# Supplementary material for: New constraints on Ti diffusion in quartz and the priming of silicic volcanic eruptions
Source: Nat Commun. 2023 Jul 17;14:4277. doi: 10.1038/s41467-023-39912-5 (PMC10352339; doi:10.1038/s41467-023-39912-5)
Supplement: Supplementary file 1 — Supplementary Information [file 41467_2023_39912_MOESM1_ESM.pdf]

Supplementary Information for

New constraints on Ti diffusion in quartz and the priming of silicic volcanic eruptions

Andreas Audétat, Axel Schmitt, Raphael Njul, Megan Harris, Anastassia Borisova, Yongjun Lu

Correspondence to: andreas.audetat@uni-bayreuth.de

CONTENTS

1. Investigated samples . . . . p. 2

2. Results from Mt. Pinatubo
2.1. Quartz textures . . . . p. 3
2.2. Age and chemistry of the zircon inclusions . . . . p. 3
2.3. Quartz CL intensity vs. trace element content . . . . p. 8
2.4. Thermobarometry . . . . p. 9
2.5. Extracted diffusion coefficients . . . . p. 12
2.6. Experimental data point at 1600 °C . . . . p. 13
2.7. Origin of the Ti-rich rims . . . . p. 14
2.8. Titanium diffusion timescales . . . . p. 15

3. Results from other locations
3.1. Quartz textures . . . . p. 16
3.2. Thermobarometry . . . . p. 17
3.3. Titanium diffusion timescales . . . . p. 18

4. References . . . . p. 18

1. Investigated samples

An overview of the investigated samples and related references is provided in Supplementary Data 4 (Table 1). Samples of the crystal-rich, white dacite of the Plinian June 15, 1991 eruption at ***Mt. Pinatubo*** were obtained from two sources: one sample (from which all quartz crystals named "Qtz..." were extracted) was provided by Michel Pichavant, whereas samples 8A, 8B and 8D stem from a profile through the earliest fall pumices collected in the Maloma River Valley (site #17 in Fig. 24 of (ref [1])), with 8A representing the base of the profile and 8D the top. The rhyolitic, crystal-rich (~50 vol% crystals) 25 Ma old ***Amalia Tuff*** (New Mexico, USA) was sampled 40 km WSW of the Questa source caldera. From the 1.3 Ma old ***Bandelier Tuff*** (New Mexico, USA), a rhyolitic sample from the last-erupted, uppermost Tshirege member collected 17 km SE of the Valles source caldera was investigated. The dacitic, crystal-rich (~50 vol% crystals) sample of the monotonous, 31 Ma old ***Cottonwood Wash Tuff*** (Utah, USA) was collected ~75 km NE of the inferred Indian Peak source caldera. The sample of the crystal-rich (~30 vol% crystals), rhyolitic, 35 Ma old ***Tunnel Spring Tuff*** (Utah, USA) was collected at Crystal Peak. The ***Hiko Tuff*** (19 Ma old, Nevada, USA) sample is a densely welded, crystal-rich (~40 vol% crystals) rhyolite sampled 35 km WSW of the Delamar source caldera of the Caliente Caldera Complex. The ***Lassen*** sample is a quartz grain separate from a pyroclastic flow or proximal fall deposit associated with the 41 ka old rhyodacite domes of Sunflower Flat next to Chaos Crags. The welded, crystal-rich (~30 vol% crystals), rhyolitic sample of the 10 Ma old ***Rainbow Mountain Tuff*** sequence (Nevada, USA) was collected near Beatty ca. 5 km SW of the Timber Mt. source caldera. The ***Toba Tuff*** sample (75 ka, Indonesia) is sample NMNH 116398-4 of the Smithsonian Museum of Natural History. The origin of the dark, welded tuff (~20 vol% crystals) is loosely described as "near Haranggaol, Lake Toba, Indonesia", but according to the phenocryst mineralogy and composition of melt inclusions in quartz it stems from the Younger Toba Tuff (YTT).

2. Results from Mt. Pinatubo

2.1. Quartz textures

Thirty-six quartz phenocrysts from Mt. Pinatubo were mapped by CL. All of them are strongly rounded, and all except for two crystals show CL-bright rims. The CL-bright rims make up usually less than 10% of the total crystal diameter (in some cases even only 1-2%), and the fact that grew discordantly over older growth zones that are cut by rounded surfaces suggests that their formation was preceded by a stage of quartz dissolution. Cathodoluminescence images of the 18 quartz phenocrysts from which zircon inclusions were dated are shown in Supplementary Data 2. Contacts between zones of contrasting luminosity are generally distinctly more blurred in the quartz cores than at the core–rim contact. In some crystals (e.g., 8Aa-8, 8Aa-15, QtzIIa-8, QtzIIb-10, QtzV-18 and QtzX-3) the contacts become gradually sharper towards the rim. Most crystals record several stages of quartz dissolution and subsequent precipitation of more Ti-rich quartz, but the last stage is typically by far the most prominent one. Exceptions are e.g., grains 8Aa-8 and 8Aa-15, which record also a prominent earlier stage of quartz dissolution and subsequent growth of Ti-rich quartz.

2.2. Age and chemistry of zircons

In addition to the 18 zircon inclusions in quartz phenocrysts that were dated by U-Th disequilibrium age dating, 55 U-Th age data were obtained on 31 zircon microphenocrysts that have previously been U-Pb age dated[2]. Furthermore, 10 zircon inclusions within quartz phenocrysts were analyzed for trace elements by LA-ICP-MS. Both zircon inclusions and zircon microphenocrysts cover the same age range between c. 25 ka and secular equilibrium (>350 ka) (Supplementary Fig. 1; Supplementary Data 4 (Table 2)). Zircon inclusions, however, have generally higher U and Th contents and lower U/Th ratios than the zircon microphenocrysts (Supplementary Fig. 2; Supplementary Data 4 (Table 3)). This correlates with higher Ce and Yb abundances in zircon inclusions relative to the zircon microphenocrysts determined by LA-ICP-MS (Supplementary Data 4 (Table 3)), both typically incompatible elements in intermediate magmas.

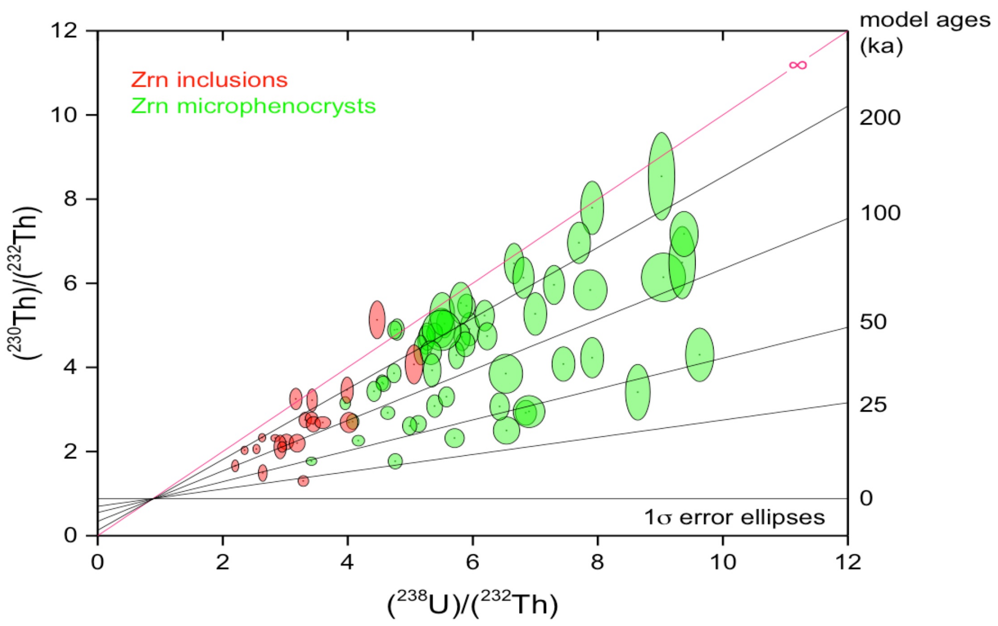

Supplementary Fig. 1. Model ages of zircon inclusions in quartz phenocrysts vs. zircon microphenocrysts extracted from the dacitic whole-rock.

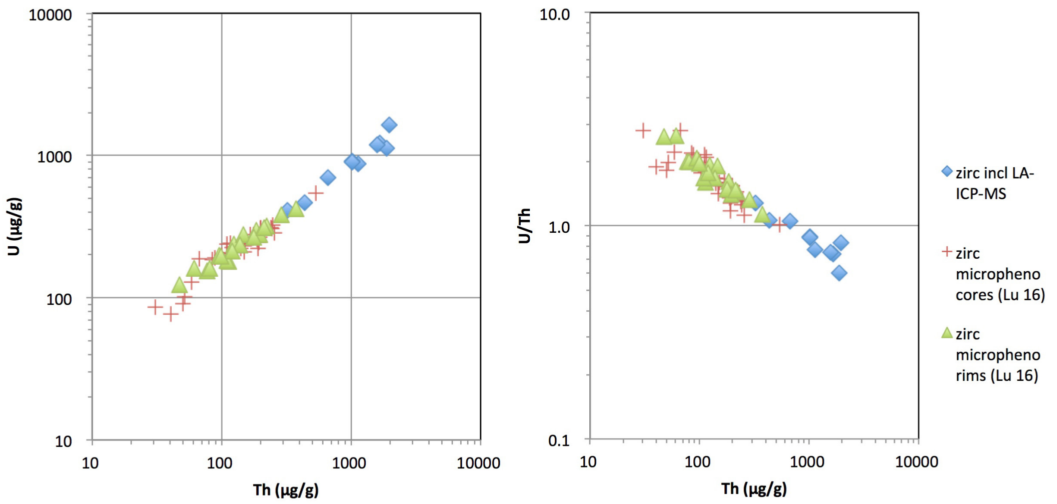

Supplementary Fig. 2. Uranium vs Th concentrations and U/Th ratios in zircon inclusions versus zircon microphenocrysts. Uranium and Th contents of zircon microphenocrysts were taken from (ref [2]).

Within the zircon microphenocrysts, the overall ranges of U, Th concentrations and U/Th ratios in cores versus rims overlap. However, if one looks at individual zircon grains, then most of the rims have higher U, Th and lower U/Th than the cores (Supplementary Fig. 3; Supplementary Data 4 (Table 3)). This observation suggests that the variations in U, Th and U/Th are caused by magmatic fractionation.

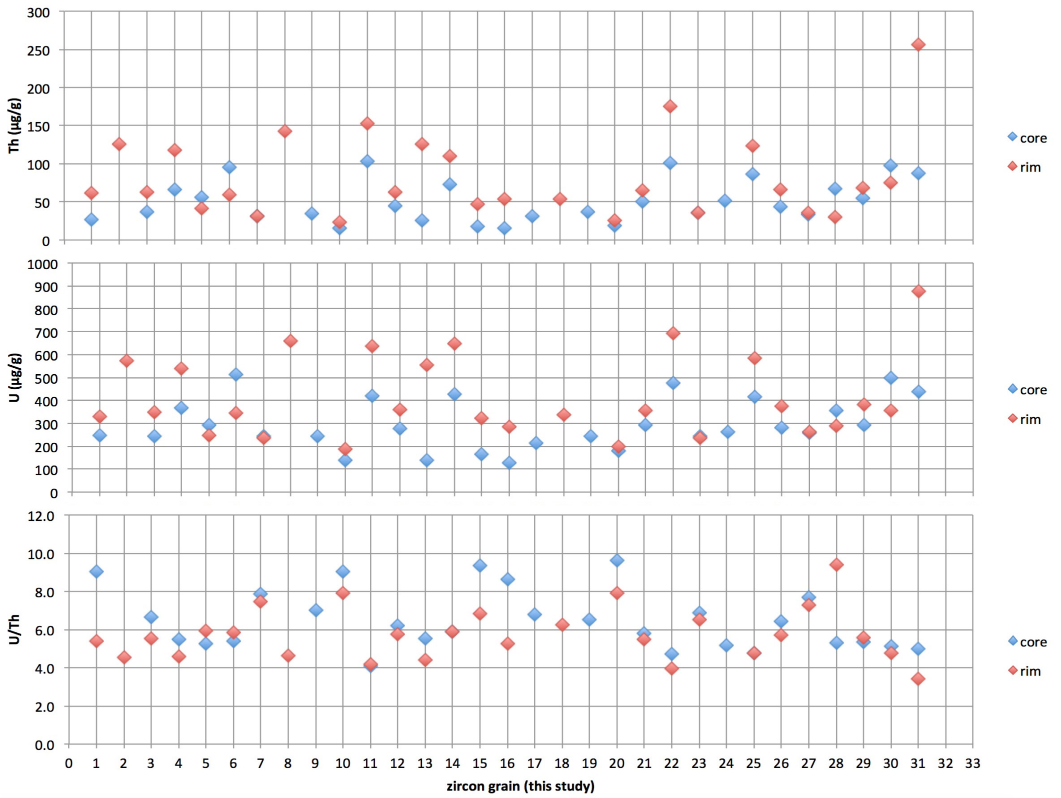

Supplementary Fig. 3. Uranium, Th concentrations and U/Th ratios in cores vs. rims of zircon microphenocrysts.

The above interpretation is supported by the trace element contents of the melt inclusions in quartz phenocrysts compared to the whole-rocks. The melt inclusions contain on average 2-3 times more Cs, Th and U than the whole-rocks (Supplementary Fig. 4; Supplementary Data 4 (Table 4)), showing that not only Cs (ref [3]) but also Th and U behaved rather incompatibly during magma fractionation. The 2-3-fold increase of these elements fits with phase equilibria experiments performed on the Mt. Pinatubo dacite[4], which suggest that at aqueous fluid saturation[5] quartz started to precipitate after the dacitic magma had reached a crystallinity of ~60 wt%.

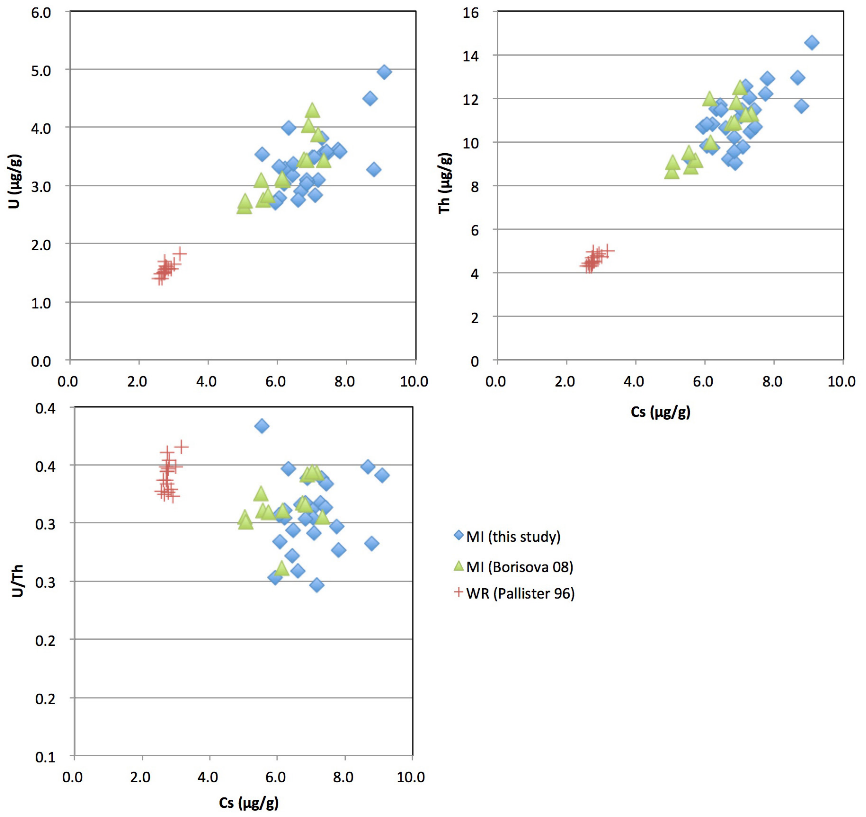

Supplementary Fig. 4. Uranium, Th concentrations and U/Th ratios in melt inclusions versus whole-rocks. The whole-rock values are from (ref [6]). Literature data of melt inclusions are from (ref [7]).

Uranium seems to have been slightly more compatible than thorium, resulting in ca. 20% lower U/Th ratios in the melt inclusions compared to the whole-rocks. Although these U-Th trends shown by melt inclusions vs. whole-rocks qualitatively agree with those recorded in the zircon inclusions vs. zircon microphenocrysts, the absolute changes are far larger in the latter, with the zircon inclusions containing in average ca. 5 times more U and ca. 10 times more Th than the zircon microphenocrysts. This calls for the involvement for some additional process, such as an increase in the $D_U^{zircon/melt}$ and $D_{Th}^{zircon/melt}$ during magma fractionation. Published partitioning data obtained from experiments[8] and natural samples[9,10] indeed suggest that the partition coefficients increase with decreasing temperature, but there is no consensus regarding the magnitude (ranging from a factor of two per 100 °C to a factor of 10 per 100 °C). For this reason, we determined $D_U^{zircon/melt}$ and $D_{Th}^{zircon/melt}$ partition coefficients in five silicic magmas by analyzing small, quartz-hosted zircon inclusions and nearby melt inclusions by LA-ICP-MS. The results (Supplementary Fig. 5; Supplementary Table 1) suggest that both $D_U^{zircon/melt}$ and $D_{Th}^{zircon/melt}$ strongly increase with decreasing temperature, and that $D_{Th}^{zircon/melt}$ does so more strongly (increasing by a factor of ca. 8 between 800 °C and 700 °C; whereas $D_U^{zircon/melt}$ increases by a factor of ca. 3.5).

Supplementary Table 1 Zircon/melt partition coefficients obtained from coexisting zircon inclusions and melt inclusions in quartz phenocrysts

Sample name	melt SiO_2 content (wt%)	T_{zirc} MI WH83 (°C)[1]	ASI MI	n MI	n zirc	$D_U^{zirc/melt}$	$D_{Th}^{zirc/melt}$
Upper Bandelier Tuff	77	820 ± 10	1.00	4	9	86 ± 35	23 ± 12
Hideaway Park tuff	77	760 ± 10	1.04	3	3	129 ± 69	23 ± 4
Lordsburg rhyolite	76	680 ± 10	1.05	7	11	370 ± 214	223 ± 147
Kos granite enclave	78	720 ± 20	1.13	3	4	341 ± 104	155 ± 55
Pinatubo dacite	78	714 ± 10	1.05	27	10	315 ± 98	124 ± 45

1) Zircon saturation temperature from melt inclusions, using the model of (ref [11])

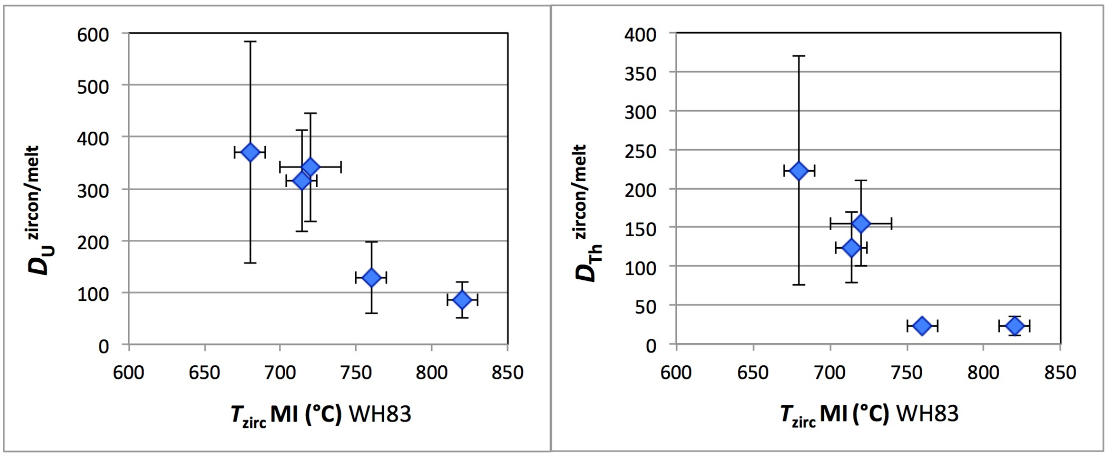

Supplementary Fig. 5. Change in the zircon/melt partition coefficient of U and Th as a function of temperature according to LA-ICP-MS measurements of pairs of zircon inclusions + melt inclusions in quartz phenocrysts from five different silicic rocks (Pinatubo dacite; Upper Bandelier Tuff, Hideaway Park Tuff[12], Lordsburg rhyolite[13], and a granitic enclave in the Kos Plateau Tuff[13]). The formation temperatures were estimated based on zircon saturation thermometry on the melt inclusions using the calibration of (ref [11]).

These increases in $D_U^{zircon/melt}$ and $D_{Th}^{zircon/melt}$, together with the 2-3-fold increase in the U and Th content of the residual liquid, explain the in average ca. 5 times higher U concentrations and ca. 10 times higher Th concentrations (and, thus, ca. two-fold lower U/Th-ratios) in the zircon inclusions compared to zircon microphenocrysts. The fact that only little overlap exists in the U, Th contents of zircon microphenocrysts versus zircon inclusions can be explained by the exponentially decreasing Zr solubility with decreasing temperature. Based on (i) the Zr content of the dacitic whole-rock (108±5 µg/g), (ii) the change of the major element composition of the residual melt during magma fractionation[4], and (iii) the dependence of the zircon solubility on temperature and melt composition[11], the onset of zircon precipitation can be estimated at ~800 °C. Due to the exponentially decreasing Zr solubility in the silicate melt the amount of zircon that precipitated between ~800 °C and ~730 °C was about nine times as high as the amount that precipitated between ~730 °C and 700 °C, with the latter representing the typical formation temperature of the zircon inclusions within quartz phenocrysts. In a spherical zircon microphenocryst

that started to crystallize at 800 °C, this last 10% crystallization volume would produce a rim that makes up only ~3% of the entire grain diameter. In other words, even if both cores and rims in the zircon microphenocrysts have been analyzed, the "rims" contained none or only a very small amount of the material that corresponds to the zircon inclusions.

2.3. Quartz trace element content vs. CL intensity

All quartz analyses of Mt. Pinatubo are provided in Supplementary Data 4 (Table 5). A crucial requirement for the use of CL grayscale profiles as proxy for Ti diffusion profiles is that the CL intensity correlates linearly with the Ti content of the quartz. Such a relationship has previously been demonstrated in studies on magmatic quartz phenocrysts[14-20], whereas in hydrothermal quartz the situation is more complex, at least at temperatures ≤400 °C[21,22]. For magmatic quartz, a correlation between quartz Ti content and CL intensity has been observed for both panchromatic CL images and hyperspectral images focused on the blue emission band at ~450 nm (2.7 eV), which is related to Ti concentration[16,18,19,23-25]. A strong correlation between quartz Ti-content and panchromatic CL intensity is observed also in the present study (Supplementary Fig. 6; Supplementary Data 4 (Table 5)), and the profiles measured in panchromatic CL images are within uncertainty the same as those measured in 500 nm shortpass-filtered (i.e., Ti-based) CL images (Methods section ; Supplementary Data 1).

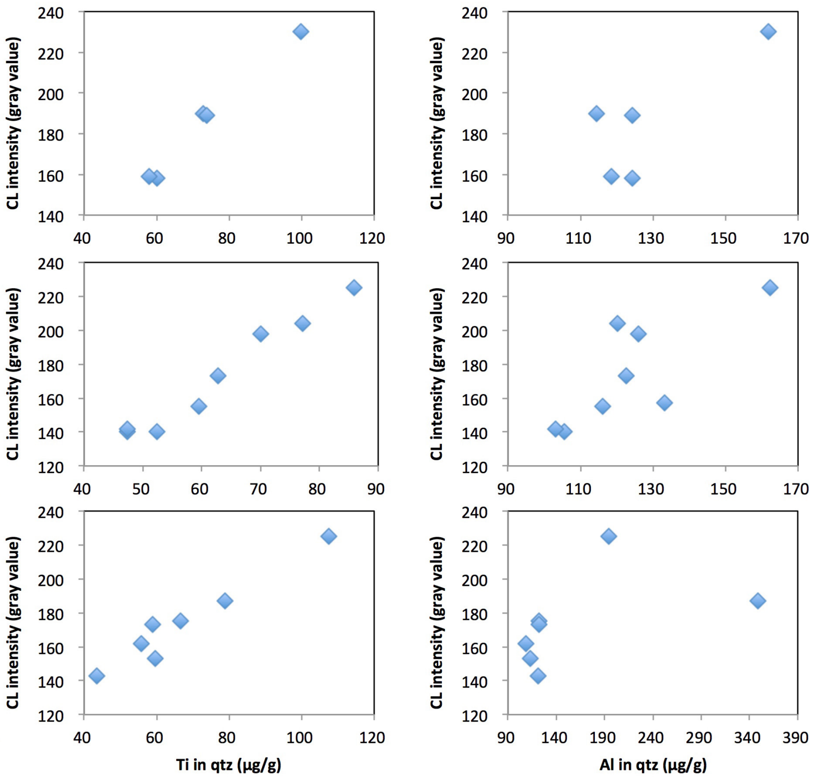

Supplementary Fig. 6. Correlation between the panchromatic CL intensity and the Ti and Al abundances in quartz measured by EPMA in sample Da-9 (top), sample Da-15 (middle), and sample Da-16 (bottom). In all cases, the CL intensity correlates well with the quartz Ti content, but less so with the quartz Al content.

Generally, Ti concentrations measured by EPMA correlate well with Ti concentrations measured by LA-ICP-MS (Supplementary Fig. 7; Supplementary Data 4 (Table 5)), for which reason the measured Ti concentrations are considered robust. Deviations in the Ti concentrations obtained from adjacent EPMA and LA-ICP-MS spots are at least partly due to the different sampling volumes of the two techniques.

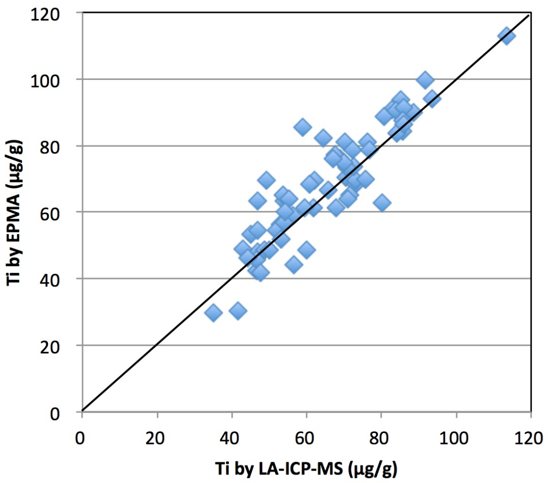

Supplementary Fig. 7. Titanium concentrations in quartz measured at adjacent spots by LA-ICP-MS vs EPMA.

2.4. Thermobarometry

Application of zircon saturation thermometry is justified by the presence of numerous zircon inclusions throughout the quartz phenocrysts. Supplementary Fig. 8 shows the abundance of zircon inclusions at a single focal depth in quartz phenocryst QtzIIa-2 as an example.

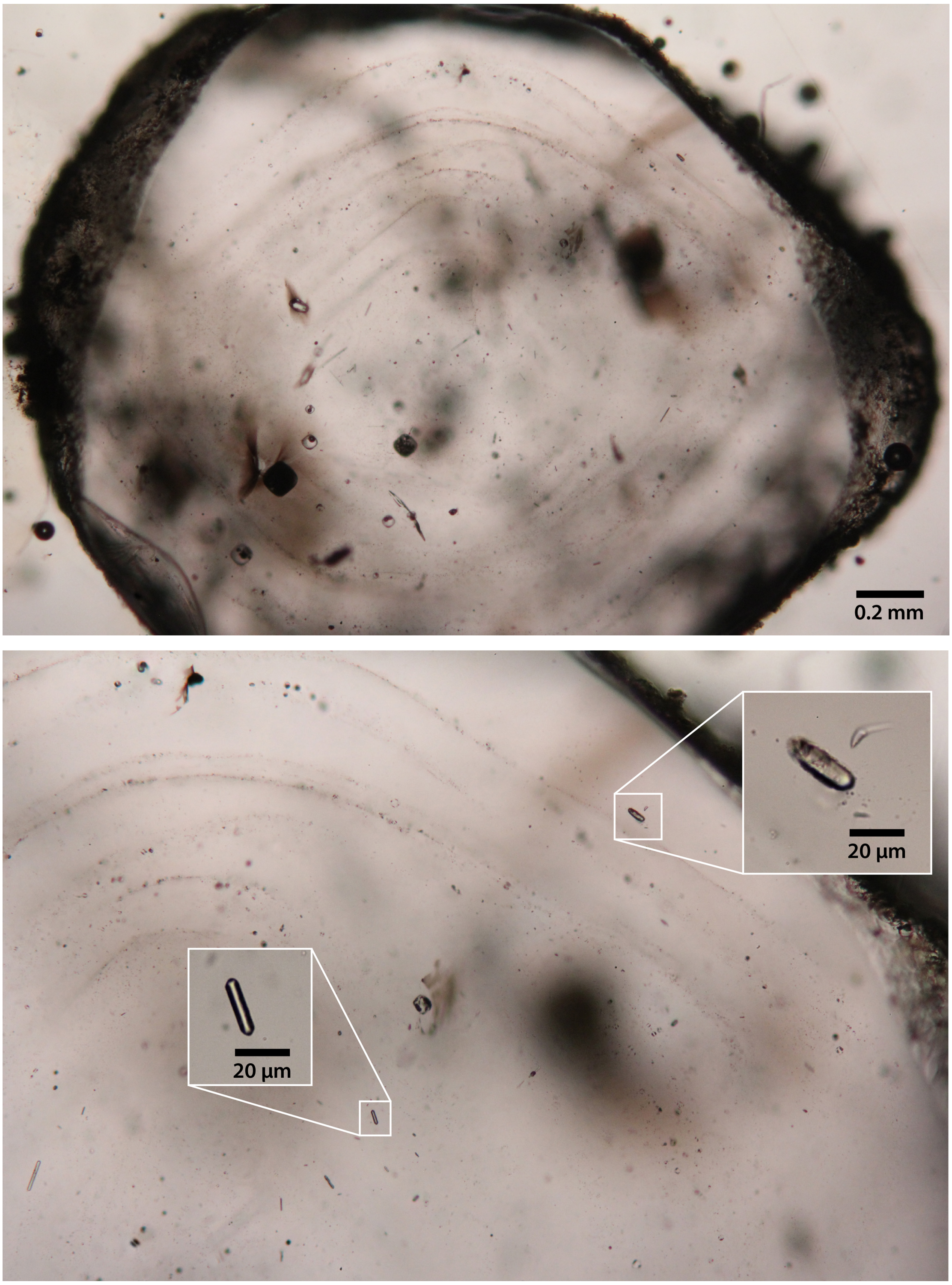

Supplementary Fig. 8. Transmitted-light photomicrograph of quartz phenocryst QtzIIa-2, showing numerous zircon inclusions at a single focal depth. Based on their high birefringence and tests done by Raman spectroscopy, virtually all needle-shaped inclusions are zircons.

Quartz crystallization pressures were calculated with the approach of (ref [26]), i.e., from the zircon saturation temperature of melt inclusions (using the model of (ref [11])), the Ti content of the surrounding host quartz (using the TitaniQ model of (ref [27])) and

the Ti-in-melt solubility model of (ref [28])). The results are in excellent agreement with the independent pressure constraints of 200±20 MPa, as they plot all within a narrow range of 200±40 MPa, except for a single quartz core that seems to have appears to have grown in a deeper magma chamber at ~300 MPa (Supplementary Fig. 9; Supplementary Data 4 (Table 6)).

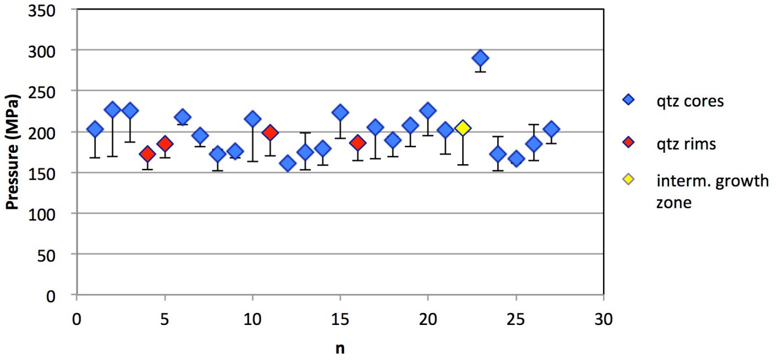

Supplementary Fig. 9. Pressures of quartz crystallization calculated from the zircon saturation temperatures of melt inclusions (using the model of (ref [11])), the TitaniQ model of (ref [27])), and the Ti-in-melt solubility model of (ref [28])). The blue data points represent quartz cores; the red data points quartz rims, and the yellow data point a Ti-rich growth zone within a quartz core. The error bars show the effect of varying temperature by ±30 °C. In principle, lowering the temperature results in higher calculated pressures (and vice versa) because the effect on $a\text{TiO}_2$ is larger than the effect on Ti solubility in quartz itself. However, if $a\text{TiO}_2$ is already close to unity (as is the case for the Pinatubo dacite), then $a\text{TiO}_2$ can raise only slightly to the maximum value of 1.0, and the effect on Ti solubility in quartz become dominant, resulting in lower calculated pressures. This is why positive error bars are absent on most data points (i.e., both 30 °C higher and 30 °C lower crystallization temperatures result in lower calculated crystallization pressures).

If the TitaniQ model of (ref [29]) is used instead of the one of (ref [27]), then most of the calculated pressures turn out at 900–1000 MPa, i.e., far higher than the independent pressure constraints. An important piece of information provided by Supplementary Fig. 9 is that the quartz cores and the quartz rims grew at similar pressures, which speaks against significant magma transport between these two stages, and, therefore, for in-situ growth of the quartz rims after the last magma rejuvenation event (see also below).
The zircon saturation temperatures obtained from the melt inclusions correlate strongly with the Ti content of the surrounding host quartz (Fig. 4 of the main text; Supplementary Fig. 10 below), suggesting that the quartz Ti content reflects at least partly variations in temperature. However, as mentioned above, it can also reflect variations in $a\text{TiO}_2$ (and potentially variations in the quartz growth rate, although in silicate melts the quartz growth rates may be generally slow enough to attain equilibrium). In fact, the TitaniQ solubility curve in quartz calculated for a constant

pressure of 200 MPa and a constant $a\mathrm{TiO_2}$ of 0.83 is distinctly steeper than the actual fit (Supplementary Fig. 10), substantiating the need for independent temperature constraints for such correlations. Application of the correlation equation in Supplementary Fig. 10 to all quartz analyses performed on the Mt. Pinatubo samples suggests that the quartz cores crystallized at temperatures of 680-730 °C, whereas the rims crystallized mostly at 720-745 °C (Supplementary Data 4 (Table 6)). Notice that the former include analyses of CL-bright growth zones that formed in response to previous heating events.

The thermal response of a magma mush to a rejuvenation event is characterized by a rapid temperature increase that is immediately followed by a rapid temperature decrease[30-32], and quartz can dissolve or re-precipitate relatively quickly in response to temperature changes. Consequently, the maximum temperatures that were reached during heating events should be similar to those recorded by the maximum Ti contents of the CL-bright growth zones, and the time spans that were spent at these high temperatures should have been far shorter than those that were spent at lower temperatures. The average magma storage temperature reconstructed from melt inclusion compositions and experimental constraints should thus be representative for deriving Ti diffusivities from the obtained zircon ages.

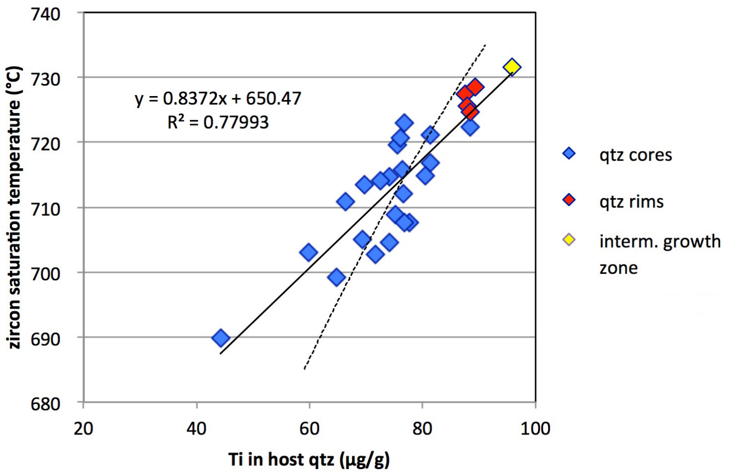

Supplementary Fig. 10. Zircon saturation temperatures of melt inclusions (calculated according to the model of (ref [11])) versus Ti concentration in the host quartz. The stippled line shows the calculated Ti solubility in quartz for a constant pressure of 200 MPa and a constant $a\mathrm{TiO_2}$ of 0.83, based on the TitaniQ formulation of (ref [27]).

2.5. Extracted diffusion coefficients

The $\log_{10}Dt$ (m^2) values extracted from the CL grayscale profiles next to the 18 dated zircon inclusions in quartz phenocrysts are listed in Supplementary Data 4 (Table 7) and are shown in Fig. 2a of the main text. From these values and the ages of the zircon inclusions, $\log_{10}D$ (m^2/s) diffusion coefficients were derived by subtracting the

equilibration times given in $\log_{10}t$ (s) from the corresponding $\log_{10}Dt$ (m^2) values (m^2) (Fig. 2b; Supplementary Data 4 (Table 7)).

2.6. Experimental data point at 1600 °C

Experiment TiDi01 of (ref [33]) was performed on a natural quartz phenocryst from the Upper Bandelier Tuff that contained a relatively sharp-bordered Ti-rich rim, similar to the quartz phenocrysts from Mt. Pinatubo. One half of the quartz phenocrysts was mapped in CL without further treatment, providing a $\log_{10}Dt$ value of -12.30±0.01 m^2 at the core–rim contact (Supplementary Fig. 11). The other half was sealed together with fine SiO_2 powder into a Pt capsule and equilibrated for 75 hours at 1600 °C and 2 GPa in a piston cylinder press. After this treatment the core–rim contact had significantly broadened to a $\log_{10}Dt$ value of -11.82±0.01 m^2 (Supplementary Fig. 11). From the difference between these two values and the experimental duration of 75 hours, a $\log_{10}D$ value of -17.36 m^2/s at 1600 °C can be calculated. All other data points of (ref [33]) were obtained on synthetic starting materials, which apparently caused problems due to either unrealistically high Ti concentrations (2000–3000 µg/g), or due to anomalous behavior in CL (the CL intensities inverted at high magnification).

Untreated half of the quartz phenocryst:

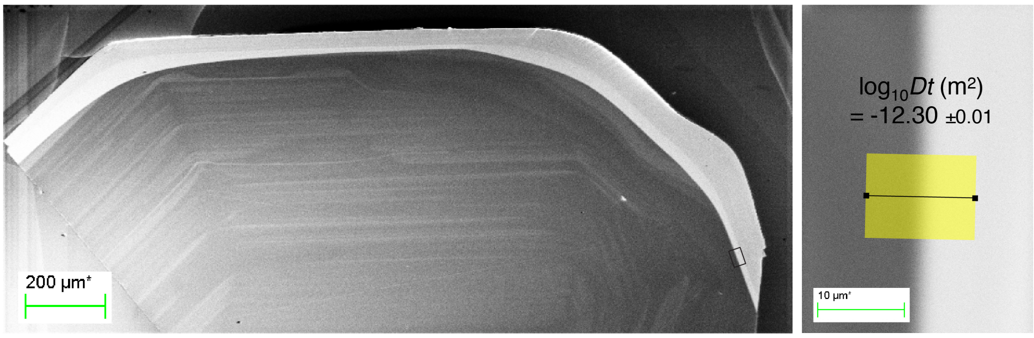

The other half of the same phenocryst, after 75 hrs at 1600 °C / 2.0 GPa:

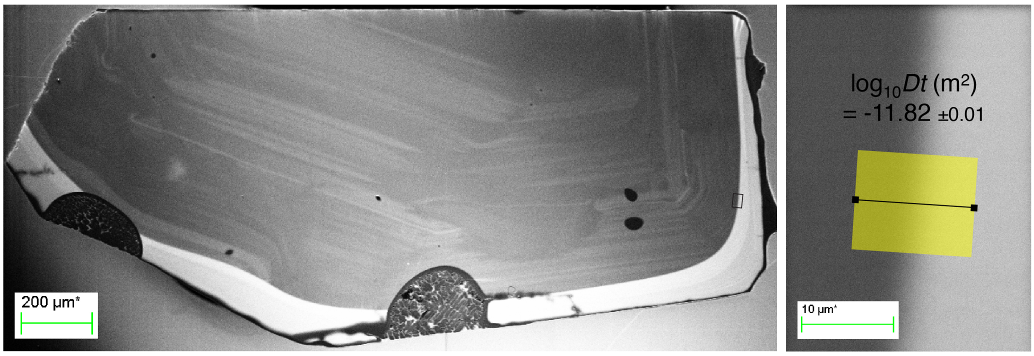

Supplementary Fig. 11. Experimental determination of the Ti diffusion rate in a naturally zoned quartz phenocrysts from the Bandelier Tuff at 1600 °C, 2 GPa (experiment TiDi01 of (ref [33])). The change from $\log_{10}Dt$ = -12.30 to -11.82 m^2 within 75 hours experimental duration suggests a $\log_{10}D$ value of -17.36 m^2/s.

2.7. Origin of the Ti-rich rims

Titanium-rich rims on quartz phenocrysts are usually interpreted to have formed as a consequence of heating associated with a magma recharge event[14,15,34,35], although alternative explanations such as faster growth rates[36] or decompression-driven growth[37] have been proposed. At Mt. Pinatubo, melt inclusions analyzed within Ti-rich rims show higher zircon saturation temperature and tend to have higher contents of compatible elements (e.g., Mg, Ti, Ba, Sr) than melt inclusions within the quartz cores (Supplementary Fig. 10, 12; Supplementary Data 4 (Table 6)). Similar (but more pronounced) geochemical trends have previously been documented for zoned quartz phenocrysts from the Bandelier Tuff and the Tunnel Spring Tuff[26]. Most rims formed after a stage of major quartz resorption, as indicated by the fact that growth zonations of the crystal interiors are cut discordantly by rounded surfaces (e.g., Mt. Pinatubo crystals 8Ab-3, 8B-11, 8Da-1, 8Da-9, 8Da-15, 8Da-16, 8Db-4 shown in Supplementary Data 2; most crystals from the Amalia Tuff, the Bandelier Tuff, the Hiko Tuff, the Lassen volcanics, the Rainbow Mt. Tuff, and the Toba Tuff shown in Supplementary Data 3). The combined evidence and the identical pressures reconstructed for quartz rims and quartz cores strongly supports the traditional view that the Ti-rich quartz rims formed in response to a magma recharge event. In the traditional view, this magma recharge event is considered to have triggered the eruption. However, this poses a problem: if hot magma was required to partially re-melt a cold crystal mush to render it eruptible, why should some quartz precipitate again prior to the eruption? Quartz precipitation requires magma cooling, unless there was a decrease in pressure or a decrease in the activity of H_2O (aH_2O). A decrease in pressure can be ruled out because TitaniQ pressure obtained from the quartz rims are identical to those obtained from the quartz cores (Supplementary Fig. 9). A decrease in aH_2O is in principle possible by means of CO_2 fluxing from the recharge magma. However, it is not clear why this should have happened *after* the heating (which caused the strong quartz dissolution prior to the growth of Ti-rich quartz), and not *simultaneously*. Furthermore, there is no evidence for elevated CO_2 concentrations in the silicate melt prior to the 1991 Mt. Pinatubo eruption, as also the glassy reentrants contain <20 ppm CO_2. We thus conclude that the rims formed during magma cooling.

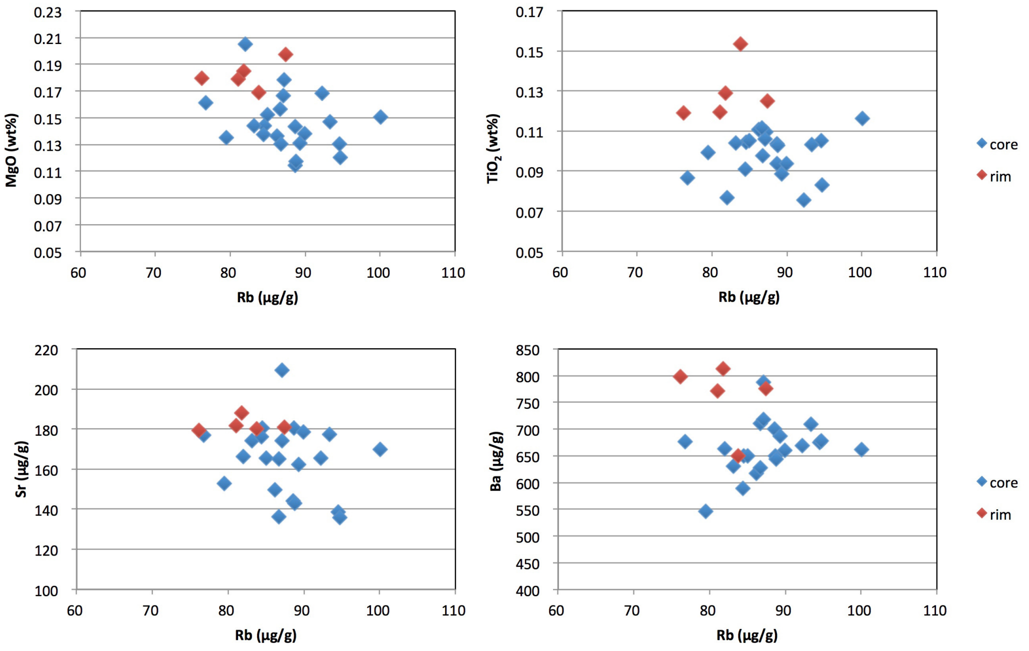

Supplementary Fig. 12. Minor- to trace element content of melt inclusions within the cores vs. rims of quartz phenocrysts. The higher concentration of compatible elements (Mg, Ti, Sr, Ba) in the rim-hosted melt inclusions suggests that the quartz rims grew after a heating event.

2.8. Titanium diffusion timescales

The newly derived Ti diffusion coefficients were used to constrain the times that elapsed since the beginning of quartz rim growth and the magma eruption in 1991. $\mathrm{Log}_{10}Dt$ (m^2) values were extracted from high-resolution CL images of core–rim contacts, and corresponding $\log_{10}D$ (m^2/s) diffusion coefficients were determined from the Ti content of the quartz rim, the Ti_{qtz} vs. T relationship shown in Supplementary Fig. 10, and equation (1) in the main text. The elapsed time (in $\log_{10}$ (s)) was then obtained by subtracting the $\log_{10}D$ (m^2/s) value from the $\log_{10}Dt$ (m^2) value. Reconstructed quartz rim temperatures vary from 712 to 745 °C (avg. of 730 °C), and calculated residence times since the beginning of quartz rim growth range from 30 to 50,000 years, with most values being between 30 and 300 years (Fig. 6 of the main text; Supplementary Data 4 (Table 8)). Strictly speaking, these are maximum values because the initial Ti distribution at the contacts may not have been a perfect step functions. However, if these diffusion profiles were dominated by pre-existing Ti concentration gradients, then the positive correlation between calculated priming times and erupted magma volumes seen among all investigated tuff occurrences (see below) is hard to explain, as there is no reason why the initial gradients at core-rim contacts of quartz crystals from large eruptions should have been less sharp than those in quartz crystals from small eruptions. Therefore, even if contributions from pre-existing gradients cannot be excluded, it appears that the profiles at quartz-rim contacts were nevertheless shaped dominantly by Ti diffusion.

In the same manner as described above, magma residence times were calculated also for diffusion profiles measured at the inner contacts of CL-bright growth zones in the interiors of the quartz phenocrysts. The obtained data are summarized in Fig. 6 of the main text and are provided in Supplementary Data 4 (Table 9). The lack of zircon inclusion ages younger than the Sacobia eruption relates to the facts that (i) most quartz of this age is preserved only in the relatively thin rims, and (ii) that our focus was on zircon inclusions associated with relatively long Ti diffusion profiles because in that case small deviations from the assumption of initial step functions do not significantly affect the calculated diffusion coefficients.

3. Results from other locations

3.1. Quartz textures

In order to obtain more estimates on the time scales between magma chamber rejuvenation and eruption, samples from 25 quartz-bearing, silicic tuffs worldwide were collected, and from each tuff 4–20 (on average 9) quartz phenocrysts were mapped by CL. Quartz phenocrysts with CL-bright rims were found in eight of these samples (Supplementary Data 4 (Table 1)): the Amalia Tuff (New Mexico, USA), the uppermost Bandelier Tuff (New Mexico, USA), the Cottonwood Wash Tuff (Utah, USA); the Tunnel Spring Tuff (Utah, USA), the Hiko Tuff (Nevada, USA), the Sunflower Flat rhyodacite at Lassen Volcano (California, USA), the Rainbow Mt. Tuff (Nevada, USA), and YTT (Sumatra, Indonesia). In the other 17 (i.e., two-thirds of the) tuffs, no quartz phenocrysts with CL-bright rims were found. However, this does not necessarily mean that in these tuffs such phenocrysts are generally absent. From thoroughly studied occurrences like the Bishop Tuff and the Bandelier Tuff it is known that only the last-erupted magma units contain rimmed quartz phenocrysts, whereas in the earlier-erupted units they are absent[38,39]. Therefore, with only 1–3 samples being available from each tuff for the present study, and only 4–20 phenocrysts being examined from each sample, it is not possible to tell with certainty whether rimmed quartz phenocrysts are absent. In some of the tuffs, nearly all investigated quartz phenocrysts showed CL-bright rims (Amalia Tuff: 12 out of 12; i.e., 12/12; Rainbow Mt. Tuff: 10/12; uppermost Bandelier Tuff: 9/13), in others they were common (Toba Tuff: 7/10; Hiko Tuff: 6/9; Tunnel Spring Tuff: 5/8; Cottonwood Wash Tuff: 5/10), and in the Sunflower Flat Rhyolite from Lassen Volcano they were rare (3/15).

Twenty-five representative CL images of these samples are shown in Supplementary Data 3. In most samples, the CL-bright rims grew discordantly over resorbed, less CL-rich cores, indicating growth after a resorption event. As at Mt. Pinatubo, rim-hosted melt inclusions in quartz phenocrysts from the Bandelier Tuff and the Tunnel Spring Tuff are more mafic and return higher zircon saturation temperatures than melt inclusions in the cores, suggesting that quartz resorption and subsequent formation of Ti-rich rims occurred in response to a magma recharge[26]. From the other tuffs no data

from rim-hosted melt inclusions are available, but it is expected that also in these samples the Ti-rich rims formed in response to magma recharge because in most crystal-rich rhyolite tuffs the compositions of the matrix is more mafic than the composition of quartz-hosted melt inclusions[13].

In the Cottonwood Wash Tuff, the Hiko Tuff and the Rainbow Mt. Tuff the quartz phenocrysts commonly show stepwise increases in the CL intensity towards the rim, and the very thin rims of the Tunnel Spring Tuff were not preceded by significant quartz dissolution.

3.2. Thermobarometry

As emphasized in a previous study[14], one of the largest sources of uncertainty in the estimation of diffusion times are uncertainties in the estimated temperature. At 750 °C, for example, a temperature uncertainty of ±50 °C results in a ±fourfold change in the diffusion times calculated according to equation (1). Magma temperatures were thus constrained as accurately as possible based on published data and/or the approaches detailed below. An overview of all thermobarometric constraints that were used to estimate magma temperatures is provided in Supplementary Data 4 (Table 1), whereas melt inclusion-based zircon saturation temperatures and corresponding TitaniQ pressures are provided in Supplementary Data 4 (Table 10). A major aim of Supplementary Data 4 (Table 10) was to test different zircon solubility models. As shown by (ref [40]), a sensitive test for the reliability of zircon saturation temperatures is to calculate corresponding activities of TiO_2 ($aTiO_2$) in the melt[28,41] and compare the results with independent estimates obtained from Fe-Ti-oxides[42]. If the zircon saturation temperature is too low, then the calculated $aTiO_2$ becomes too high (commonly above unity, which is impossible). Such unreasonably high $aTiO_2$ values are obtained for most of the investigated samples if the zircon saturation temperatures are calculated based on the model of (ref [43]) or model 1 of (ref[44]), except for the Upper Bandelier Tuff and the Youngest Toba Tuff (Supplementary Data 4 (Table 10)). Following the findings of (ref [13]) and those made above for the samples from Mt. Pinatubo, the zircon saturation thermometer of (ref [11]) was used to calculate melt inclusions entrapment temperatures also for all other occurrences, except for the peralkaline Amalia tuff, for which the model of (ref [11]) is not valid. For the samples investigated from the Upper Bandelier Tuff, the Tunnel Spring Tuff, and the Hiko Tuff, a correlation between melt inclusion zircon saturation temperature and the Ti content of the surrounding host quartz could be established, which allowed estimation of quartz formation temperatures solely based on the quartz Ti content. For each sample the correlation is different because $aTiO_2$ does not stay constant during magma rejuvenation (cf. (ref [26])).

All Fe-Ti-oxide pairs that were used for Supplementary Data 4 (Table 10) passed the Mg/Mn partitioning test[45] and were recast based on the model of (ref [42]) using the online calculator at https://gitlab.com/ENKI-portal/app-fe-ti-oxide-geotherm. For the Upper Bandelier Tuff and the Youngest Toba Tuff, independent temperature

estimates were obtained from the Mo content of melt inclusions[46], as these magmas are saturated in molybdenite, magnetite and pyrrhotite, and independent fO_2 estimates available. For the Amalia Tuff, valuable constraints on temperature were obtained from experimental phase equilibria on a compositionally similar peralkaline rhyolite[47]. The Amalia Tuff seems to have been fluid-saturated, as melt inclusions and fluid inclusions (now commonly empty) coexist on the same growth zones within quartz and feldspar phenocrysts. The formation temperature of the quartz rims of the Lassen sample is poorly constrained because only zircon saturation temperatures for melt inclusions in the quartz cores are available, and because – as shown above – extrapolations based solely on the Ti content of quartz and assuming constant pressure and $a\mathrm{TiO_2}$ are dangerous. The rough estimate of 800±70 °C is based on this assumption, plus from Fe-Ti-oxides temperatures reported from slightly younger rhyodacite erupted at nearby Chaos Crags[48,49].

3.3. Titanium diffusion timescales

The full data set of the obtained Ti diffusion timescales is provided in Supplementary Data 4 (Table 11). Diffusion timescales obtained from core–rim contacts are mostly on the order of decades to thousands of years (excluding two data points from Lassen volcano, which are only on the order of years), whereas timescales obtained from growth zone contacts in the interiors of the same quartz crystals are generally 1–2 orders of magnitude longer. An exception is the Amalia Tuff, where the timescales obtained from quartz interiors are only 2–3 times longer than those obtained from the rim. Importantly, the obtained timescales correlate positively with the erupted magma volume and negatively with the magma temperature, which fits with the results of numerical models[50,51], suggesting that large and/or cool magma volumes require more time to become partially melted and reach an eruptible state than small and/or hot magma volumes. In analogy with the findings at Mt. Pinatubo, it is assumed that also in the other studied occurrences the majority of the Ti-rich quartz rims are genetically related to the eruption that produced the sampled tuff, even if they may not be representative of the recharge event that was the ultimate trigger of the eruption.

4. References

1 Hoblitt R. P., Wolfe E. W., Scott W. E., Couchman M. R., Pallister J. S. and Javier D. (1996) The pre-climactic eruptions of Mount Pinatubo, June 1991. In *Fire and mud: eruptions and lahars of the Mount Pinatubo, Philippines* (eds. C. G. Newhall and R. S. Punongbayan), 457-511. Univ. Washington Press, Seattle.

2 Lu Y.-J., Loucks R. L., Fiorentini M. L., McCuaig T. C., Evans N. J., Yang Z.-M., Hou Z.-Q., Kirkland C. L., Parra-Avila L. A. and Kobussen A. (2016) Zircon compositions as a pathfinder for porphyry Cu±Mo±Au mineral deposits In *Tethyan tectonics and metallogeny* (ed., J. P. Richards) Society of Economic Geologists Special Publication, 329-347.

3 Audétat A. and Pettke T. (2003) The magmatic-hydrothermal evolution of two barren granites: a melt and fluid inclusion study of the Rito del Medio and Cañada Pinabete Plutons in northern New Mexico (USA). *Geochim. Cosmochim. Acta* **67**, 97-121.

4 Scaillet, B. & Evans, B. W. The 15 June 1991 eruption of Mount Pinatubo. I. Phase equilibria and pre-eruption P-T-fO_2-fH_2O conditions of the dacite magma. *J. Petrol.* **40**, 381-411 (1999).

5 Borisova, A. Y., Pichavant, M., Beny, J.-M., Rouer, O. & Pronost, J. Constraints on dacite magma degassing and regime of the June 15, 1991, climactic eruption of Mount Pinatubo (Philippines): New data on melt and crystal inclusions in quartz. *J. Volc. Geotherm. Res.* **145**, 35-67 (2005).

6 Pallister, J. S., Hoblitt, R. P., Meeker, G. P., Knight, R. J. & Siems, D. F. Magma mixing at Mount Pinatubo: petrographic and chemical evidence from the 1991 deposits. In *Fire and mud: eruptions and lahars of the Mount Pinatubo, Philippines* (eds Newhall C. G & Punongbayan, R. S.) 687-731 (Univ. Washington Press, 1996).

7 Borisova A. Y., Freydier R., Polvé M., Salvi S., Candaudap F. and Aigouy T. (2008) *In situ* multi-elemental analysis of the Mount Pinatubo quartz-hosted melt inclusions by NIR femtosecond laser ablation-inductively coupled plasma-mass spectrometry. *Geostand. Geoanal. Res.* **32**, 209-229.

8 Rubatto D. and Hermann J. (2007) Experimental zircon/melt and zircon/garnet trace element partitioning and implications for the geochronology of crustal rocks. *Chem. Geol.* **241**, 38-61.

9 Kirkland C. L., Smithies R. H., Taylor R. J. M., Evans N. and McDonald B. (2015) Zircon Th/U ratios in magmatic environs. *Lithos* **212-215**, 397-414.

10 Claiborne L. L., Miller C. F., Gualda G. A. R., Carley T. L., Covey A. K., Wooden J. L. and Fleming M. A. (2018) Zircon as magma monitor: robust, temperature-dependent partition coefficients from glass and zircon surface and rim measurements from natural systems In *Microstructural geochronology: planetary records down to atom scale* (eds. D. E. Moser, F. Y. Corfu, J. R. Darling, S. M. Reddy and K. Tait) *Geophysical Monograph* **232**, doi.org/10.1002/9781119227250.ch1.

11 Watson, B. E. & Harrison, M. T. Zircon saturation revisited: temperature and composition effects in a variety of crystal magma types. *Earth Planet. Sci. Lett.* **64**, 295-304 (1983).

12 Mercer C. N., Hofstra A. H., Todorov T. I., Roberge J., Burgisser A., Adams M. C. and Cosca M. (2015) Pre-eruptive conditions of the Hideaway Park topaz rhyolite: insights into metal source and evolution of magma parental to the Henderson porphyry

13 Arató R. and Audétat A. (2017) Vanadium magnetite–melt oxybarometry of natural, silicic magmas: a comparison of various oxybarometers and thermometers. *Contrib. Min. Petrol.* **172**, DOI:10.1007/s00410-017-1369-6.

14 Matthews, N. E., Huber, C., Pyle, D. M. & Smith, V. C. Timescales of magma recharge and reactivation of large silicic systems from Ti diffusion in quartz. *J. Petrol.* **53**, 1385-1416 (2012).

15 Wark, D. A., Hildreth, W., Spear, F. S., Cherniak, D. J. & Watson, E. B. Pre-eruption recharge of the Bishop magma system. *Geology* **35**, 235-238 (2007).

16 Leeman, W. P. *et al.* A study of cathodoluminescence and trace element compositional zoning in natural quartz from volcanic rocks: mapping titanium content in quartz. *Microsc. Microanal.* **18**, 1322-1341 (2012).

17 Drivenes, K., Larsen, R. B., Müller, A. & Sorensen, B. E. Crystallization and uplift path of late Variscan granites evidenced by quartz chemistry and fluid inclusions: Example from the Land's End granite, SW England. *Lithos* **252-253**, 57-75 (2016).

18 Müller A., van den Kerkhof A. M., Behr H. J., Kronz A. and Koch-Müller M. (2009) The evolution of late-Hercynian granites and rhyolites documented by quartz - a review. *T. Roy. Soc. Edin. - Earth* **100**, 185-204.

19 Müller A., Wiedenbeck M., van den Kerkhof A. M., Kronz A. and Simon K. (2003) Trace elements in quartz – a combined electron microprobe, secondary ion mass spectrometry, laser-ablation ICP-MS, and cathodoluminescence study. *Eur. J. Mineral.* **15**, 747-763.

20 Smith V. C., Shane P. and Nairn I. (2010) Insights into silicic melt generation using plagioclase, quartz and melt inclusions from the caldera-forming Rotoiti eruption, Taupo volcanic zone, New Zealand. *Contrib. Min. Petrol.* **160**, 951-971.

21 Rusk B. G., Lowers H. A. and Reed M. H. (2008) Trace elements in hydrothermal quartz: relationships to cathodoluminescence textures and insights into vein formation. *Geology* **36**, 547-550.

22 Rusk B. (2012) Cathodoluminescent textures and trace elements in hydrothermal quartz. In *Quartz: deposits, mineralogy and analytics* (eds. J. Götze and R. Möckel), 307-329. Springer, Heidelberg.

23 Vasyukova, O. V., Goemann, K., Kamenetsky, V. S., MacRae, C. M. & Wilson, N. C. Cathodoluminescence properties of quartz eyes from porphyry-type deposits: implications for the origin of quartz. *Am. Mineral.* **98**, 98-109 (2013).

24 MacRae C. M., Wilson N. C. and Torpy A. (2013) Hyperspectral cathodoluminescence. *Miner. Petrol.* **107**, 429-440.

25 Götze J., Pan Y. and Müller A. (2021) Mineralogy and mineral chemistry of quartz: A review. *Mineral. Mag.* **85**, doi:10.1180/mgm.2021.72.

26 Audétat, A. Origin of Ti-rich rims in quartz phenocrysts from the Upper Bandelier Tuff and the Tunnel Spring Tuff, southwestern USA. *Chem. Geol.* **360-361**, 99-104 (2013).

27 Huang R. and Audétat A. (2012) The titanium-in-quartz (TitaniQ) thermobarometer: a critical examination and re-calibration. *Geochim. Cosmochim. Acta* **84**, 75-89.

28 Kularatne, K. & Audétat, A. Rutile solubility in hydrous rhyolite melts at 750-900 °C and 2 kbar, with application to titanium-in-quartz (TitaniQ) thermobarometry. *Geochim. Cosmochim. Acta* **125**, 196-209 (2014).

29 Thomas J. B., Watson E. B., Spear F. S., Shemella P. T., Nayak S. K. and Lanzirotti A. (2010) TitaniQ under pressure: the effect of pressure and temperature on the solubility of Ti in quartz. *Contrib. Min. Petrol.* **160**, 743-759.

30 Bachmann O. and Huber C. (2016) Silicic magma reservoirs in the Earth's crust. *Am. Mineral.* **101**, 2377-2404.

31 Rubin A. E., Cooper K. M., Till C. B., Kent A. J. R., Costa F., Bose M., Gravley D., Deering C. and Cole J. (2017) Rapid cooling and cold storage in a silicic magma reservoir recorded in individual crystals. *Science* **356**, 1154-1156.

32 Kent A. J. R. and Cooper K. M. (2018) How well do zircons record the thermal evolution of magmatic systems? *Geology* **46**, 111-114.

33 Audétat, A., Miyajima, N., Wiesner, D. & Audinot, J.-N. Confirmation of slow Ti diffusion in quartz by diffusion couple experiments and evidence from natural samples. *Geology*, doi.org/10.1130/G48785.48781 (2021).

34 Chamberlain, K. J., Morgan, D. J. & Wilson, C. J. N. Timescales of mixing and mobilisation in the Bishop Tuff magma body: perspectives from diffusion chronometry. *Contrib. Min. Petrol.* **168**, 1034 (2014).

35 Cooper, G. F., Morgan, D. J. & Wilson, C. J. N. Rapid assembly and rejuvenation of a large silicic magmatic system: Insights from mineral diffusive profiles in the Kidnappers and Rocky Hill deposits, New Zealand. *Earth Planet. Sci. Lett.* **473**, 1-13 (2017).

36 Pamukcu, A. S., Ghiorso, M. S. & Gualda, G. A. R. High‐Ti, bright‐CL rims in volcanic quartz: a result of very rapid growth. *Contrib. Min. Petrol.* **171**, doi 10.1007/s00410-00016-01317-x (2016).

37 Shamloo, H. I. & Till, C. B. Decadal transition from quiescence to supereruption: petrologic investigation of the Lava Creek Tuff, Yellowstone Caldera, WY. *Contrib. Min. Petrol.* **174**, 32 (2019).

38 Peppard, B. T., Steele, I. M., Davis, A. M., Wallace, P. J. & Anderson, A. T. Zoned quartz phenocrysts from the rhyolitic Bishop Tuff. *Am. Mineral.* **86**, 1034-1052 (2001).

39 Wilcock, J., Goff, F., Minarik, W. G. & Stix, J. Magmatic recharge during the formation and resurgence of the Valles Caldera, New Mexico, USA: evidence from quartz compositional zoning and geothermometry. *J. Petrol.* **54**, 635-664 (2013).

40 Audétat, A. Comment on "Ti-in-quartz thermobarometry and TiO2 solubility in rhyolitic melts: New experiments and parametrization" by Zhang et al. [Earth Planet. Sci. Lett. 538 (2020) 116213]. *Earth Planet. Sci. Lett.*, doi.org/10.1016/j.epsl.2021.116847 (2021).

41 Hayden, L. A. & Watson, E. B. Rutile saturation in hydrous siliceous melts and its bearing on Ti-thermometry of quartz and zircon. *Earth Planet. Sci. Lett.* **258**, 561-568 (2007).

42 Ghiorso, M. S. & Evans, B. W. Thermodynamics of rhombohedral oxide solid solutions and a revision of the Fe-Ti two-oxide geothermometer and oxygen-barometer. *Am. J. Sci.* **308**, 957-1039 (2008).

43 Boehnke, P., Watson, B. E., Trail, D., Harrison, T. M. & Schmitt, A. K. Zircon saturation re-visited. *Chem. Geol.* **351**, 324-334 (2013).

44 Shao, T., Xia, Y., Ding, X., Cai, Y. & Song, M. Zircon saturation model in silicate melts: a review and update. *Acta Geochimica* **39**, doi.org/10.1007/s11631-11019-00384-11634 (2020).

45 Bacon, C. R. & Hirschmann, M. M. Mg/Mn partitioning test for equilibrium between coexisting Fe-Ti oxides. *Am. Mineral.* **73**, 57-61 (1988).

46 Sun, W., Audétat, A. & Dolejs, D. Solubility of molybdenite in hydrous granitic melts at 800 °C, 100-200 MPa. *Geochim. Cosmochim. Acta* **131**, 393-401 (2014).

47 Scaillet, B. & MacDonald, R. Phase relations of peralkaline silicic magmas and petrogenetic implications. *J. Petrol.* **42**, 825-845 (2001).

48 Quinn E. T., 2014. Experimental determination of pre-eruptive storage conditions and continuous decompression of rhyodacite magma erupted from Chaos Crags, Lassen Volcanic Center, California. M.Sc. thesis, Humboldt State University.

49 Scruggs M. A. and Putirka K. D. (2018) Eruption triggering by partial crystallization of mafic enclaves at Chaos Crags, Lassen Volcanic Center, California. *Am. Mineral.* **103**, 1575-1590.

50 Huber C., Bachmann O. and Dufek J. (2011) Thermo-mechanical reactivation of locked crystal mushes: Melting-induced internal fracturing and assimilation processes in magmas. *Earth Planet. Sci. Lett.* **304**, 443-454.

51 Huber C., Bachmann O. and Dufek J. (2012) Crystal-poor versus crystal-rich ignimbrites: A competition between stirring and reactivation. *Geology* **40**, 115-118.
