# Supplementary material for: New constraints on Ti diffusion in quartz and the priming of silicic volcanic eruptions
Source: Nat Commun. 2023 Jul 17;14:4277. doi: 10.1038/s41467-023-39912-5 (PMC10352339; doi:10.1038/s41467-023-39912-5)
Supplement: Supplementary file 3 — Description of Additional Supplementary Files [file 41467_2023_39912_MOESM3_ESM.pdf]

Description of Additional Supplementary Files

File Name: Supplementary Data 1

Description: Cathodoluminescence images of selected quartz phenocrysts, once taken in panchromatic mode and once using a 500 nm shortpass filter, plus corresponding diffusion profiles measured at core–rim contacts.

File Name: Supplementary Data 2

Description: Cathodoluminescence images, measured Ti concentrations in quartz (yellow points = LA-ICP-MS; white points = EPMA), and diffusion profiles measured at growth zone contacts in all quartz phenocrysts investigated from Mt. Pinatubo, with one profile fitting example shown for each sample. White circles labeled MI1, MI2, MI3 show locations of analyzed melt inclusions.

File Name: Supplementary Data 3

Description: Cathodoluminescence images, Ti concentrations in quartz measured by LA-ICP-MS (yellow points), analyzed melt inclusions with calculated zircon saturation temperatures (white points), and diffusion profiles measured at core–rim contacts in selected quartz phenocrysts investigated from all other occurrences.

File Name: Supplementary Data 4

Description: Excel file containing all analytical data grouped in 11 different Tables.
