# Supplementary material for: New constraints on Ti diffusion in quartz and the priming of silicic volcanic eruptions
Source: Nat Commun. 2023 Jul 17;14:4277. doi: 10.1038/s41467-023-39912-5 (PMC10352339; doi:10.1038/s41467-023-39912-5)
Supplement: Supplementary file 4 — Supplementary Data 1 [file 41467_2023_39912_MOESM4_ESM.pdf]

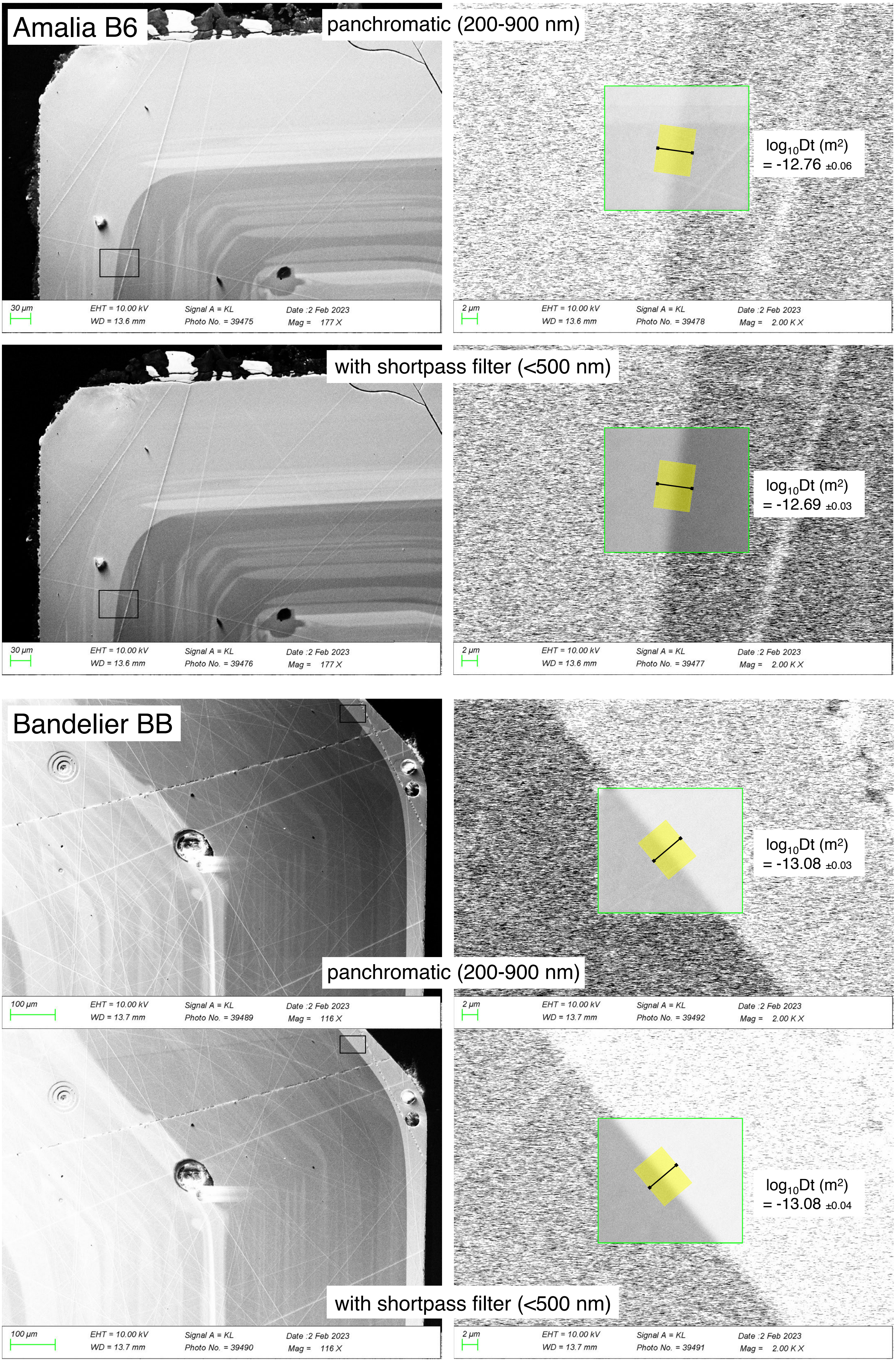

Amalia B6
panchromatic (200-900 nm)
log10Dt (m2) = -12.76 ±0.06
30 µm
EHT = 10.00 kV
WD = 13.6 mm
Signal A = KL
Photo No. = 39475
Date :2 Feb 2023
Mag = 177 X
2 µm
EHT = 10.00 kV
WD = 13.6 mm
Signal A = KL
Photo No. = 39478
Date :2 Feb 2023
Mag = 2.00 K X
with shortpass filter (<500 nm)
log10Dt (m2) = -12.69 ±0.03
30 µm
EHT = 10.00 kV
WD = 13.6 mm
Signal A = KL
Photo No. = 39476
Date :2 Feb 2023
Mag = 177 X
2 µm
EHT = 10.00 kV
WD = 13.6 mm
Signal A = KL
Photo No. = 39477
Date :2 Feb 2023
Mag = 2.00 K X
Bandelier BB
log10Dt (m2) = -13.08 ±0.03
panchromatic (200-900 nm)
100 µm
EHT = 10.00 kV
WD = 13.7 mm
Signal A = KL
Photo No. = 39489
Date :2 Feb 2023
Mag = 116 X
2 µm
EHT = 10.00 kV
WD = 13.7 mm
Signal A = KL
Photo No. = 39492
Date :2 Feb 2023
Mag = 2.00 K X
log10Dt (m2) = -13.08 ±0.04
with shortpass filter (<500 nm)
100 µm
EHT = 10.00 kV
WD = 13.7 mm
Signal A = KL
Photo No. = 39490
Date :2 Feb 2023
Mag = 116 X
2 µm
EHT = 10.00 kV
WD = 13.7 mm
Signal A = KL
Photo No. = 39491
Date :2 Feb 2023
Mag = 2.00 K X

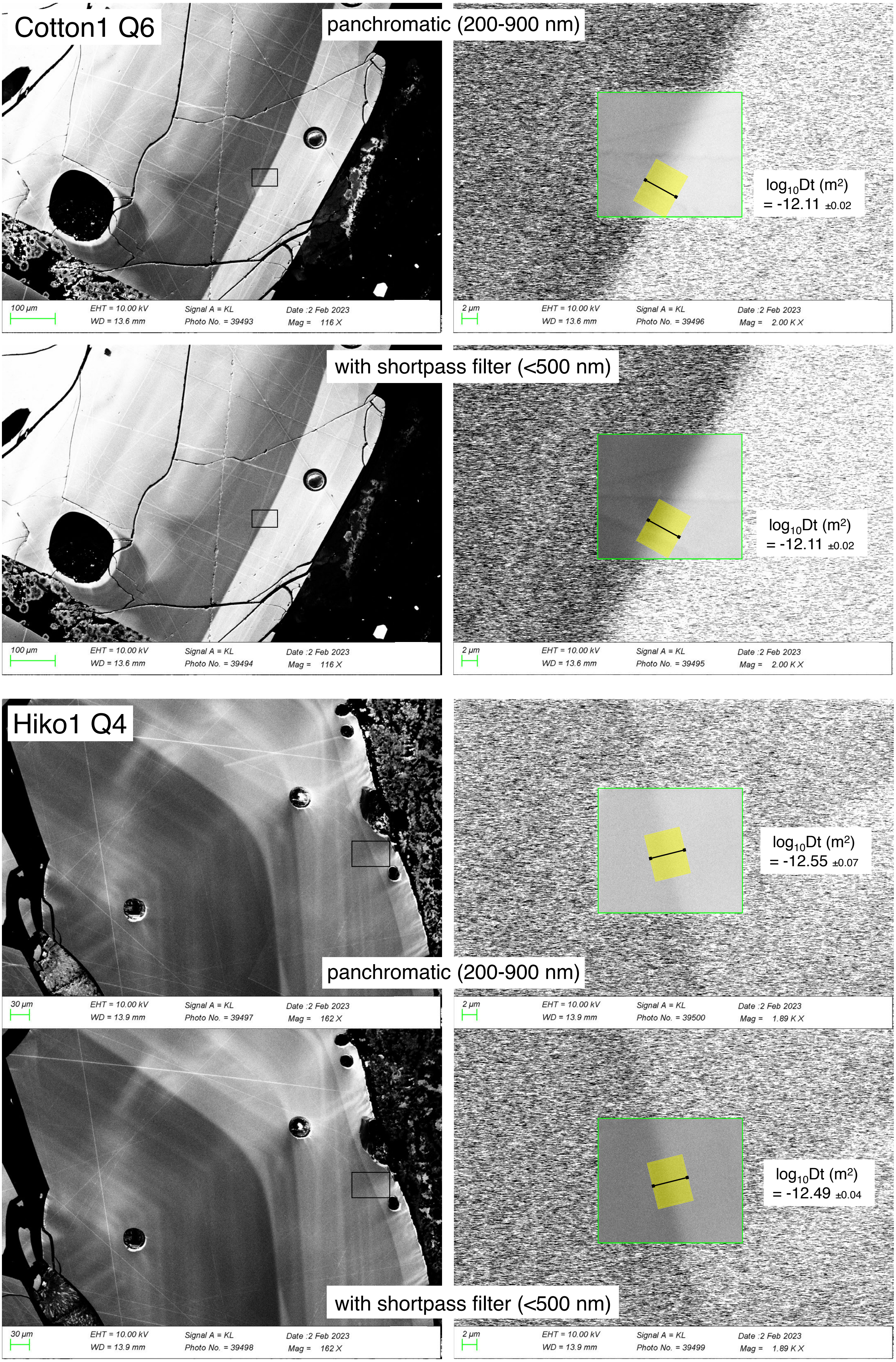
Cotton1 Q6
panchromatic (200-900 nm)
log10Dt (m2) = -12.11 ±0.02
100 µm
EHT = 10.00 kV
WD = 13.6 mm
Signal A = KL
Photo No. = 39493
Date :2 Feb 2023
Mag = 116 X
2 µm
EHT = 10.00 kV
WD = 13.6 mm
Signal A = KL
Photo No. = 39496
Date :2 Feb 2023
Mag = 2.00 K X
with shortpass filter (<500 nm)
log10Dt (m2) = -12.11 ±0.02
100 µm
EHT = 10.00 kV
WD = 13.6 mm
Signal A = KL
Photo No. = 39494
Date :2 Feb 2023
Mag = 116 X
2 µm
EHT = 10.00 kV
WD = 13.6 mm
Signal A = KL
Photo No. = 39495
Date :2 Feb 2023
Mag = 2.00 K X
Hiko1 Q4
log10Dt (m2) = -12.55 ±0.07
panchromatic (200-900 nm)
30 µm
EHT = 10.00 kV
WD = 13.9 mm
Signal A = KL
Photo No. = 39497
Date :2 Feb 2023
Mag = 162 X
2 µm
EHT = 10.00 kV
WD = 13.9 mm
Signal A = KL
Photo No. = 39500
Date :2 Feb 2023
Mag = 1.89 K X
log10Dt (m2) = -12.49 ±0.04
with shortpass filter (<500 nm)
30 µm
EHT = 10.00 kV
WD = 13.9 mm
Signal A = KL
Photo No. = 39498
Date :2 Feb 2023
Mag = 162 X
2 µm
EHT = 10.00 kV
WD = 13.9 mm
Signal A = KL
Photo No. = 39499
Date :2 Feb 2023
Mag = 1.89 K X

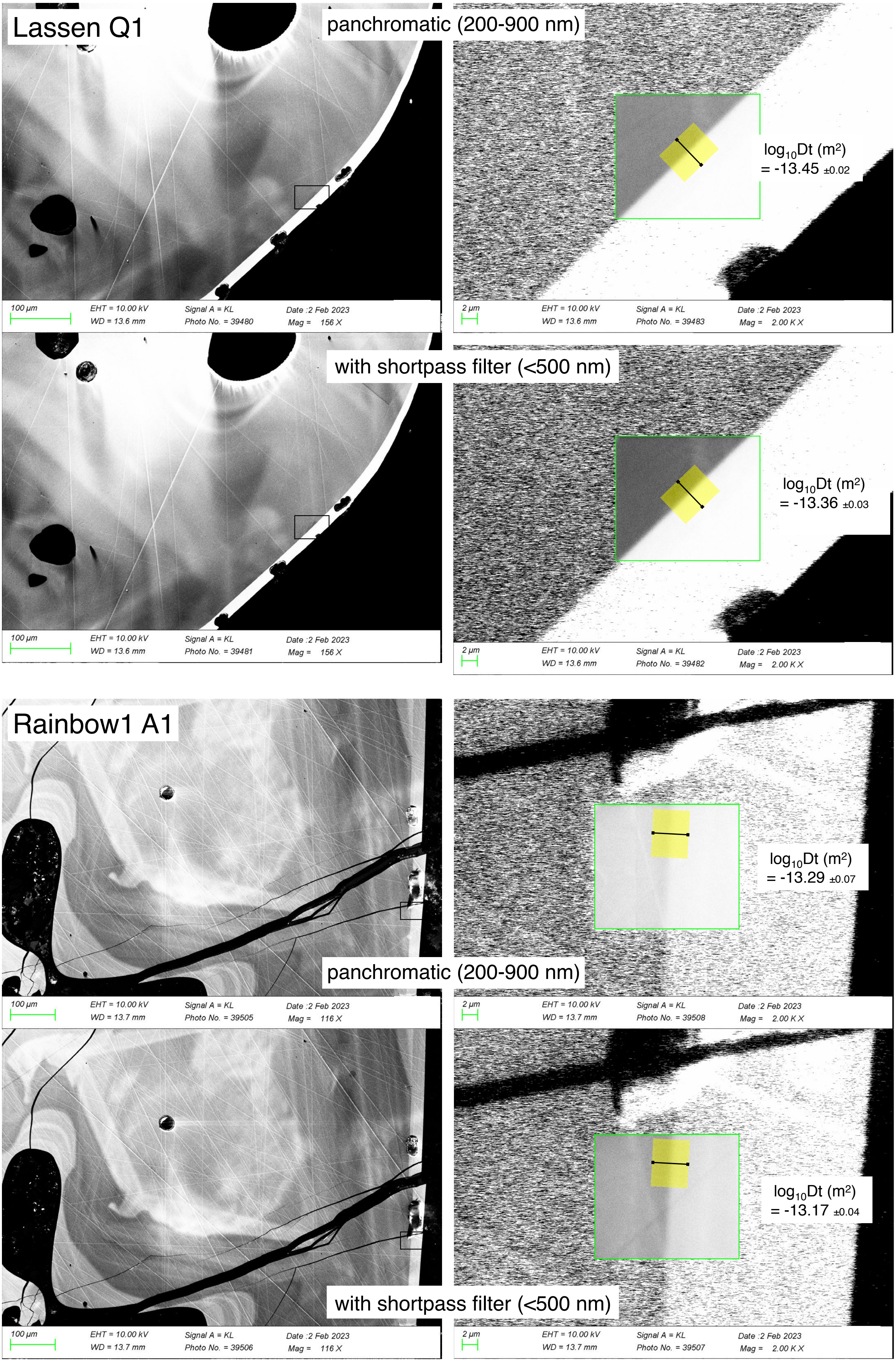
Lassen Q1
panchromatic (200-900 nm)
100 µm
EHT = 10.00 kV
WD = 13.6 mm
Signal A = KL
Photo No. = 39480
Date :2 Feb 2023
Mag = 156 X
log10Dt (m2)
= -13.45 ±0.02
2 µm
EHT = 10.00 kV
WD = 13.6 mm
Signal A = KL
Photo No. = 39483
Date :2 Feb 2023
Mag = 2.00 K X
with shortpass filter (<500 nm)
100 µm
EHT = 10.00 kV
WD = 13.6 mm
Signal A = KL
Photo No. = 39481
Date :2 Feb 2023
Mag = 156 X
log10Dt (m2)
= -13.36 ±0.03
2 µm
EHT = 10.00 kV
WD = 13.6 mm
Signal A = KL
Photo No. = 39482
Date :2 Feb 2023
Mag = 2.00 K X
Rainbow1 A1
log10Dt (m2)
= -13.29 ±0.07
panchromatic (200-900 nm)
100 µm
EHT = 10.00 kV
WD = 13.7 mm
Signal A = KL
Photo No. = 39505
Date :2 Feb 2023
Mag = 116 X
2 µm
EHT = 10.00 kV
WD = 13.7 mm
Signal A = KL
Photo No. = 39508
Date :2 Feb 2023
Mag = 2.00 K X
log10Dt (m2)
= -13.17 ±0.04
with shortpass filter (<500 nm)
100 µm
EHT = 10.00 kV
WD = 13.7 mm
Signal A = KL
Photo No. = 39506
Date :2 Feb 2023
Mag = 116 X
2 µm
EHT = 10.00 kV
WD = 13.7 mm
Signal A = KL
Photo No. = 39507
Date :2 Feb 2023
Mag = 2.00 K X

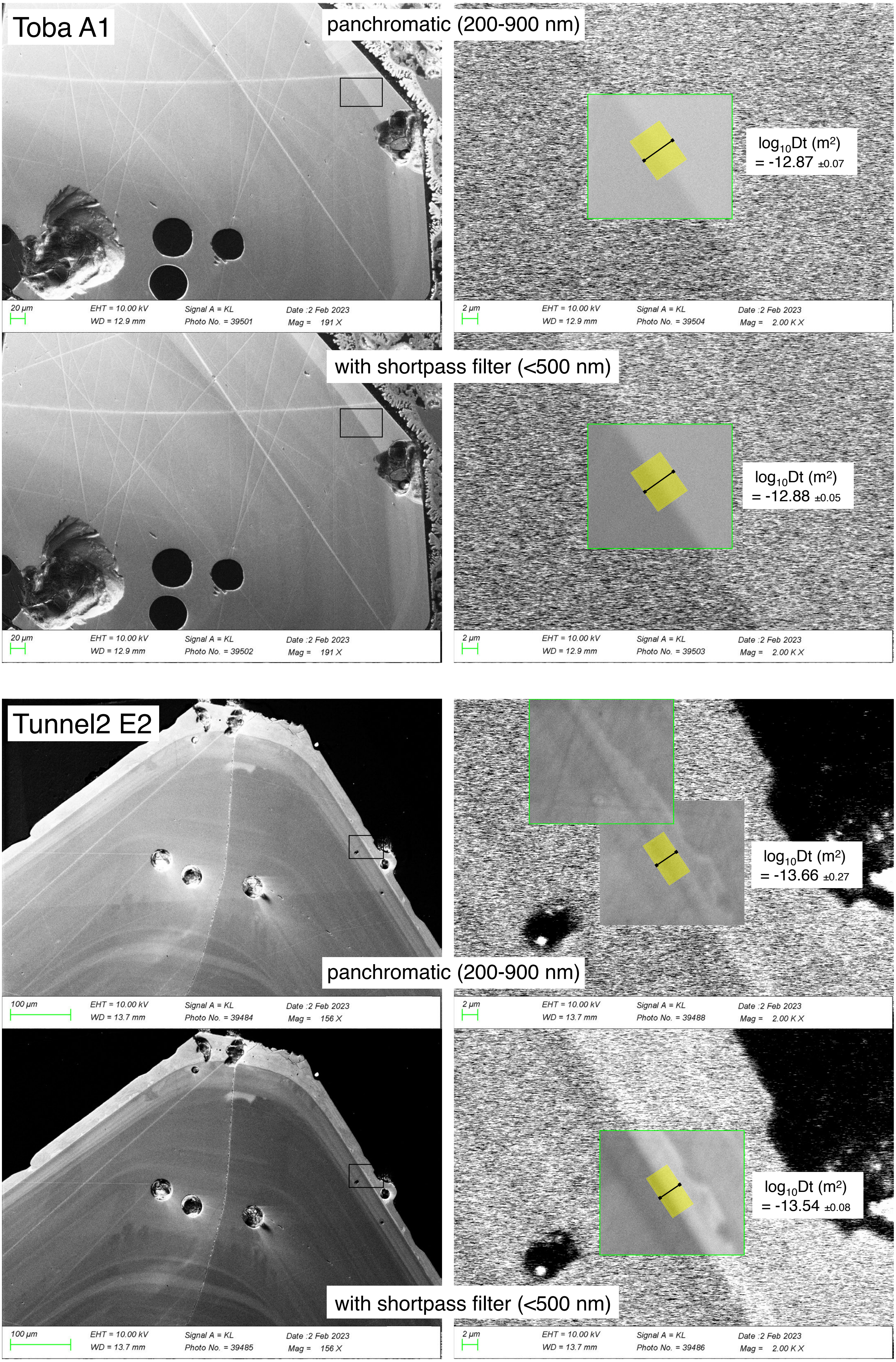
Toba A1
panchromatic (200-900 nm)
log10Dt (m2)
= -12.87 ±0.07
20 µm
EHT = 10.00 kV
WD = 12.9 mm
Signal A = KL
Photo No. = 39501
Date :2 Feb 2023
Mag = 191 X
2 µm
EHT = 10.00 kV
WD = 12.9 mm
Signal A = KL
Photo No. = 39504
Date :2 Feb 2023
Mag = 2.00 K X
with shortpass filter (<500 nm)
log10Dt (m2)
= -12.88 ±0.05
20 µm
EHT = 10.00 kV
WD = 12.9 mm
Signal A = KL
Photo No. = 39502
Date :2 Feb 2023
Mag = 191 X
2 µm
EHT = 10.00 kV
WD = 12.9 mm
Signal A = KL
Photo No. = 39503
Date :2 Feb 2023
Mag = 2.00 K X
Tunnel2 E2
log10Dt (m2)
= -13.66 ±0.27
panchromatic (200-900 nm)
100 µm
EHT = 10.00 kV
WD = 13.7 mm
Signal A = KL
Photo No. = 39484
Date :2 Feb 2023
Mag = 156 X
2 µm
EHT = 10.00 kV
WD = 13.7 mm
Signal A = KL
Photo No. = 39488
Date :2 Feb 2023
Mag = 2.00 K X
log10Dt (m2)
= -13.54 ±0.08
with shortpass filter (<500 nm)
100 µm
EHT = 10.00 kV
WD = 13.7 mm
Signal A = KL
Photo No. = 39485
Date :2 Feb 2023
Mag = 156 X
2 µm
EHT = 10.00 kV
WD = 13.7 mm
Signal A = KL
Photo No. = 39486
Date :2 Feb 2023
Mag = 2.00 K X
