# Supplementary material for: New constraints on Ti diffusion in quartz and the priming of silicic volcanic eruptions
Source: Nat Commun. 2023 Jul 17;14:4277. doi: 10.1038/s41467-023-39912-5 (PMC10352339; doi:10.1038/s41467-023-39912-5)
Supplement: Supplementary file 5 — Supplementary Data 2 [file 41467_2023_39912_MOESM5_ESM.pdf]

8Aa-8

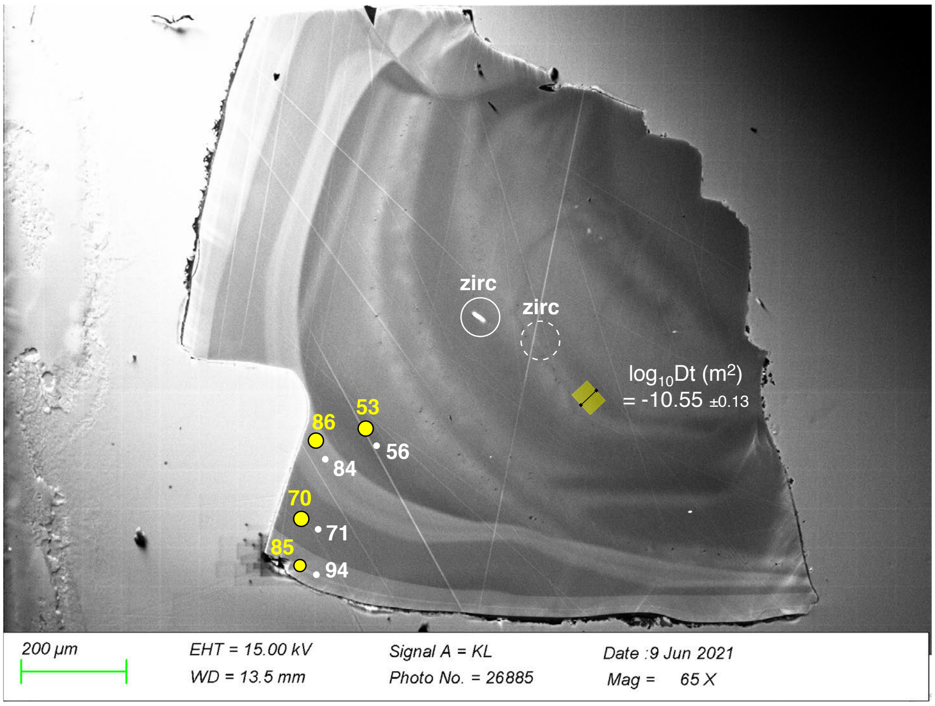

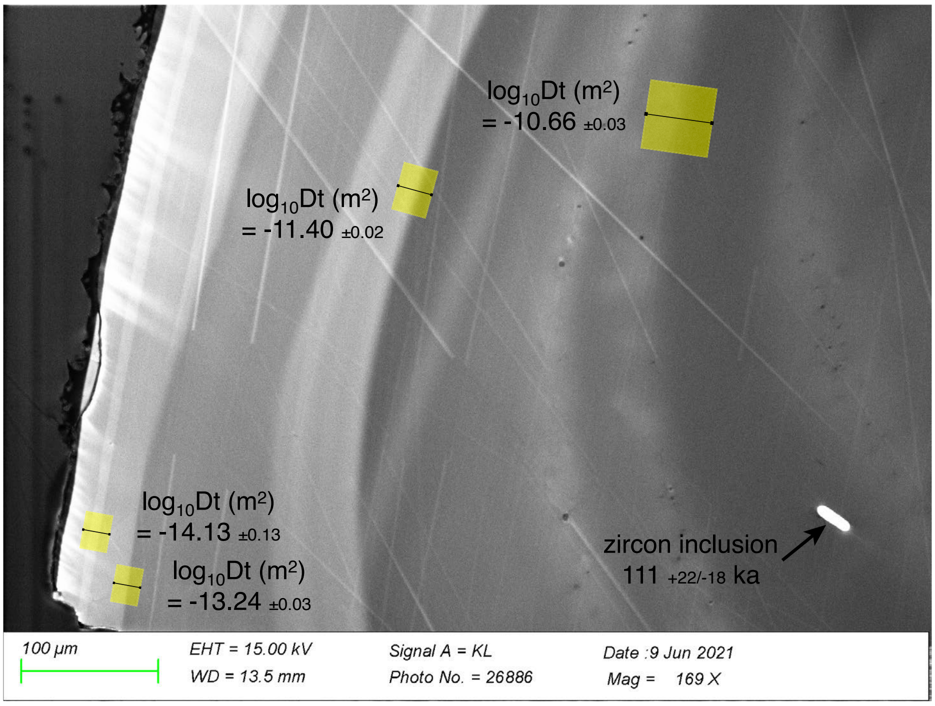

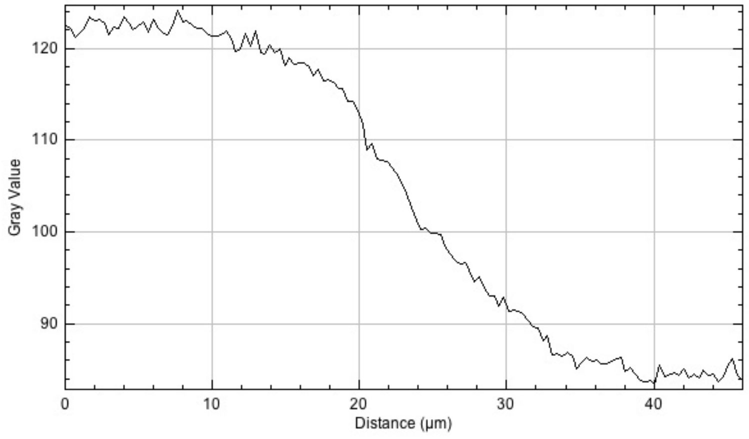

Grayscale profile from ImageJ

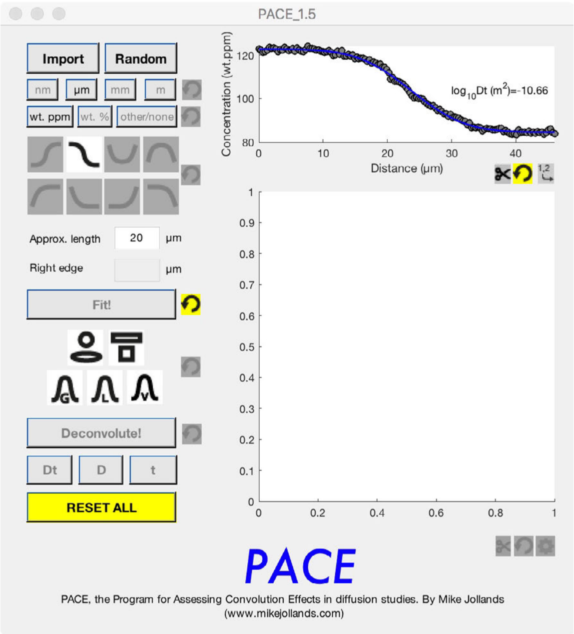

Profile fit in PACE

8Aa-15

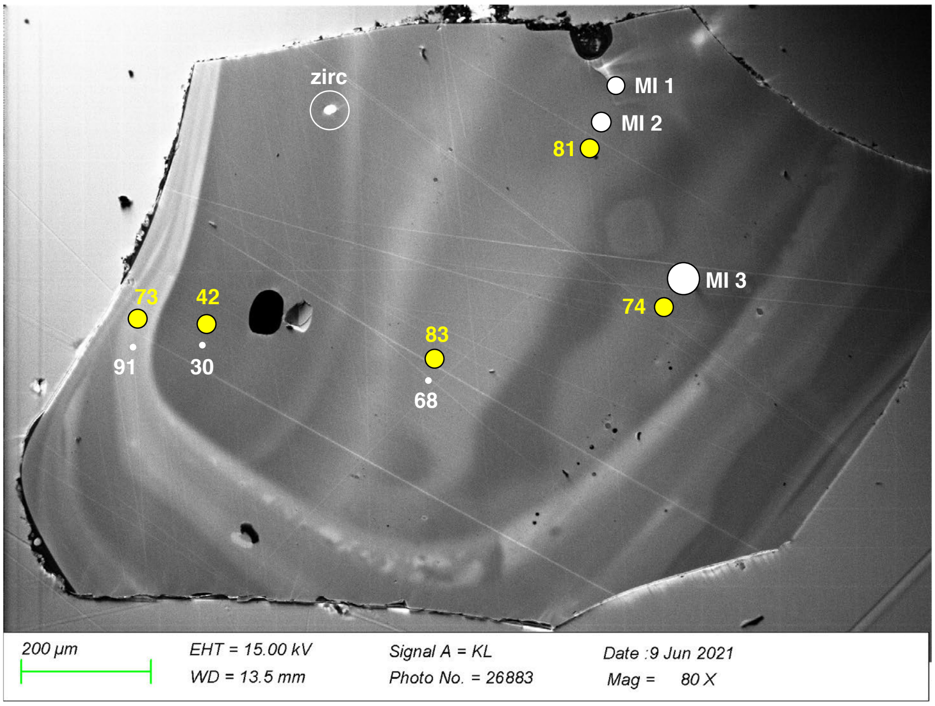

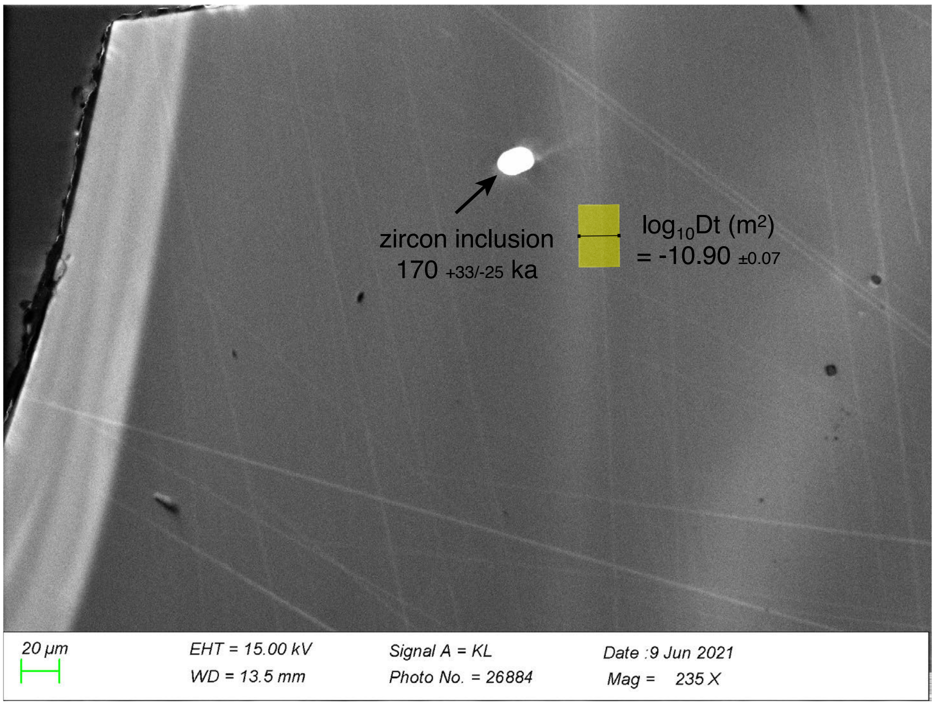

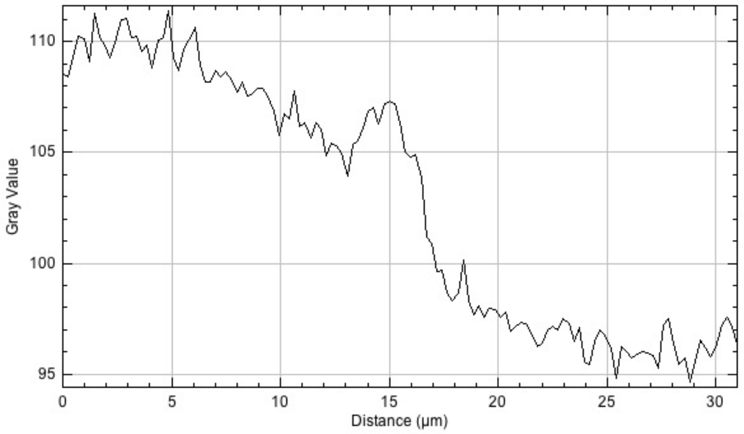

Grayscale profile from ImageJ (the hump at 15 μm stems from a scratch and was removed prior to the profile fitting)

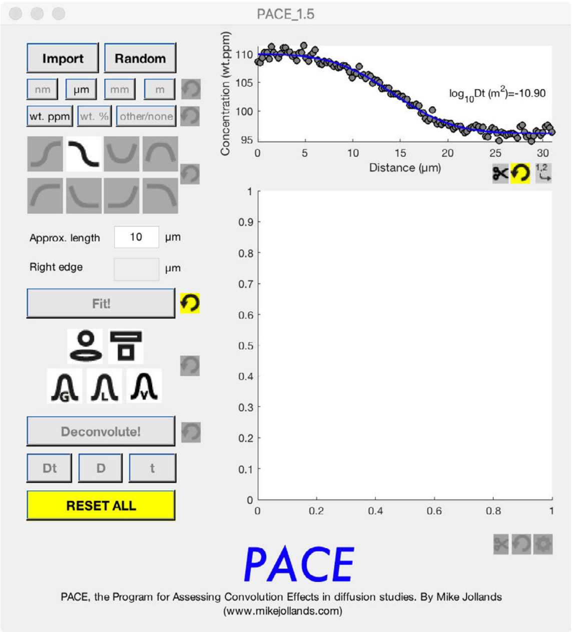

Profile fit in PACE

8Ab-3

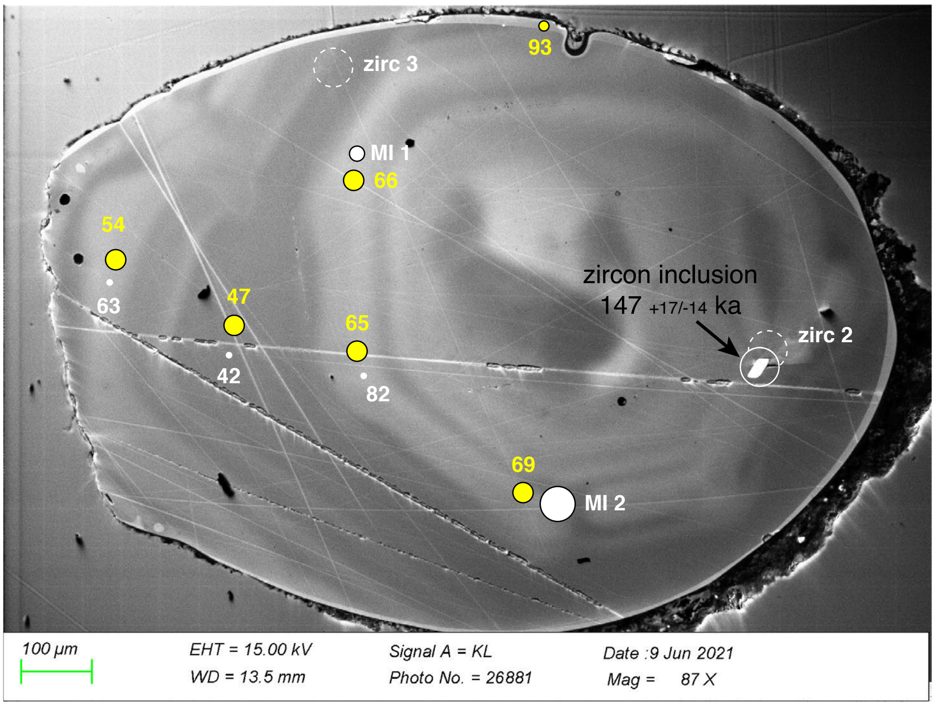

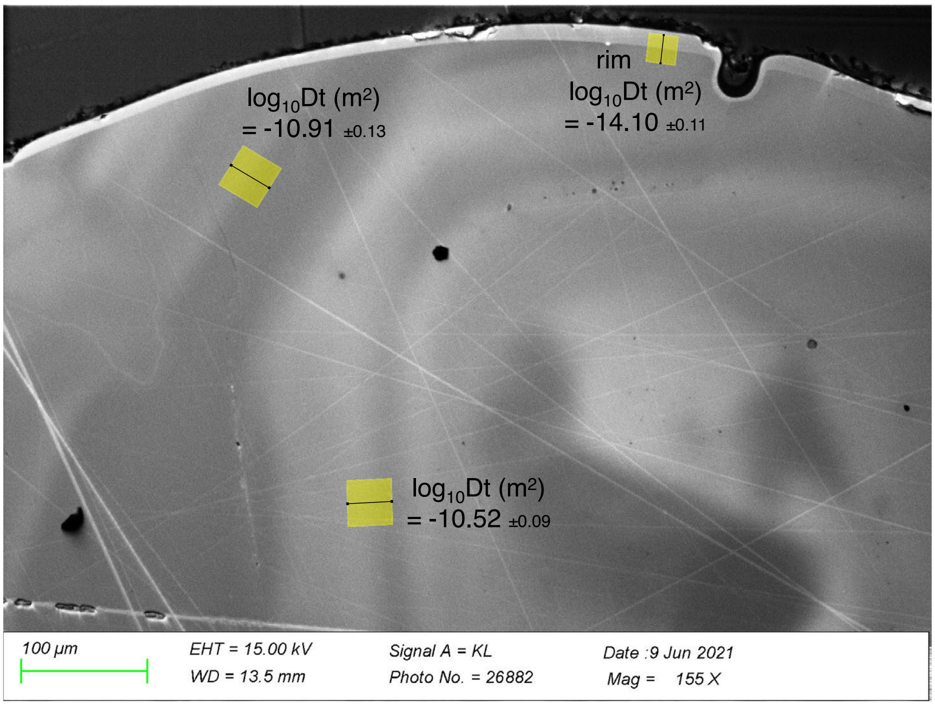

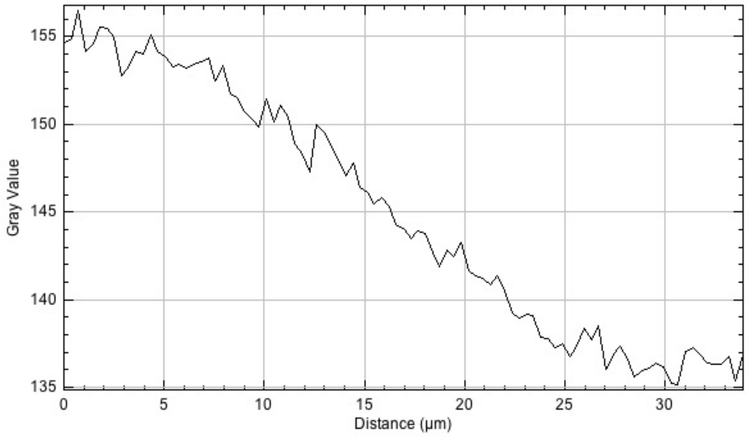

Grayscale profile from ImageJ

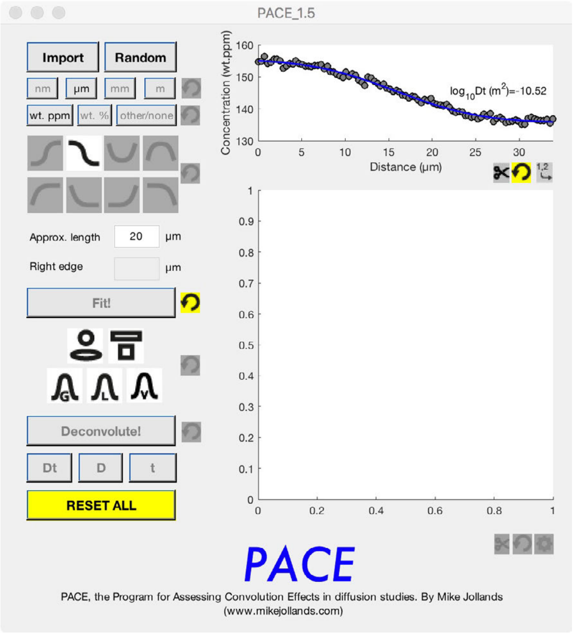

Profile fit in PACE

8B-11

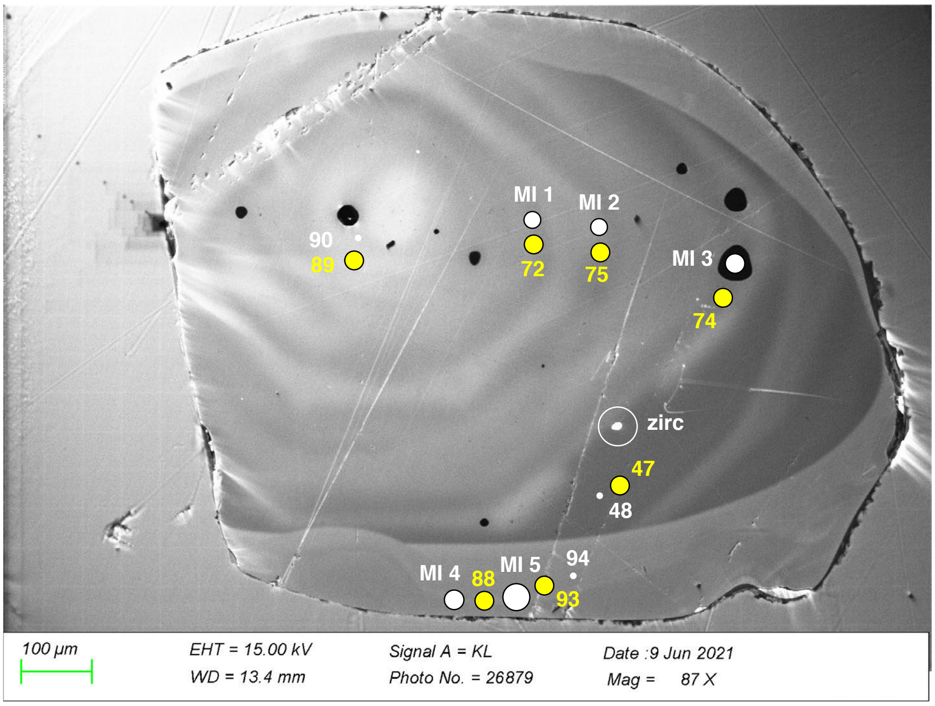

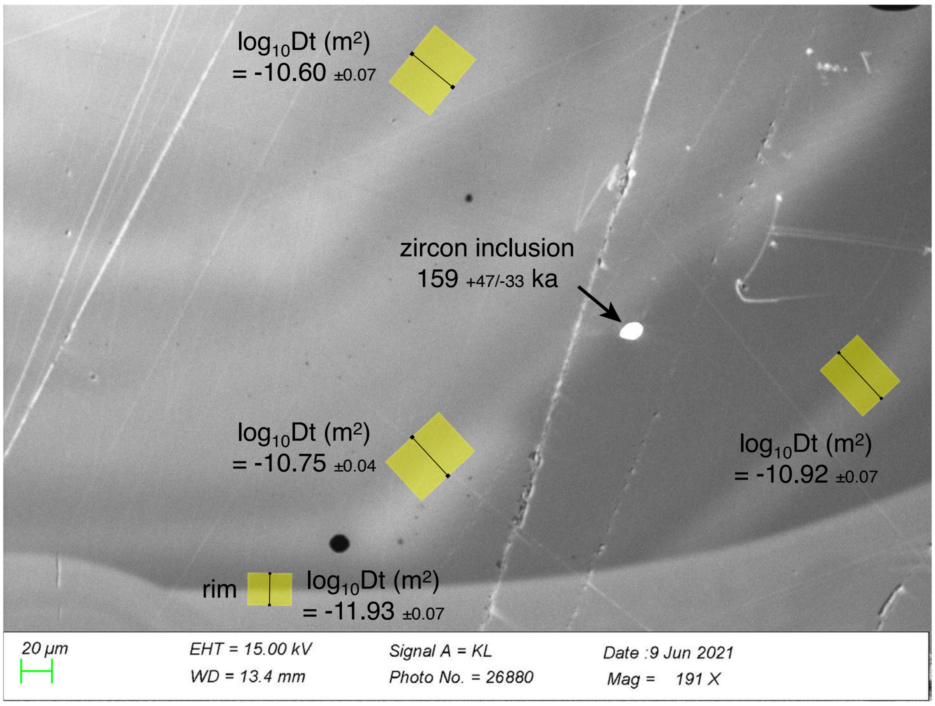

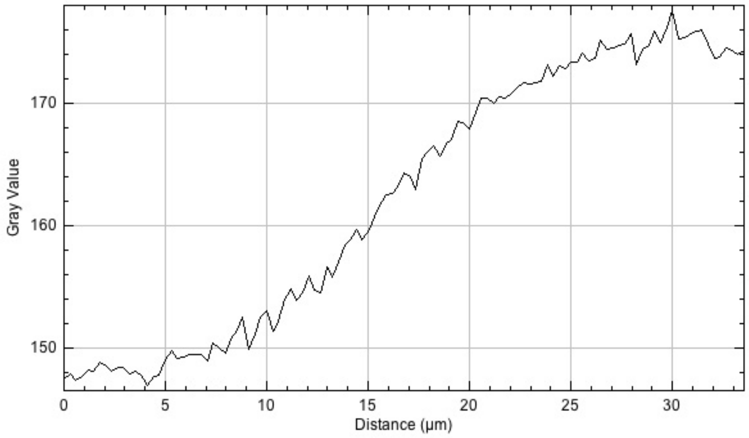

Grayscale profile from ImageJ

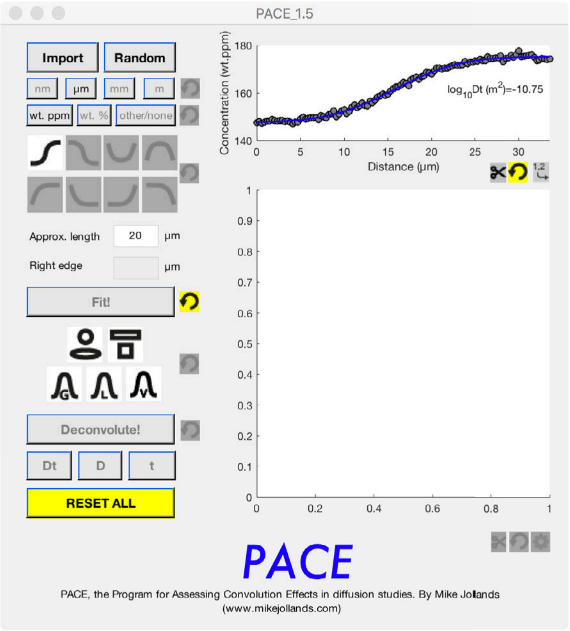

Profile fit in PACE

8Da-1

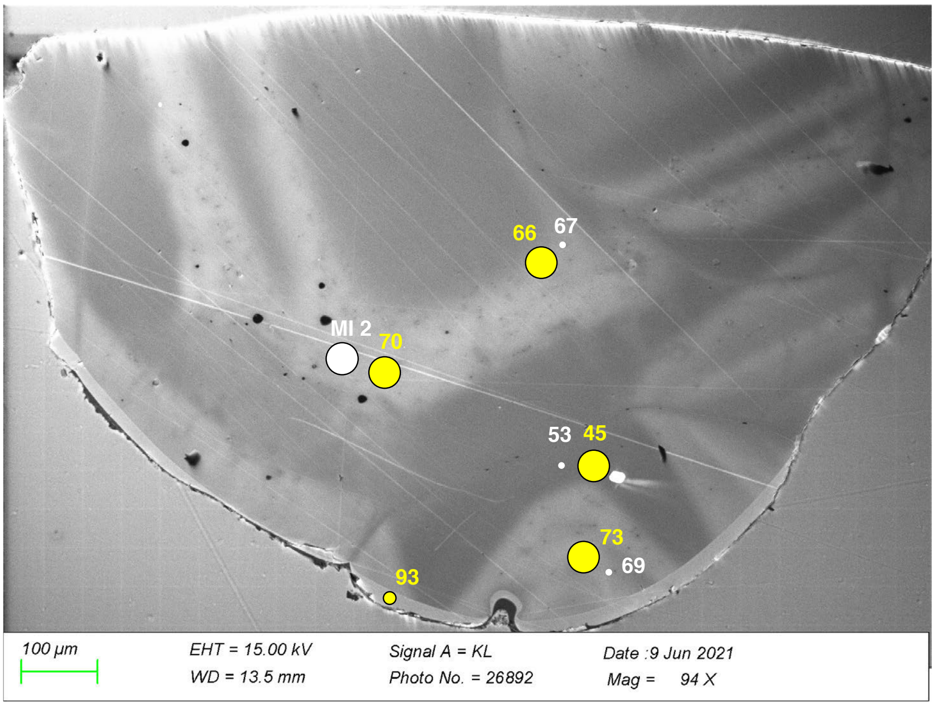

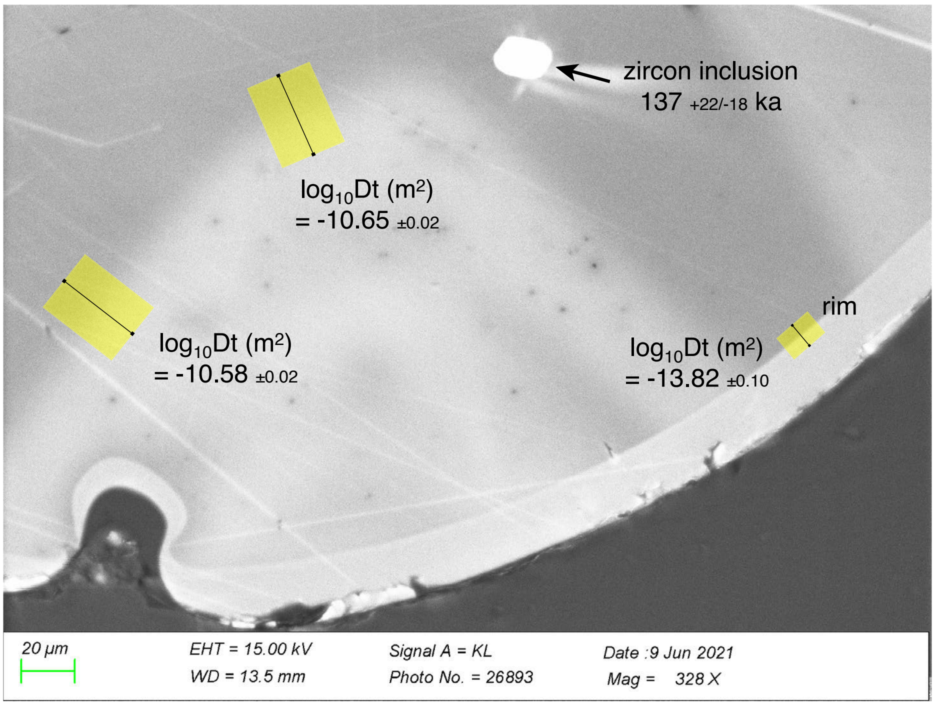

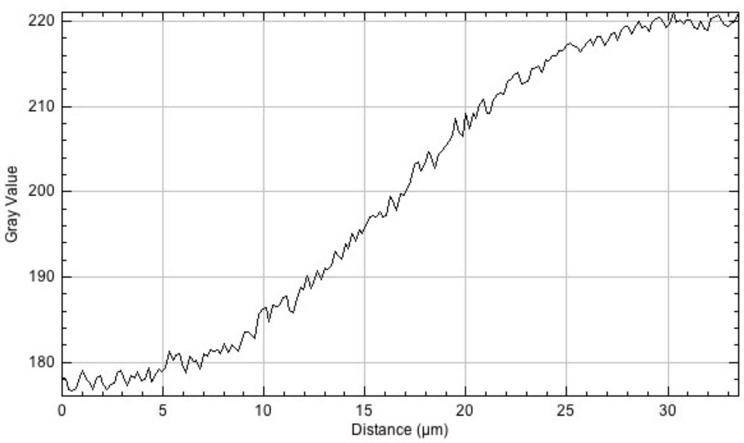

Grayscale profile from ImageJ

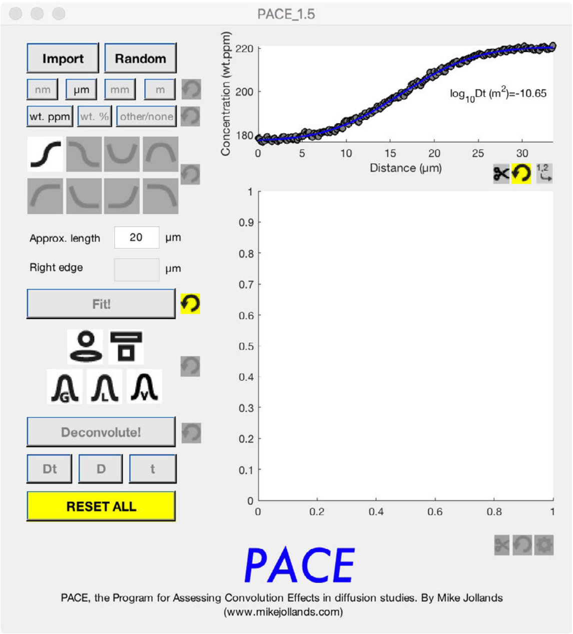

Profile fit in PACE

8Da-9

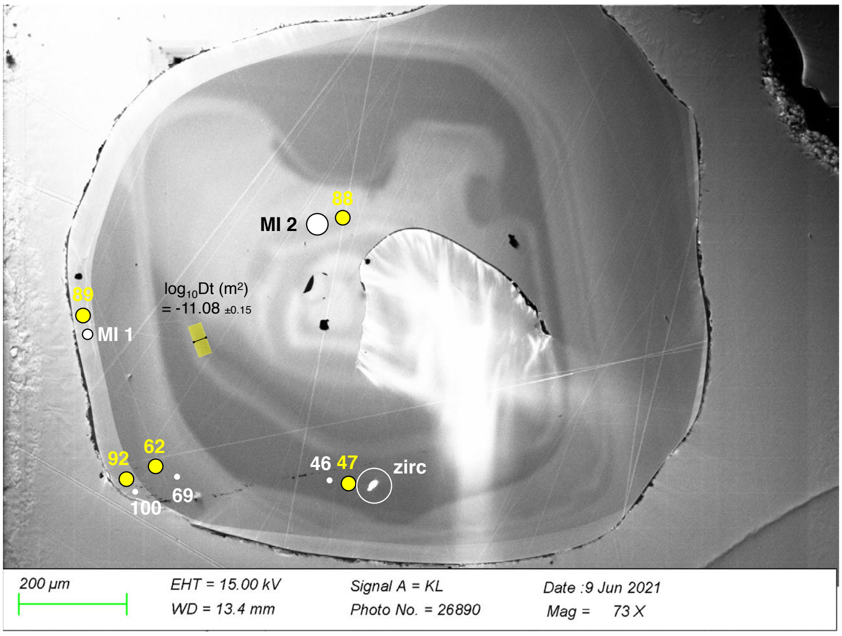

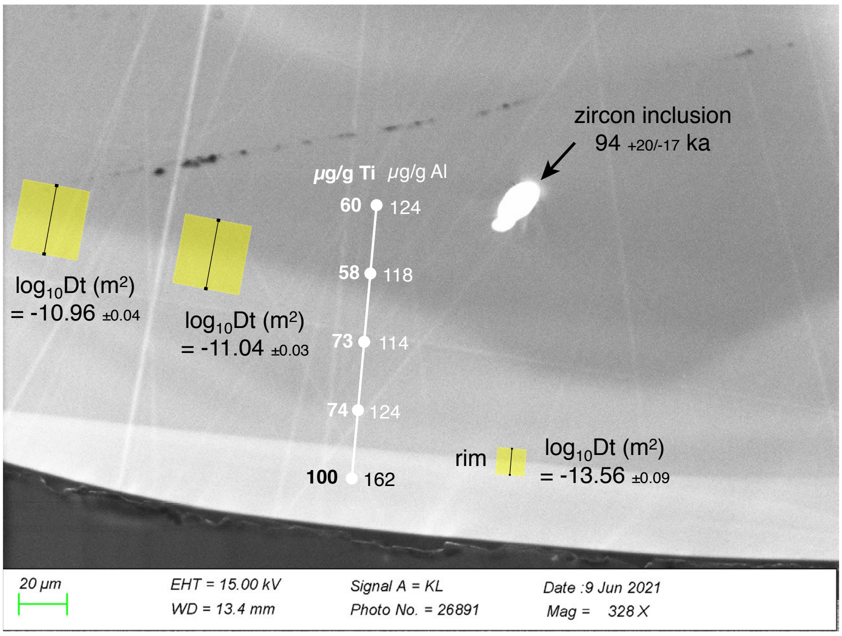

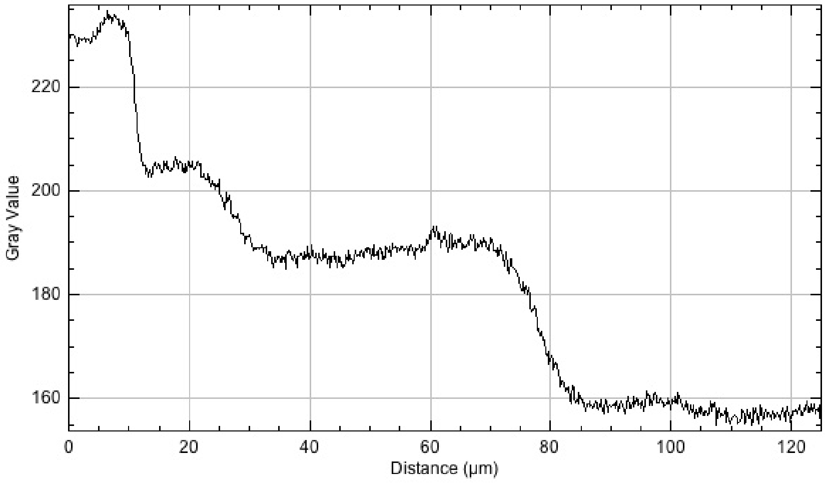

ImageJ grayscale profile along EPMA profile

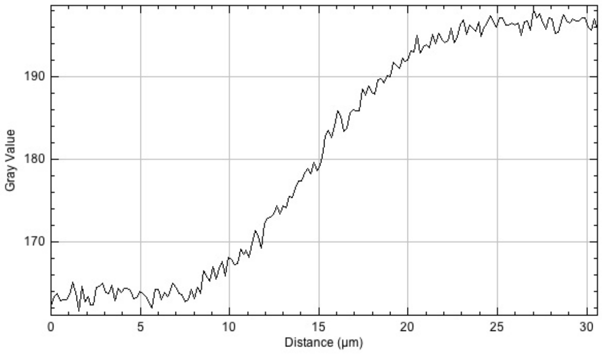

Grayscale profile from ImageJ

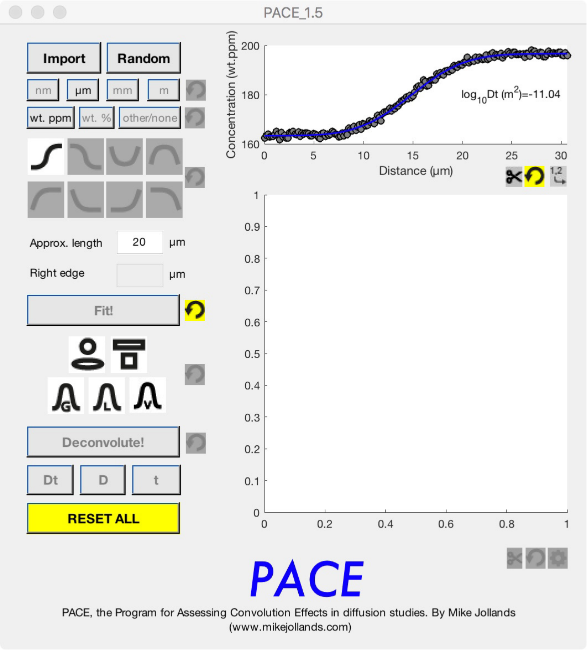

Profile fit in PACE

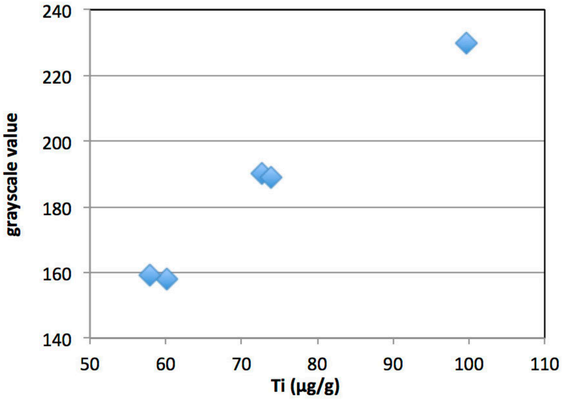

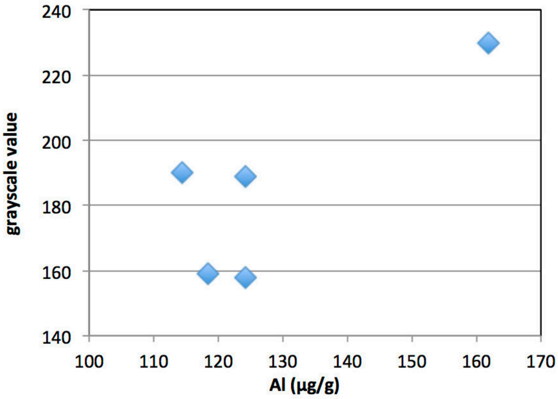

Correspondence between grayscale value and Ti and Al concentration along EPMA profile

8Da-11

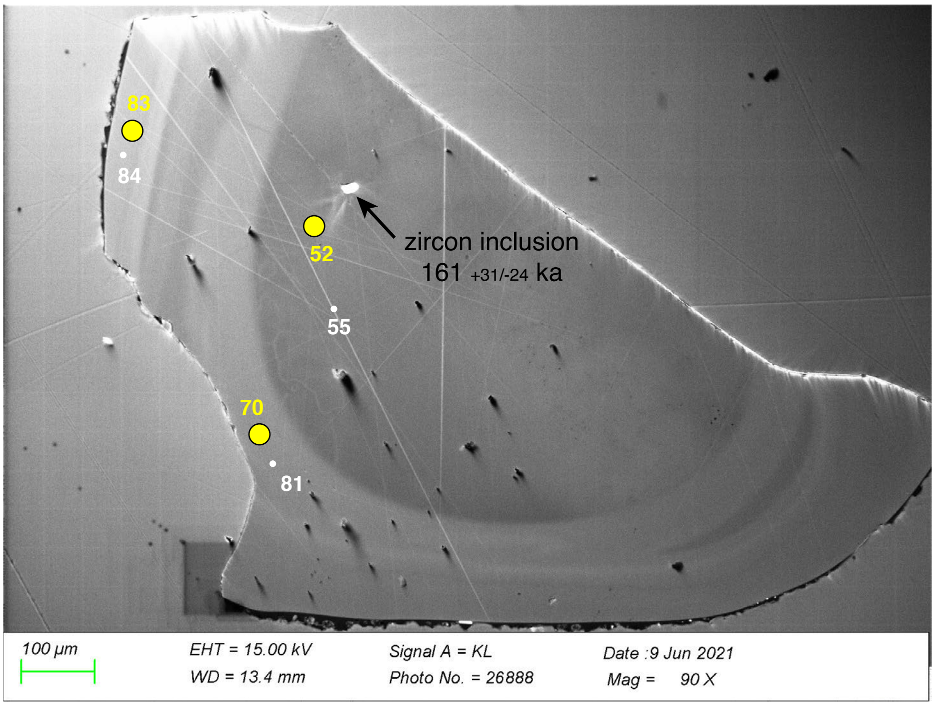

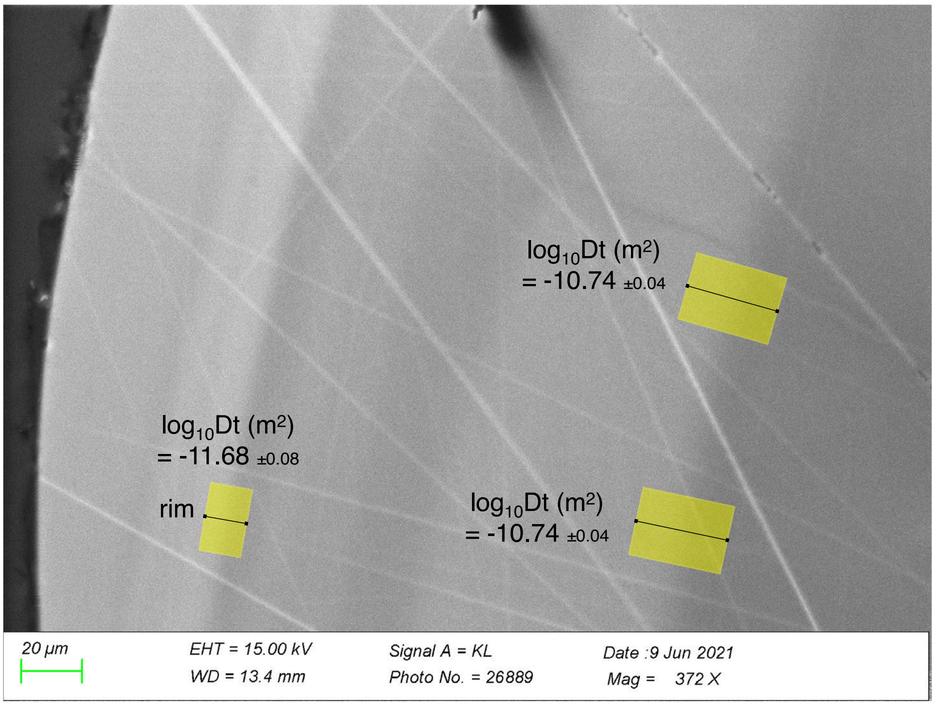

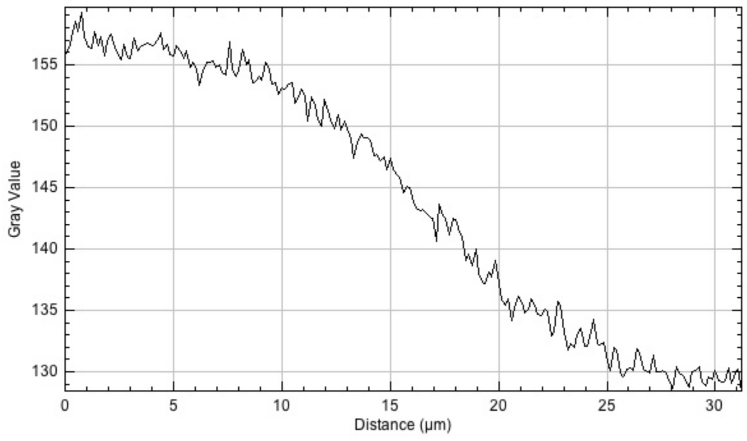

Grayscale profile from ImageJ

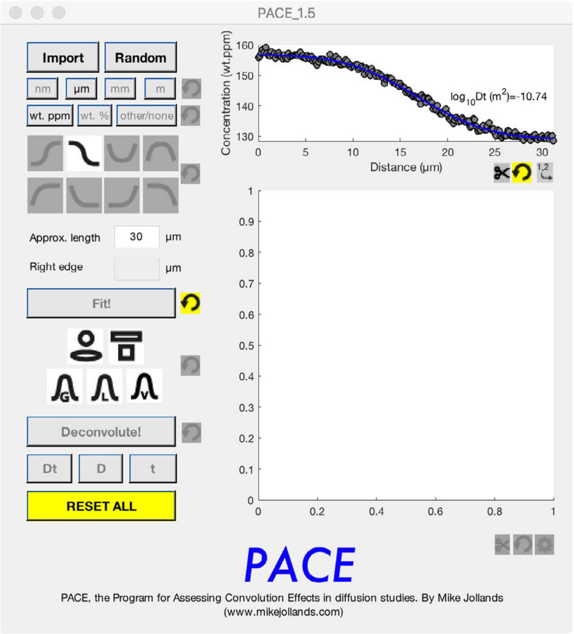

Profile fit in PACE

8Da-15

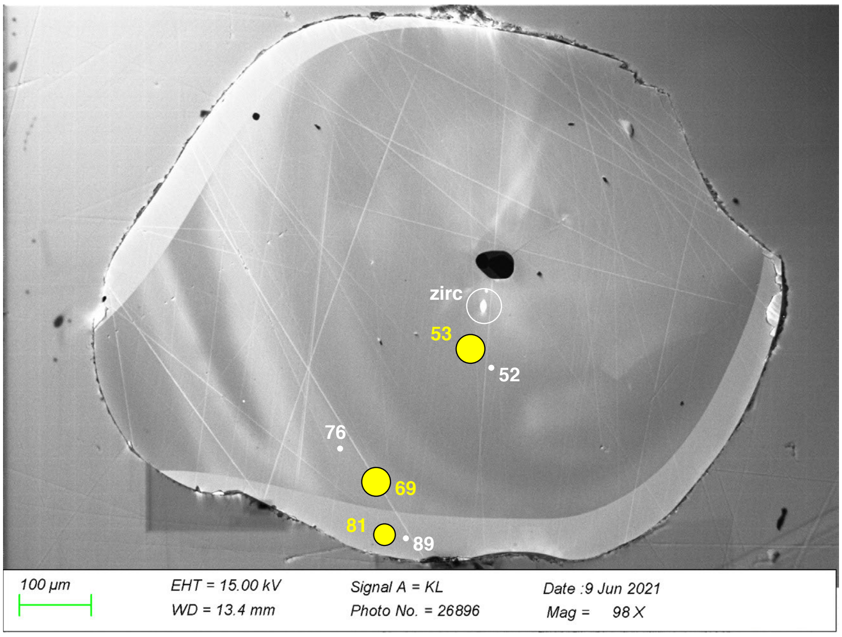

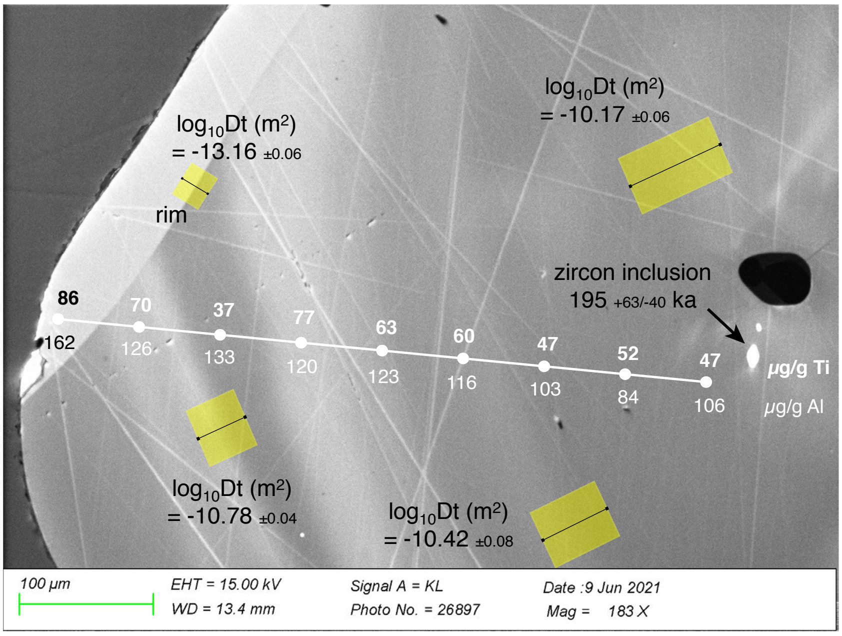

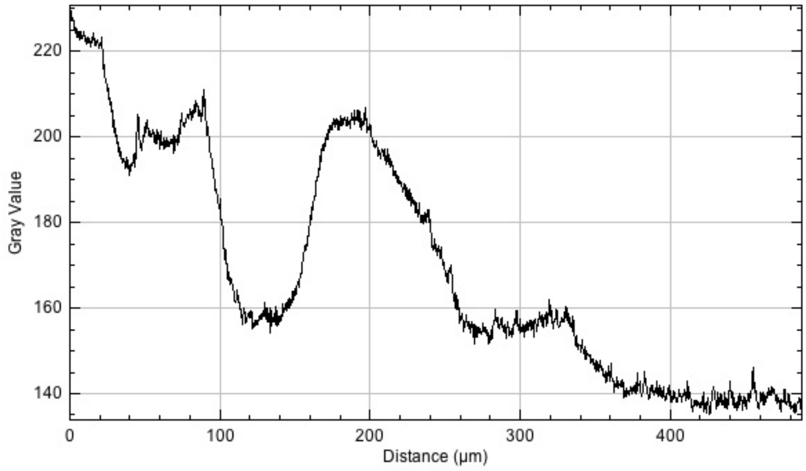

ImageJ grayscale profile along EPMA profile

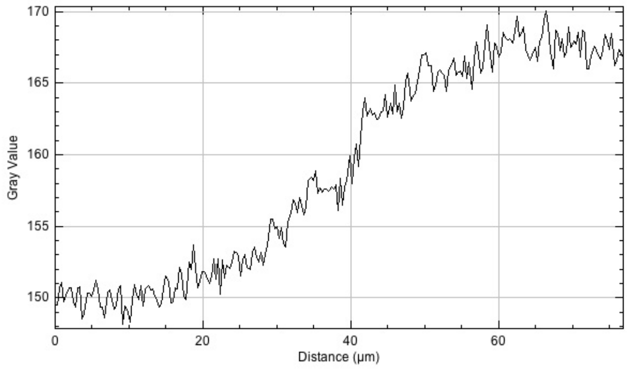

Grayscale profile from ImageJ

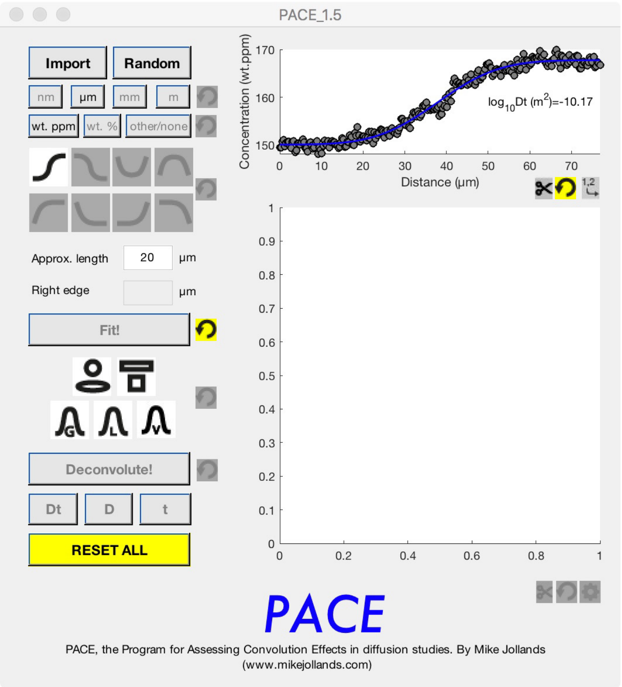

Profile fit in PACE

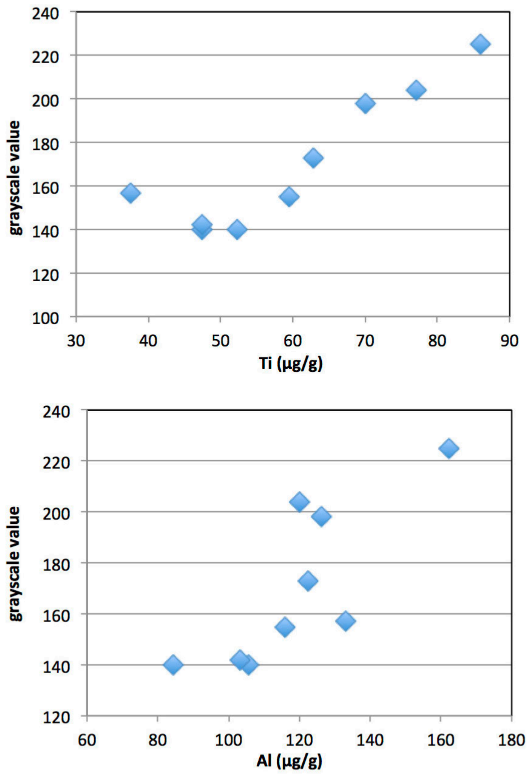

Correspondence between grayscale value and Ti and Al concentration along EPMA profile

8Da-16

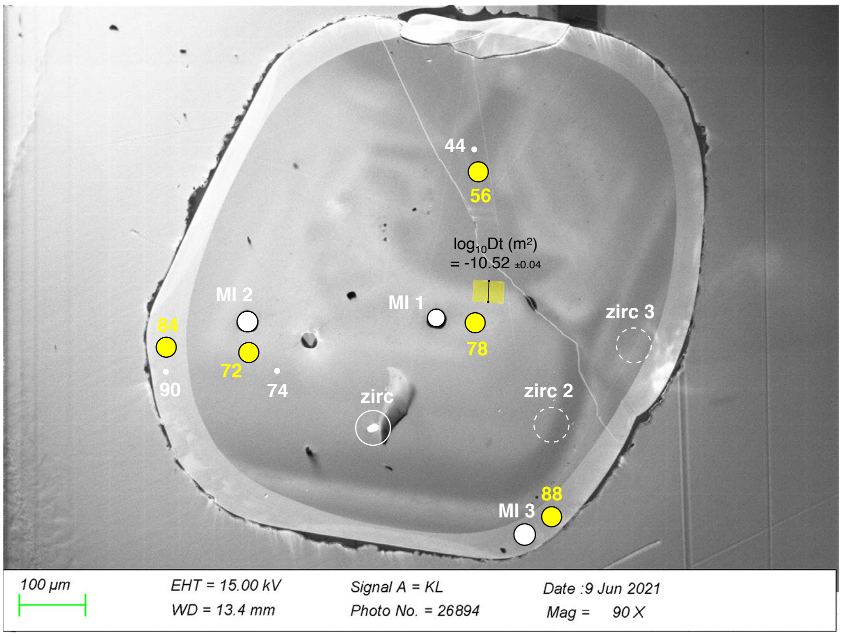

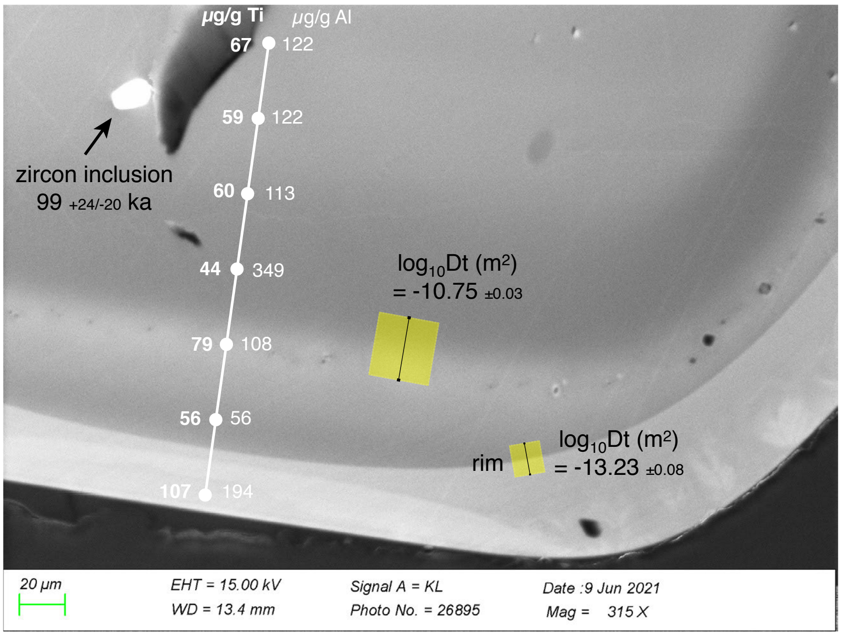

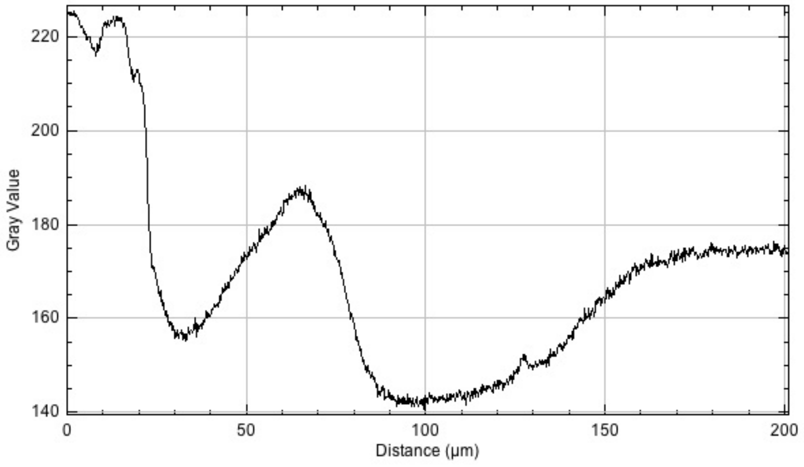

ImageJ grayscale profile along EPMA profile

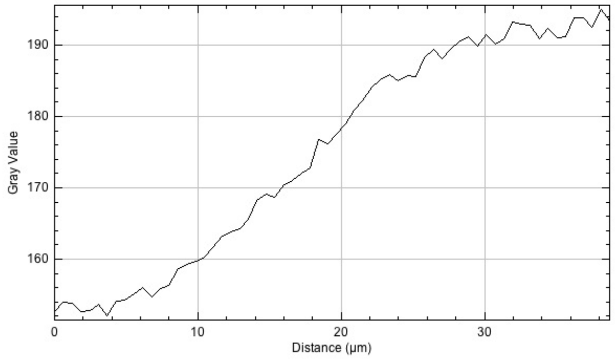

Grayscale profile from ImageJ

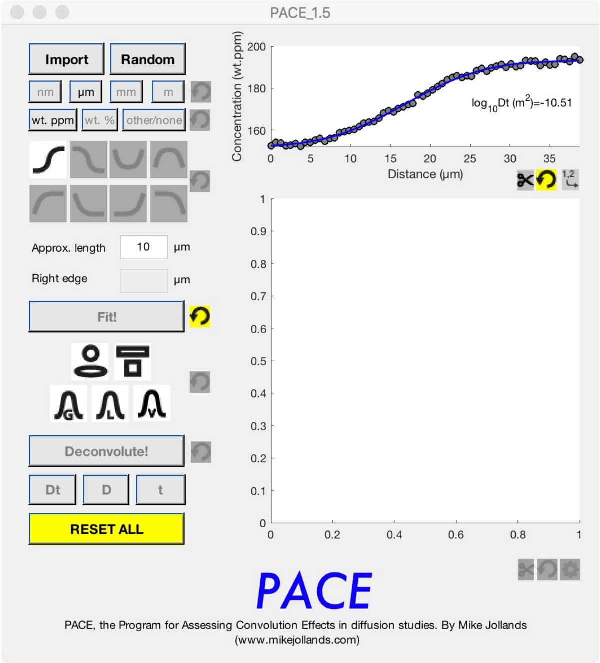

Profile fit in PACE

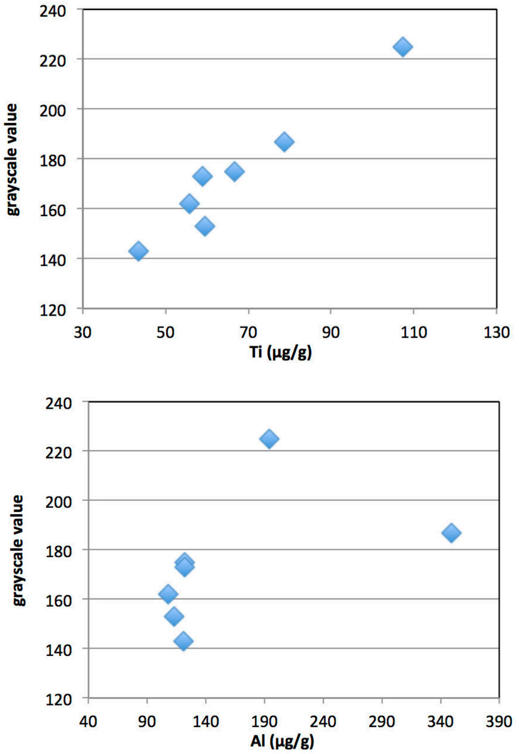

Correspondence between grayscale value and Ti and Al concentration along EPMA profile

8Db-4

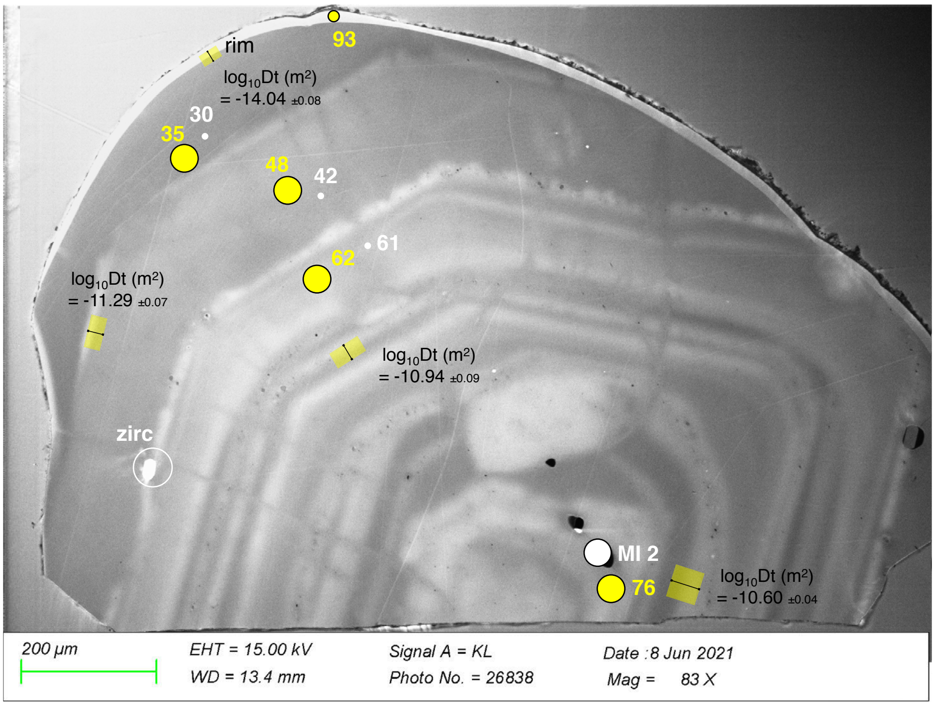

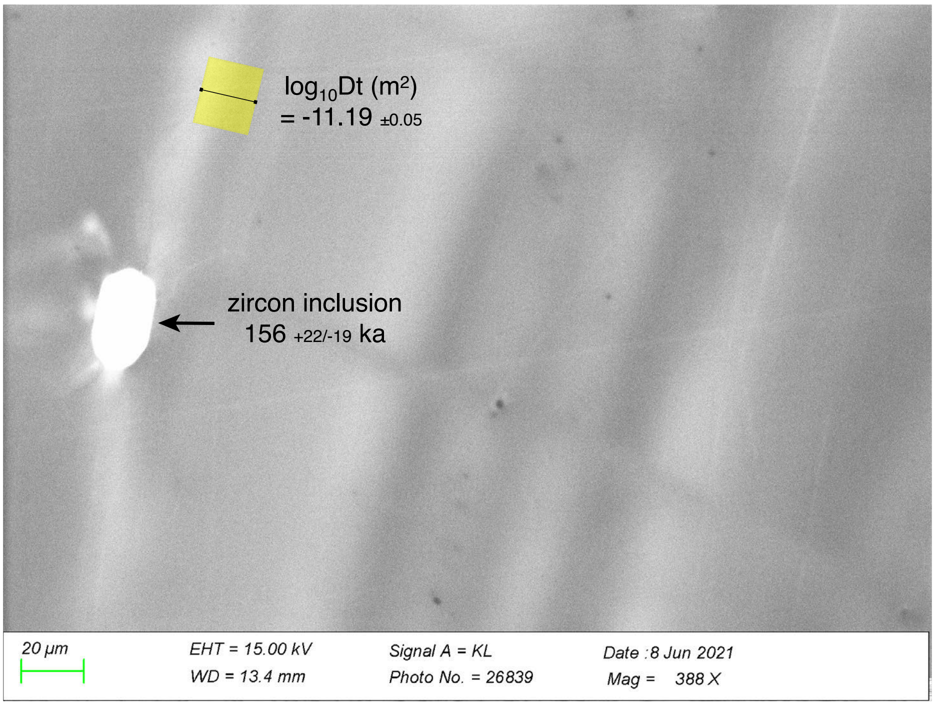

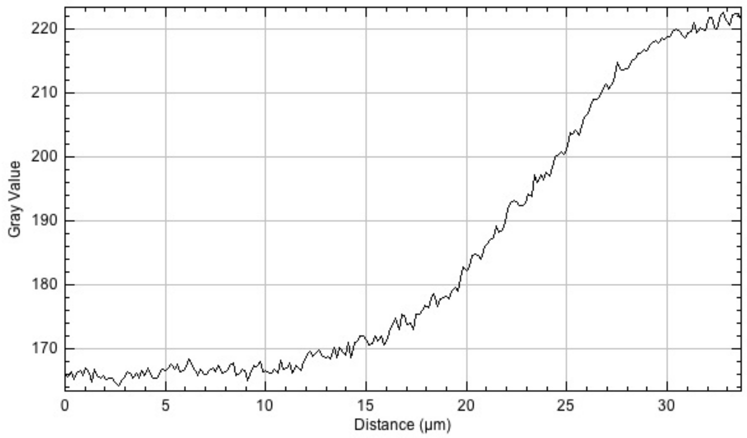

Grayscale profile from ImageJ

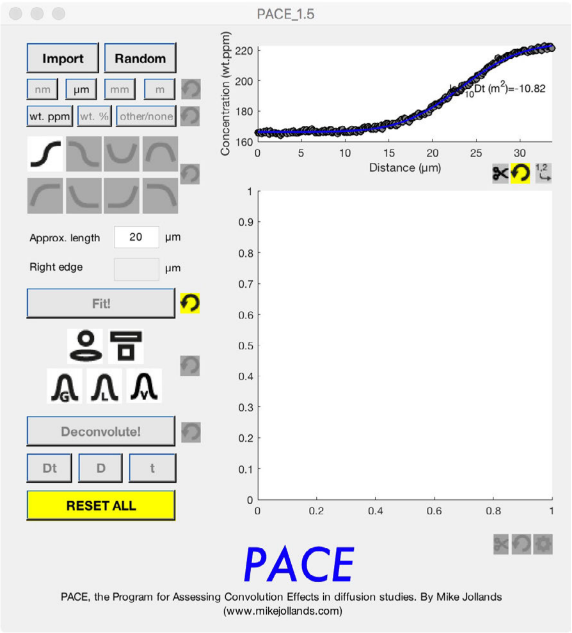

Profile fit in PACE

8Db-5

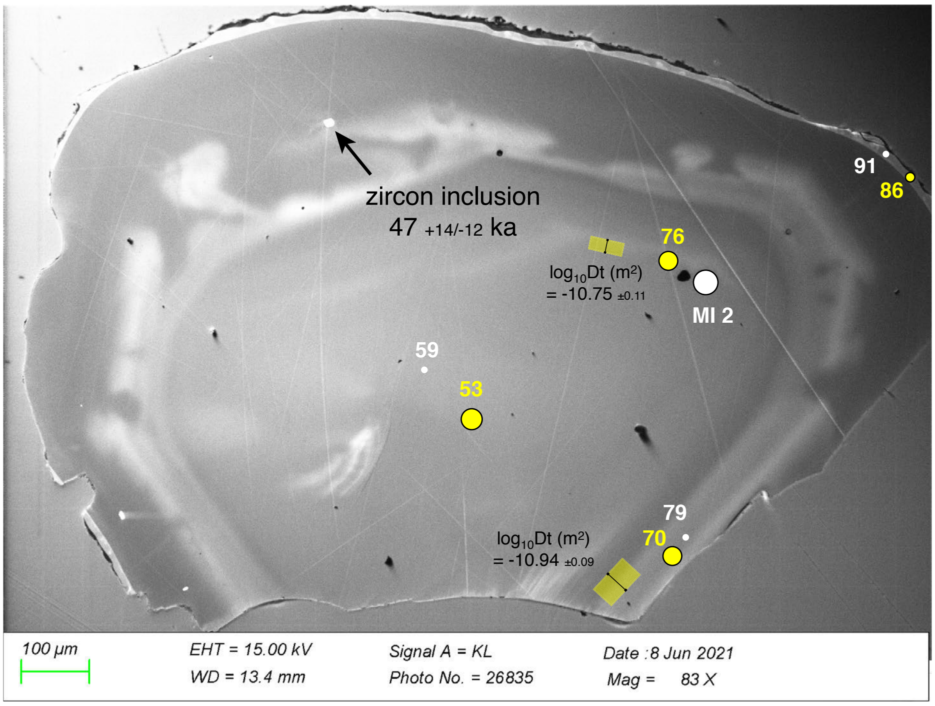

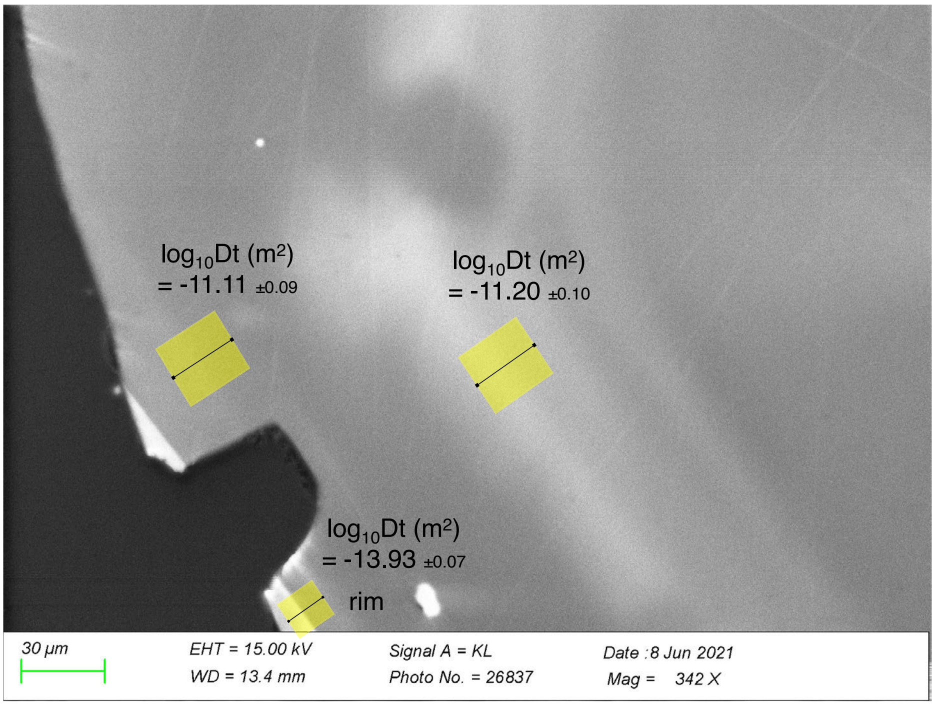

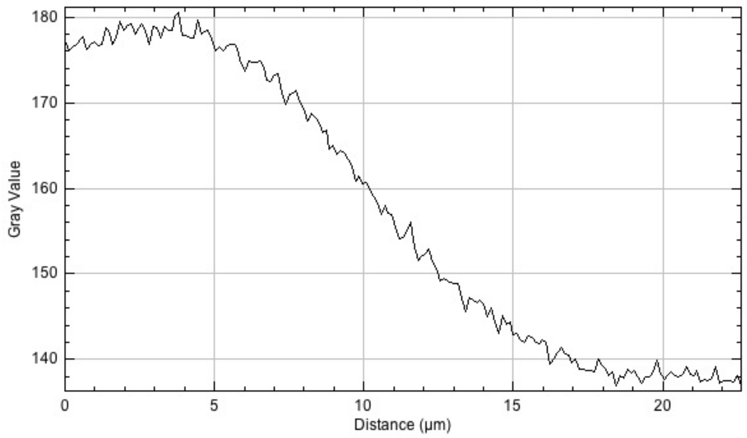

Grayscale profile from ImageJ

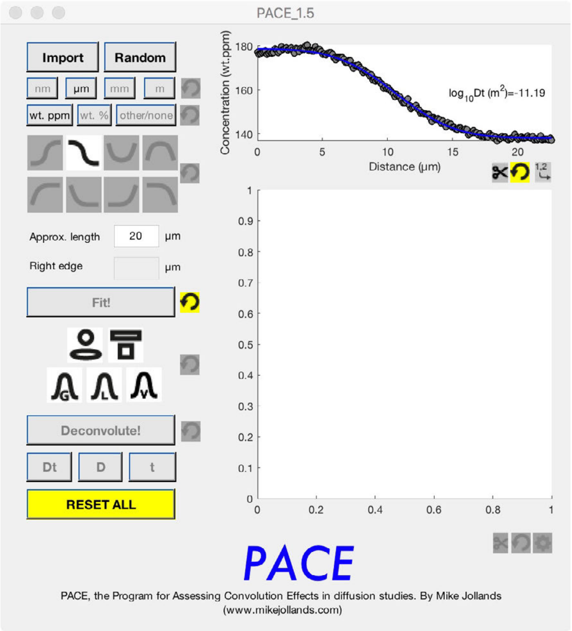

Profile fit in PACE

8Db-9

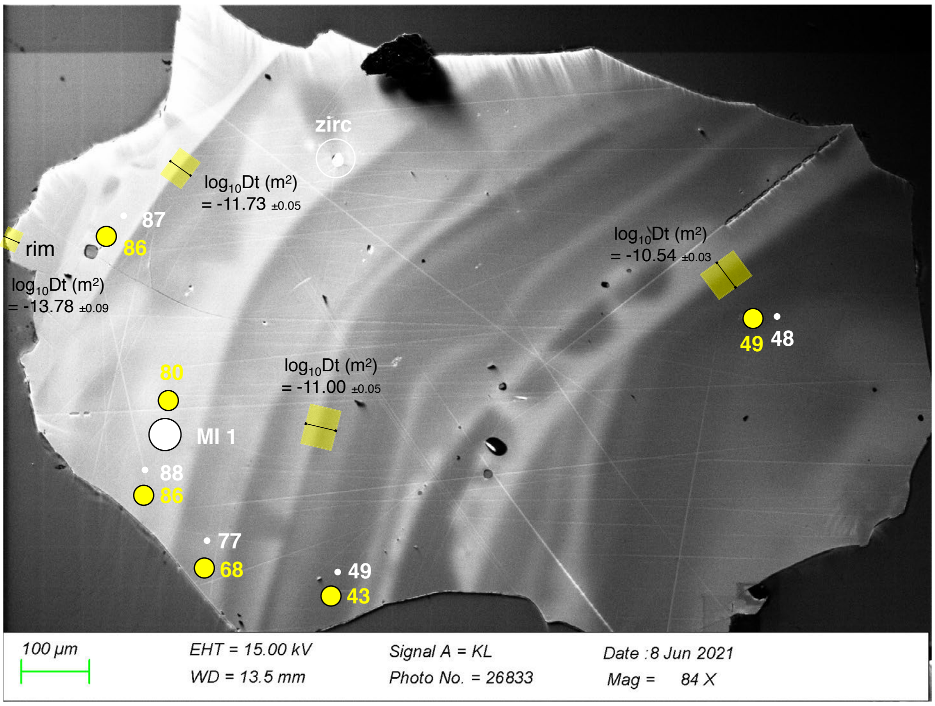

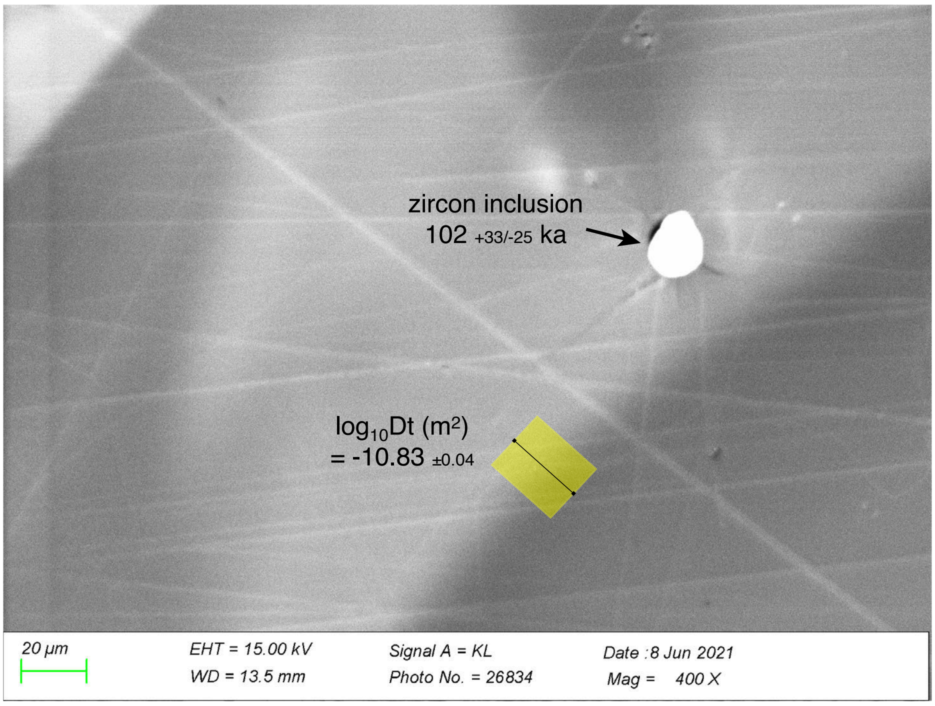

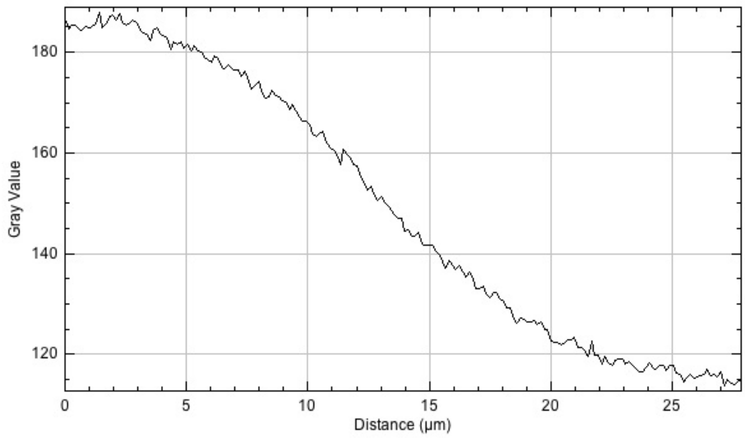

Grayscale profile from ImageJ

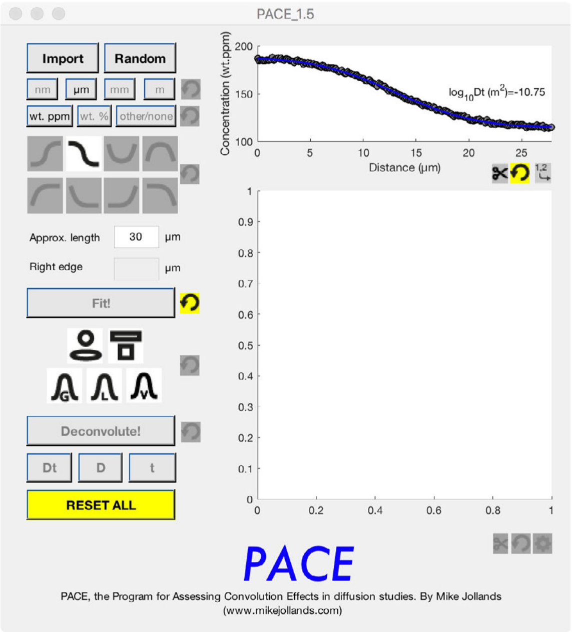

Profile fit in PACE

QtzIIa-8

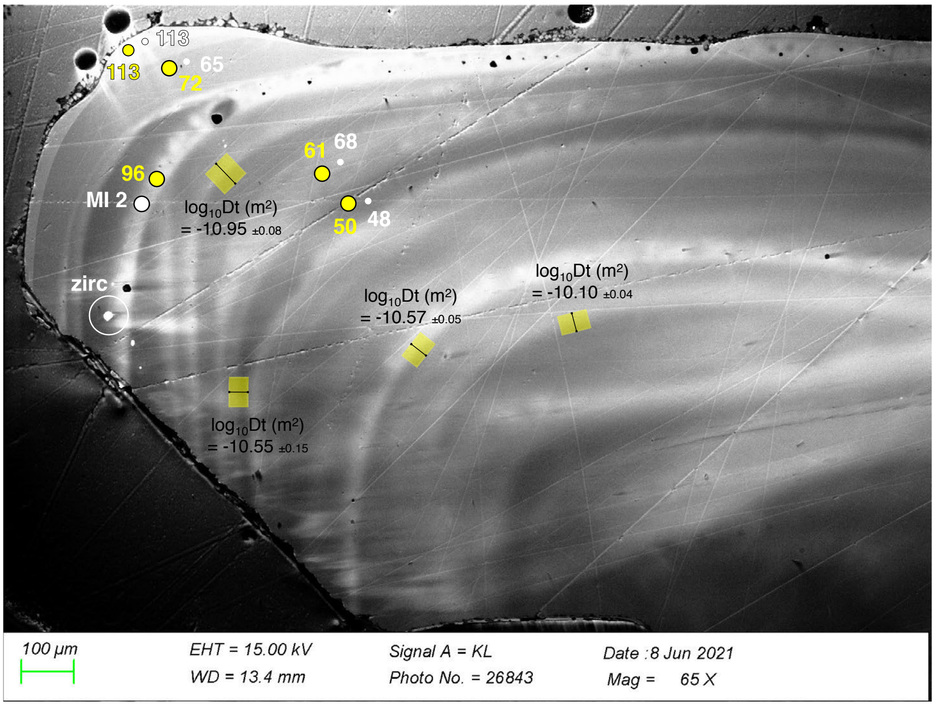

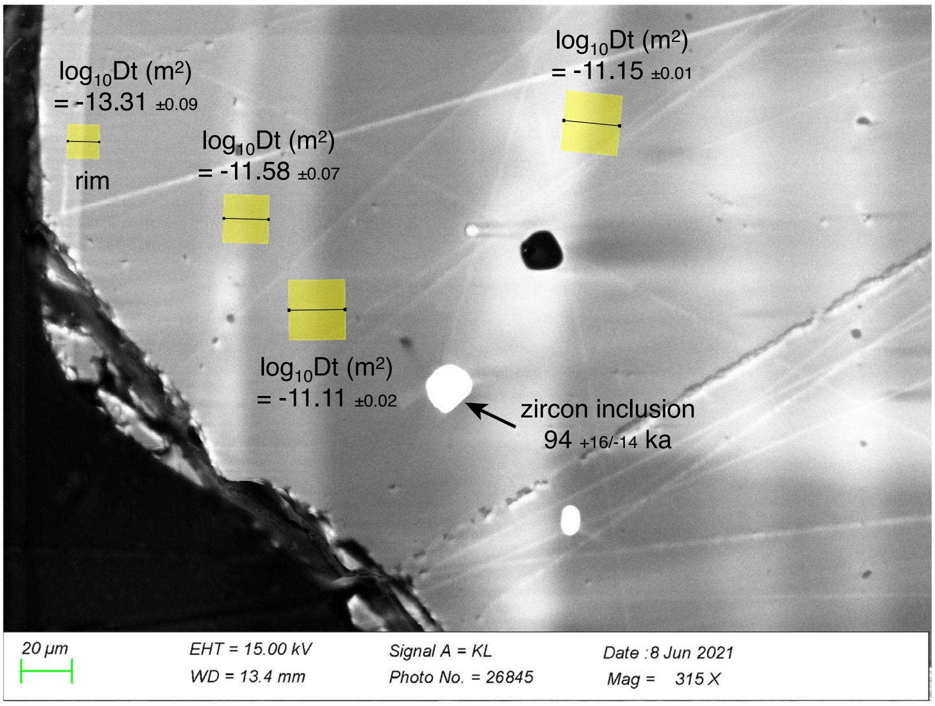

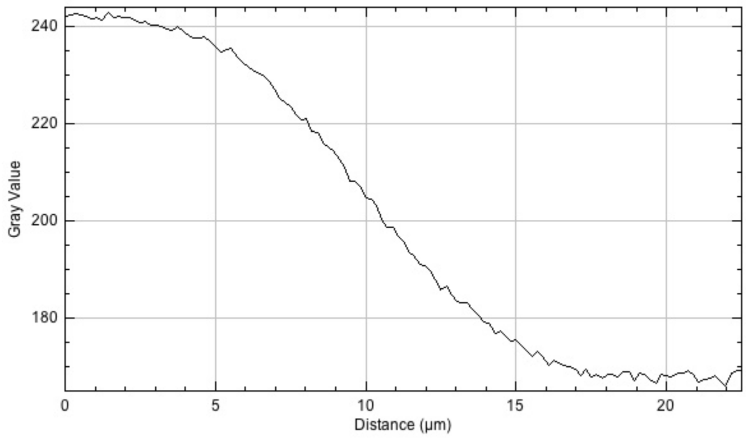

Grayscale profile from ImageJ

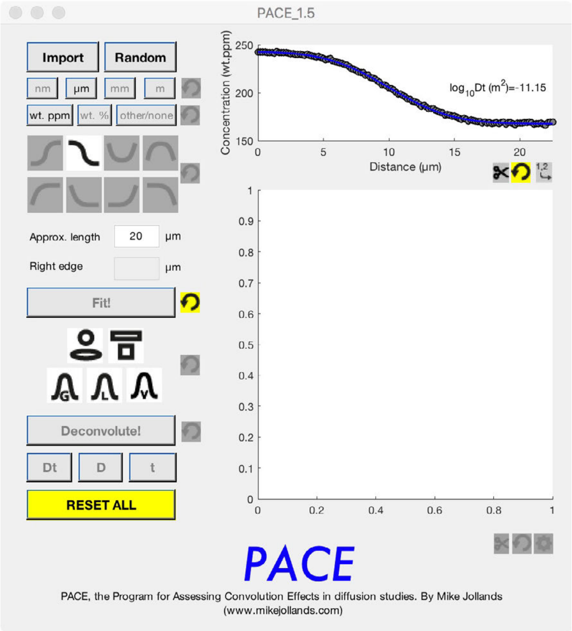

Profile fit in PACE

QtzIIb-10

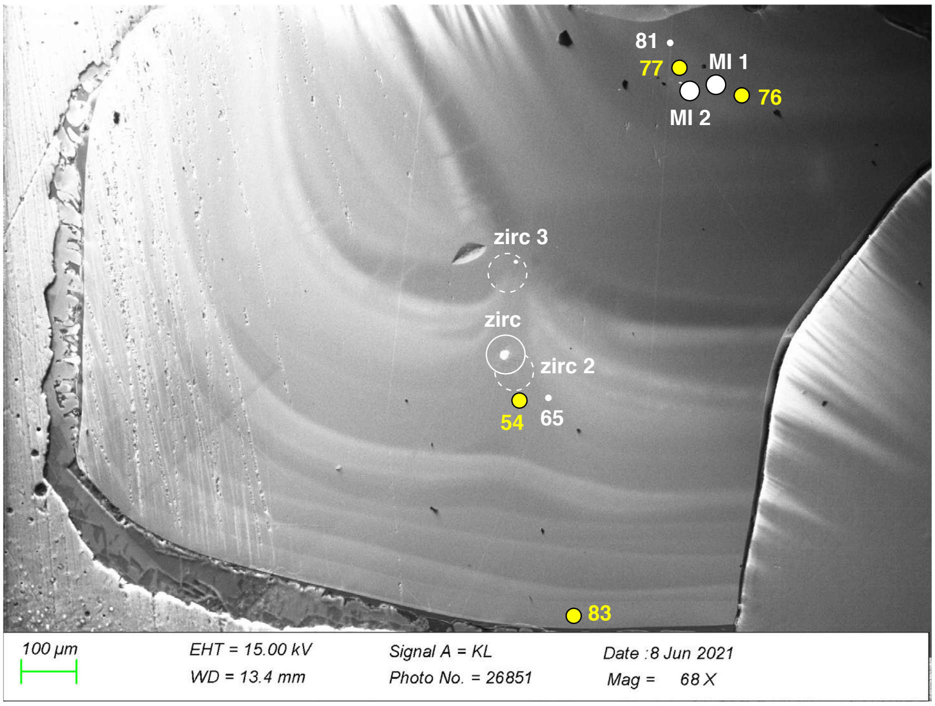

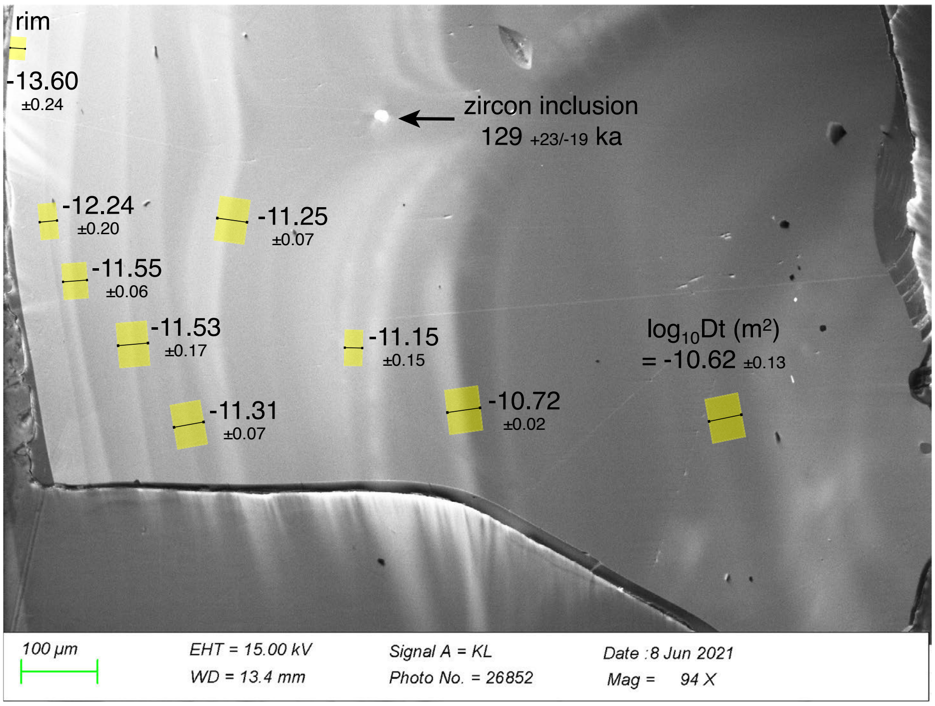

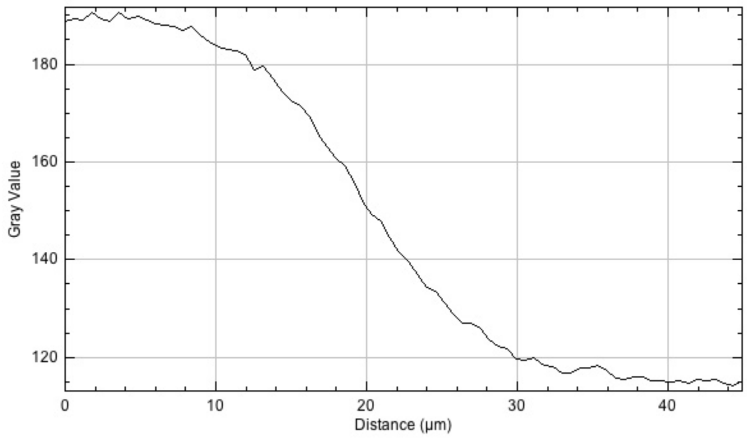

Grayscale profile from ImageJ

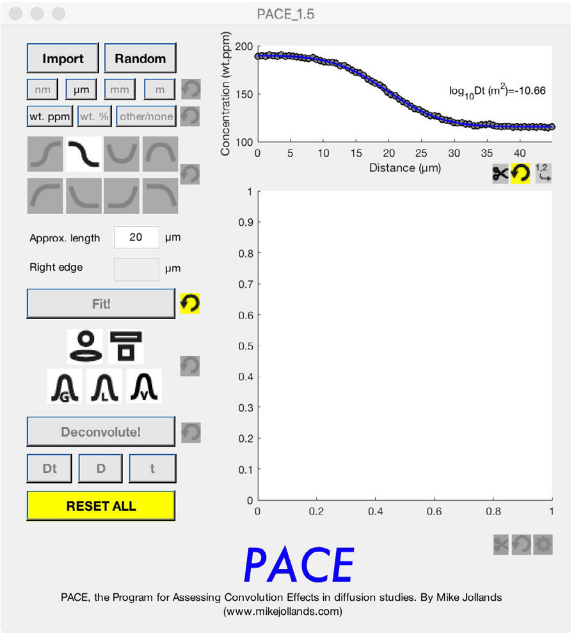

Profile fit in PACE

QtzV-7

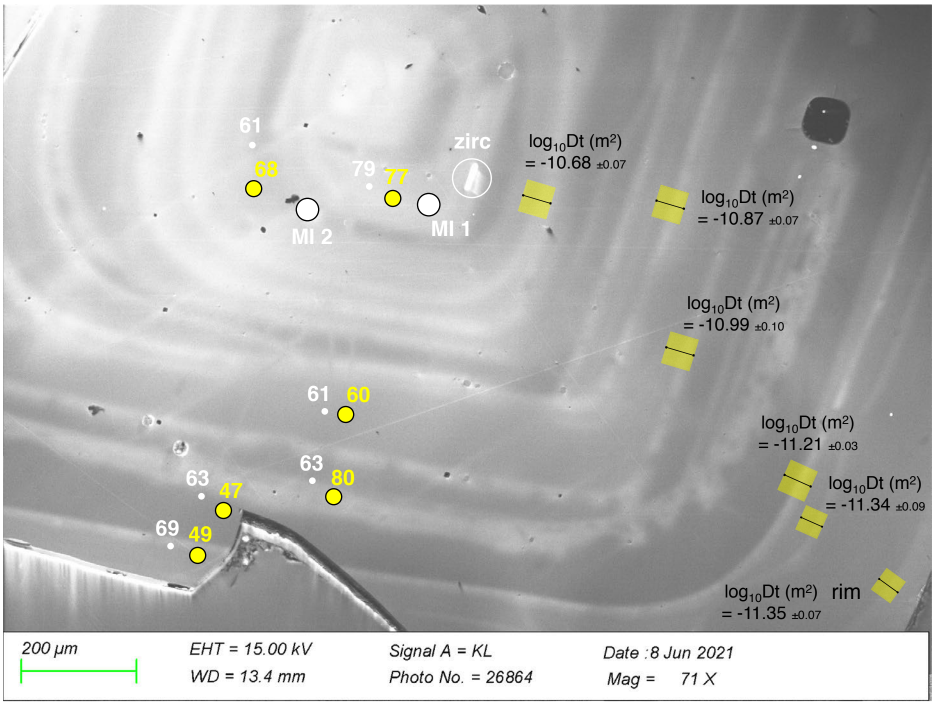

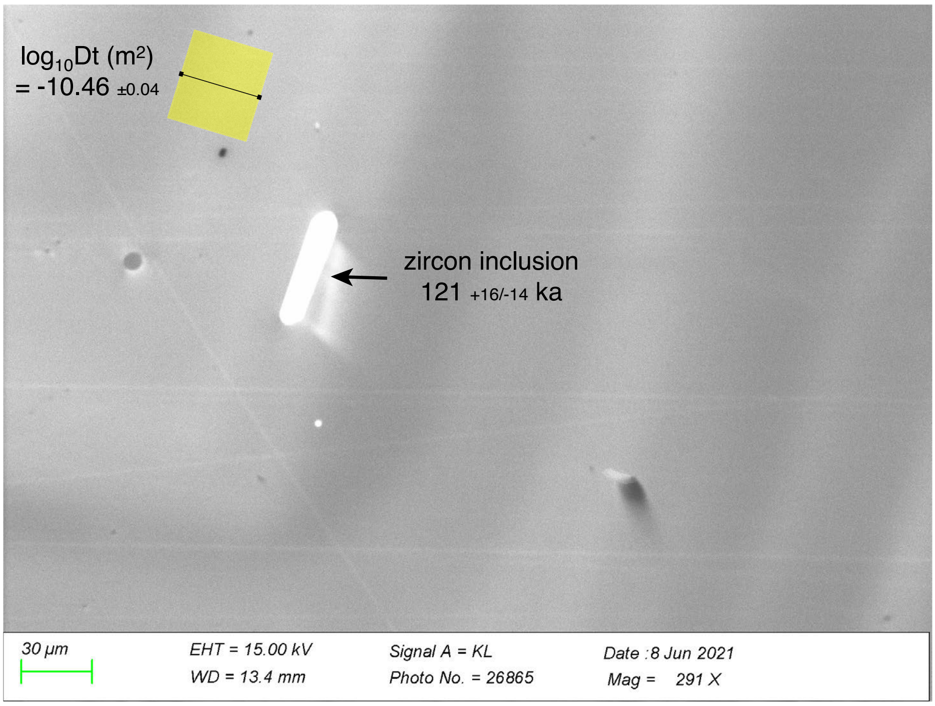

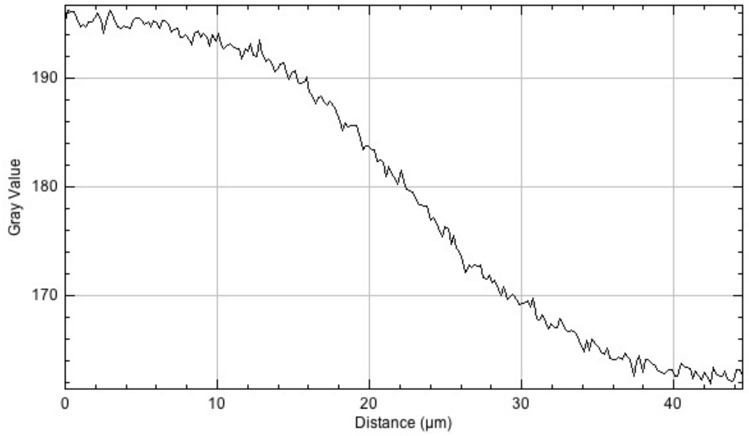

Grayscale profile from ImageJ

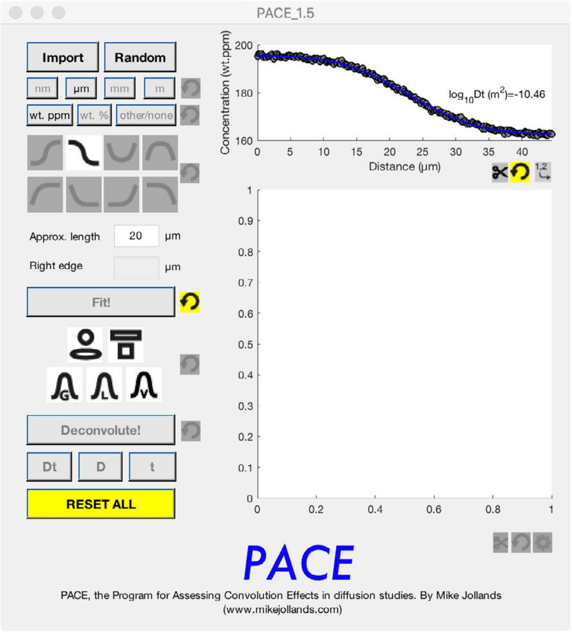

Profile fit in PACE

QtzV-17

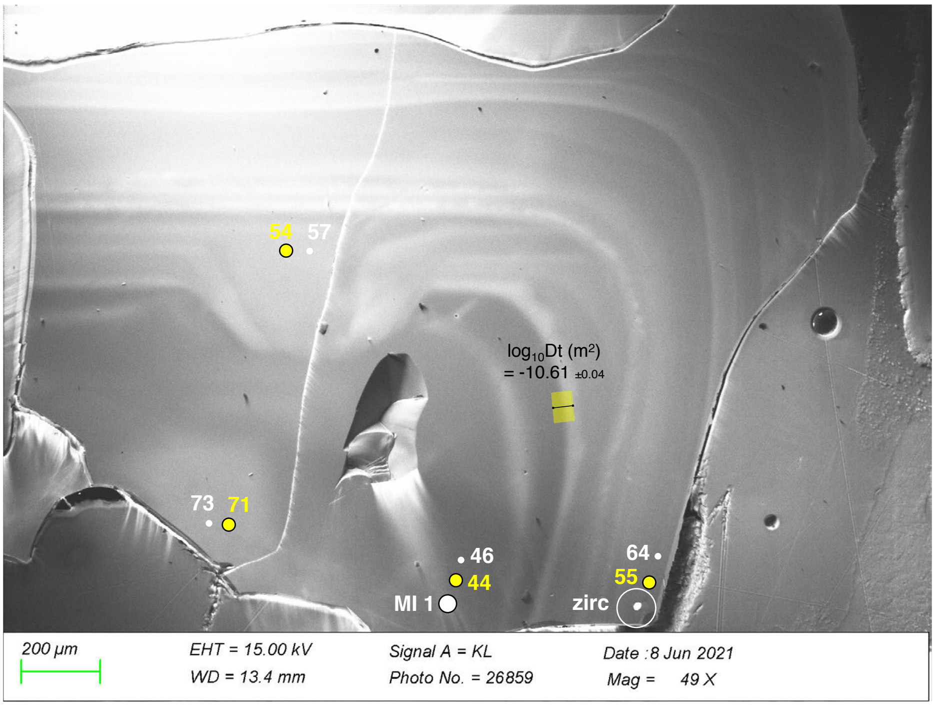

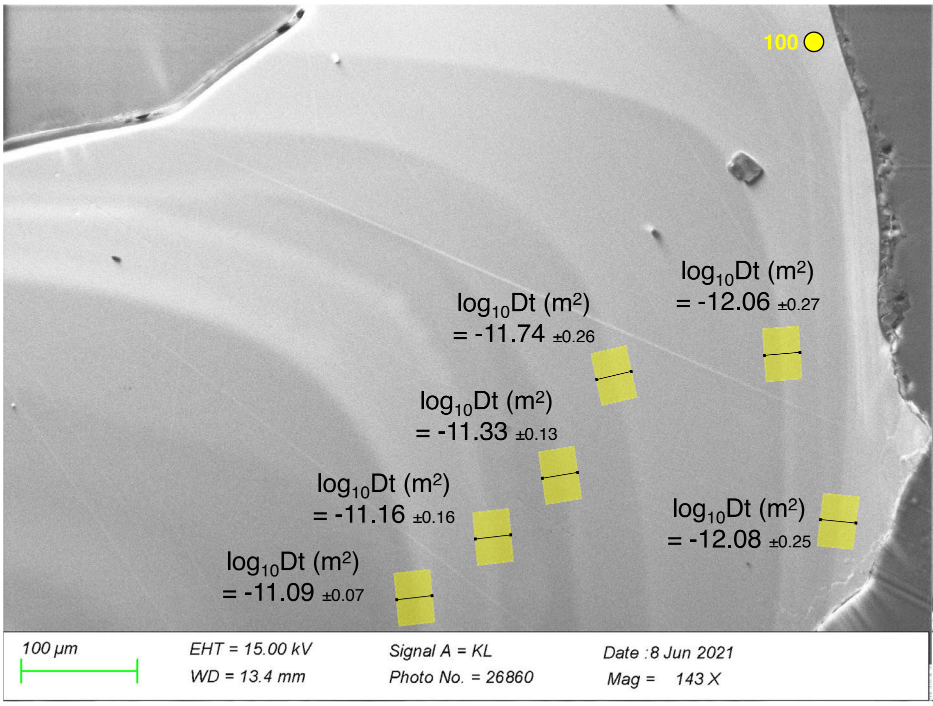

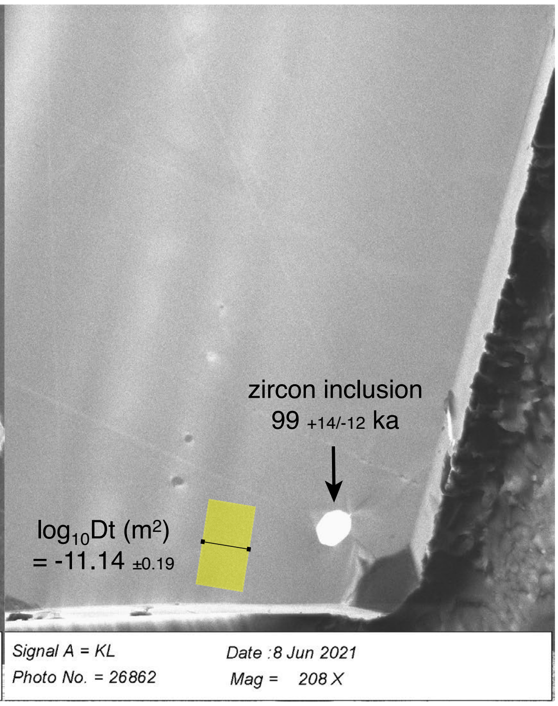

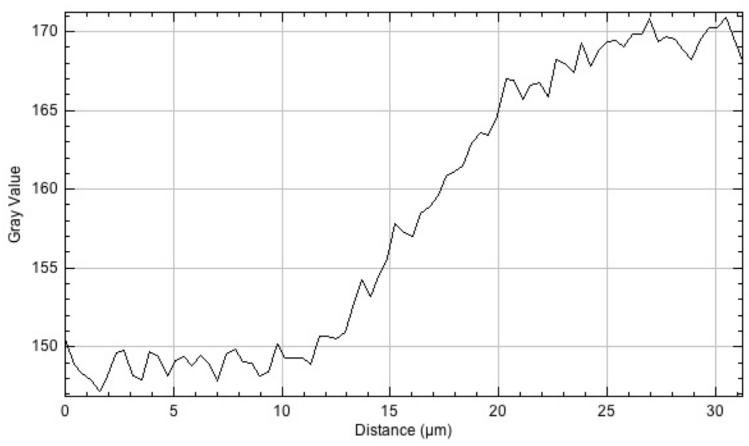

Grayscale profile from ImageJ

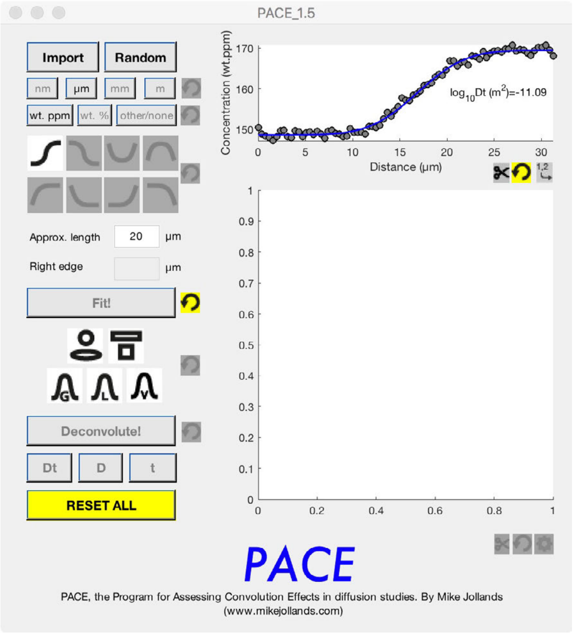

Profile fit in PACE

QtzV-18

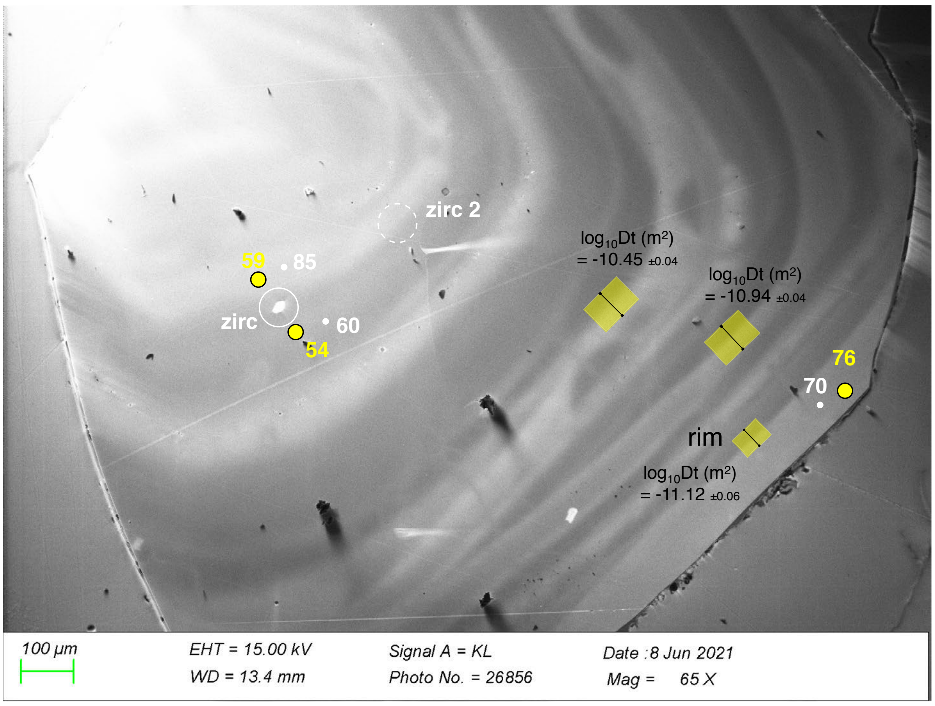

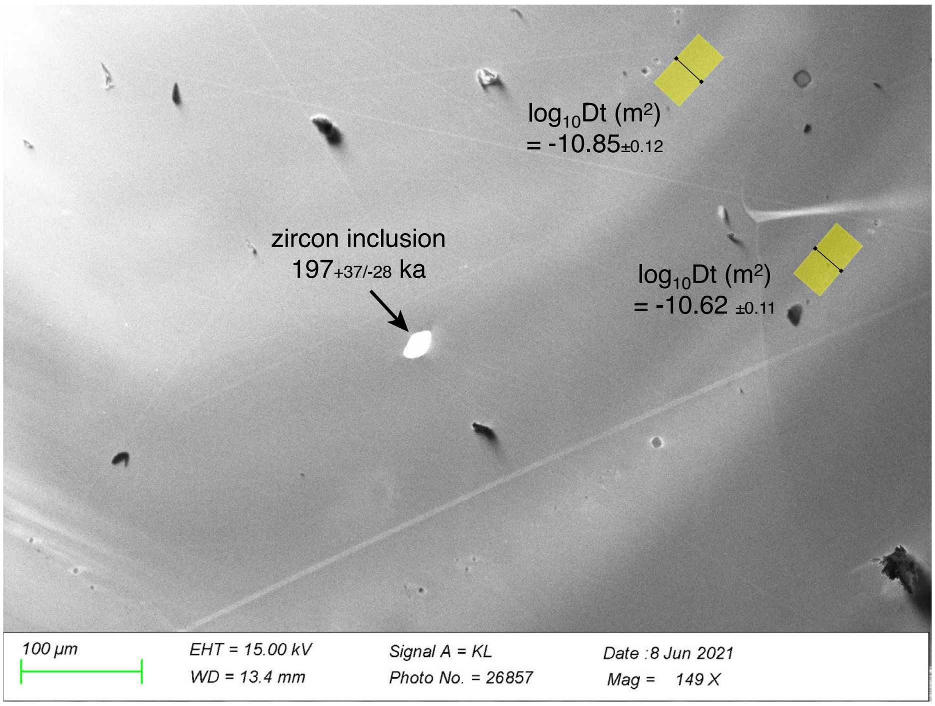

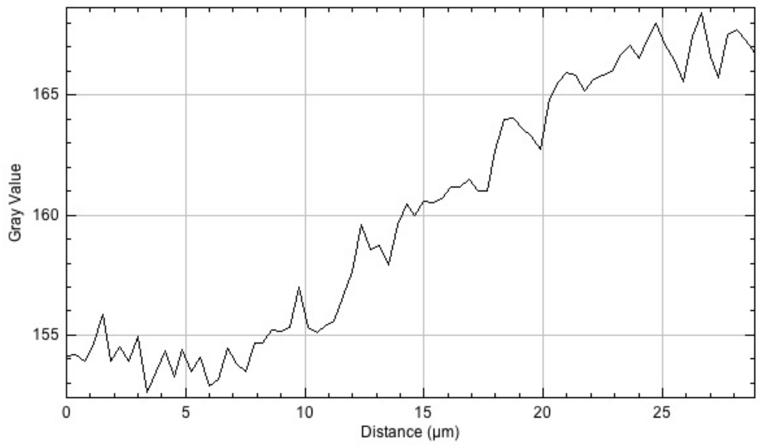

Grayscale profile from ImageJ

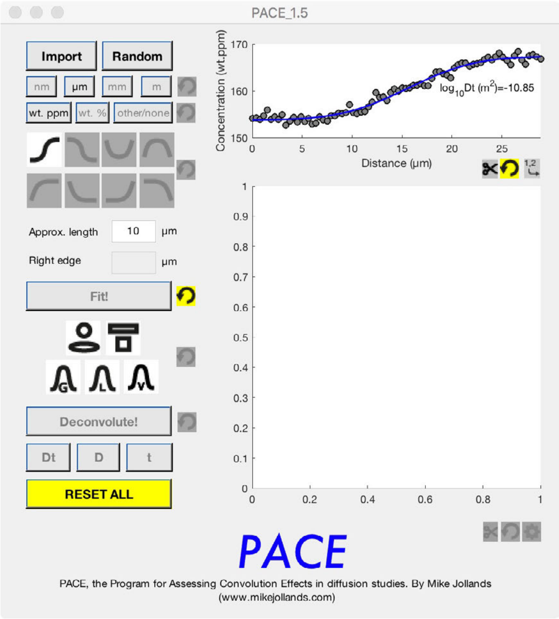

Profile fit in PACE

QtzX-3

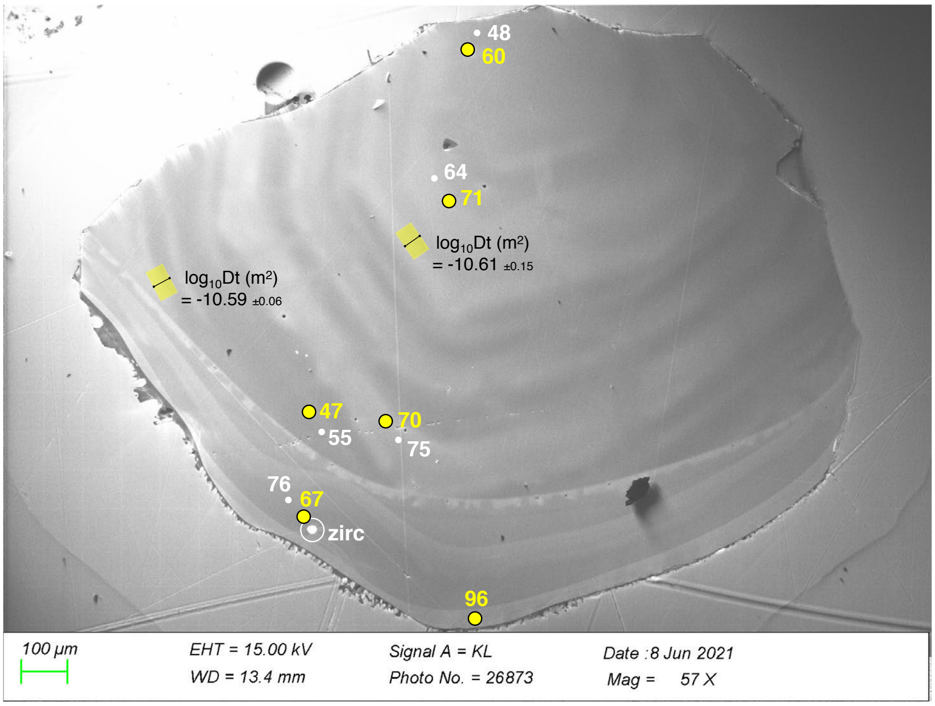

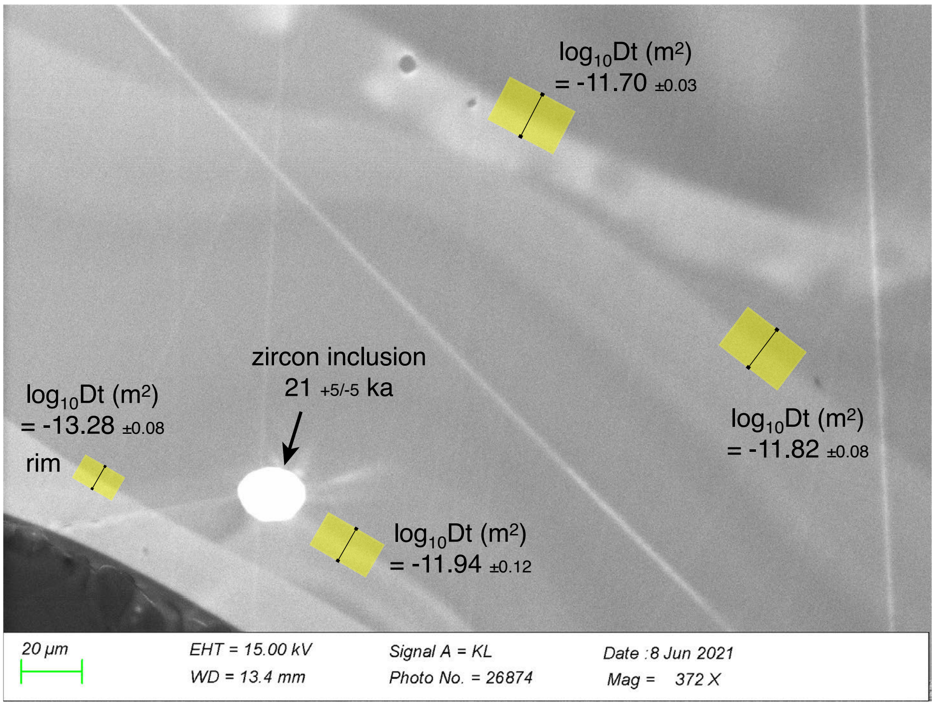

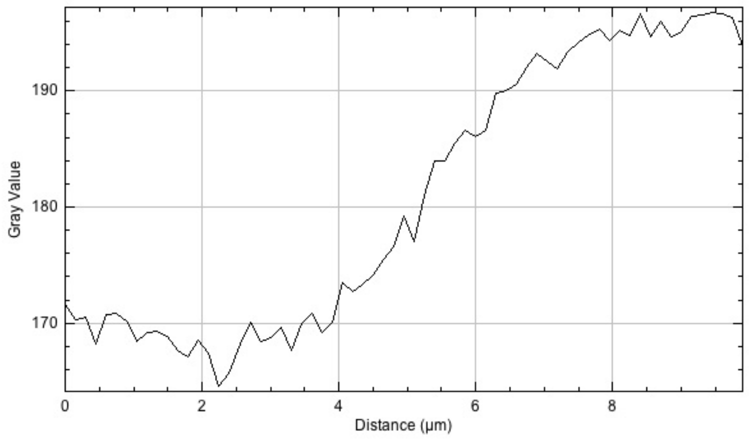

Grayscale profile from ImageJ

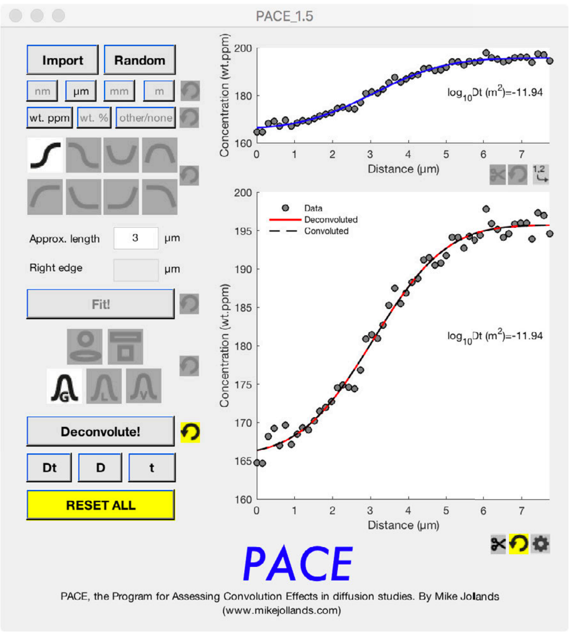

Profile fit in PACE
