# Supplementary material for: New constraints on Ti diffusion in quartz and the priming of silicic volcanic eruptions
Source: Nat Commun. 2023 Jul 17;14:4277. doi: 10.1038/s41467-023-39912-5 (PMC10352339; doi:10.1038/s41467-023-39912-5)
Supplement: Supplementary file 6 — Supplementary Data 3 [file 41467_2023_39912_MOESM6_ESM.pdf]

Amalia B6

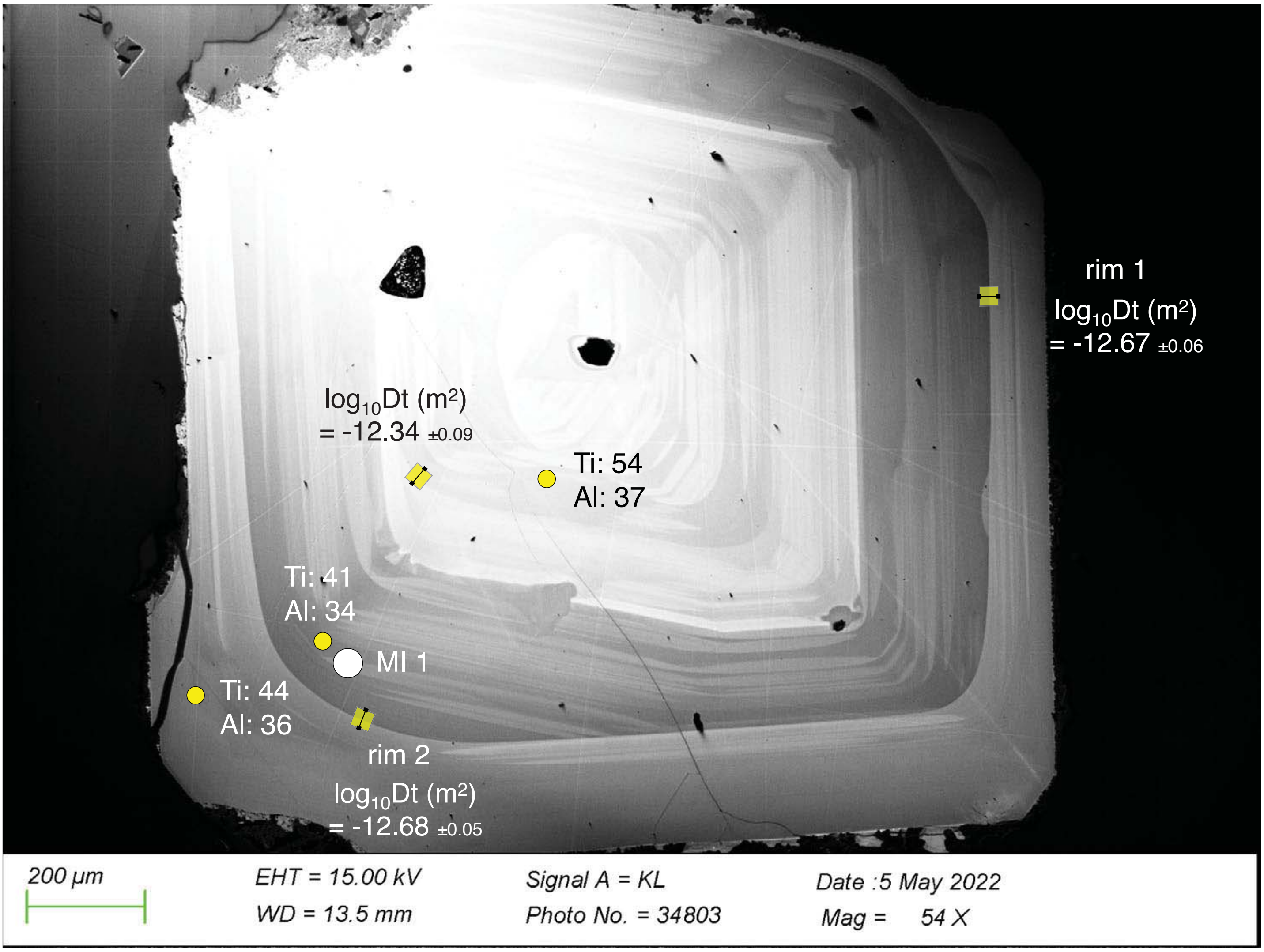

rim 1
log$_{10}$Dt (m^2)
= -12.67 ±0.06
log$_{10}$Dt (m^2)
= -12.34 ±0.09
Ti: 54
Al: 37
Ti: 41
Al: 34
MI 1
Ti: 44
Al: 36
rim 2
log$_{10}$Dt (m^2)
= -12.68 ±0.05
200 µm
EHT = 15.00 kV
WD = 13.5 mm
Signal A = KL
Photo No. = 34803
Date :5 May 2022
Mag = 54 X

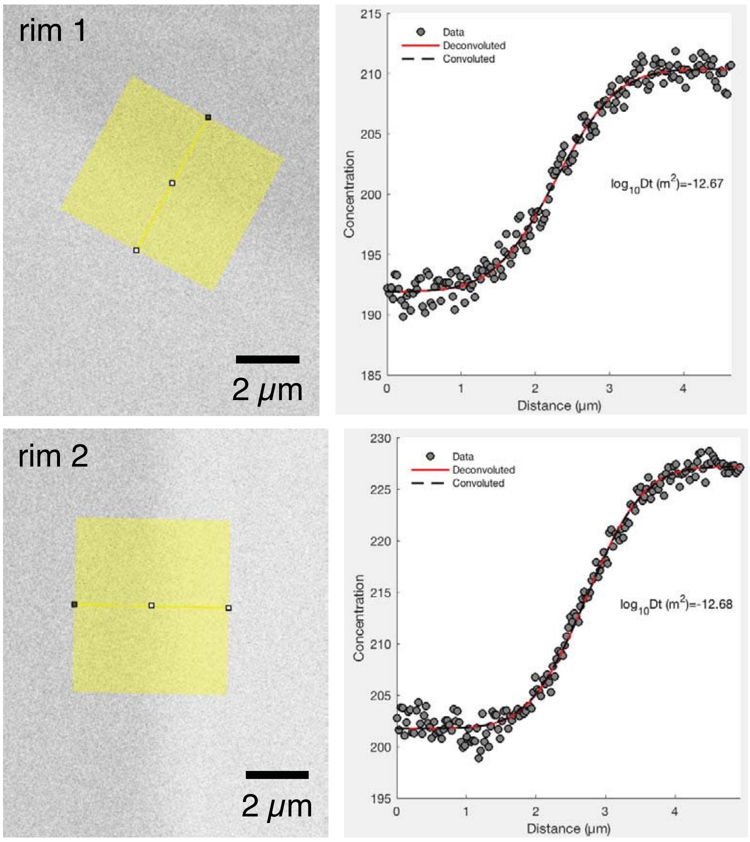

rim 1
2 µm
Data
Deconvoluted
Convoluted
log$_{10}$Dt (m^2)=-12.67
Concentration
Distance (µm)
rim 2
2 µm
Data
Deconvoluted
Convoluted
log$_{10}$Dt (m^2)=-12.68
Concentration
Distance (µm)

Amalia F1

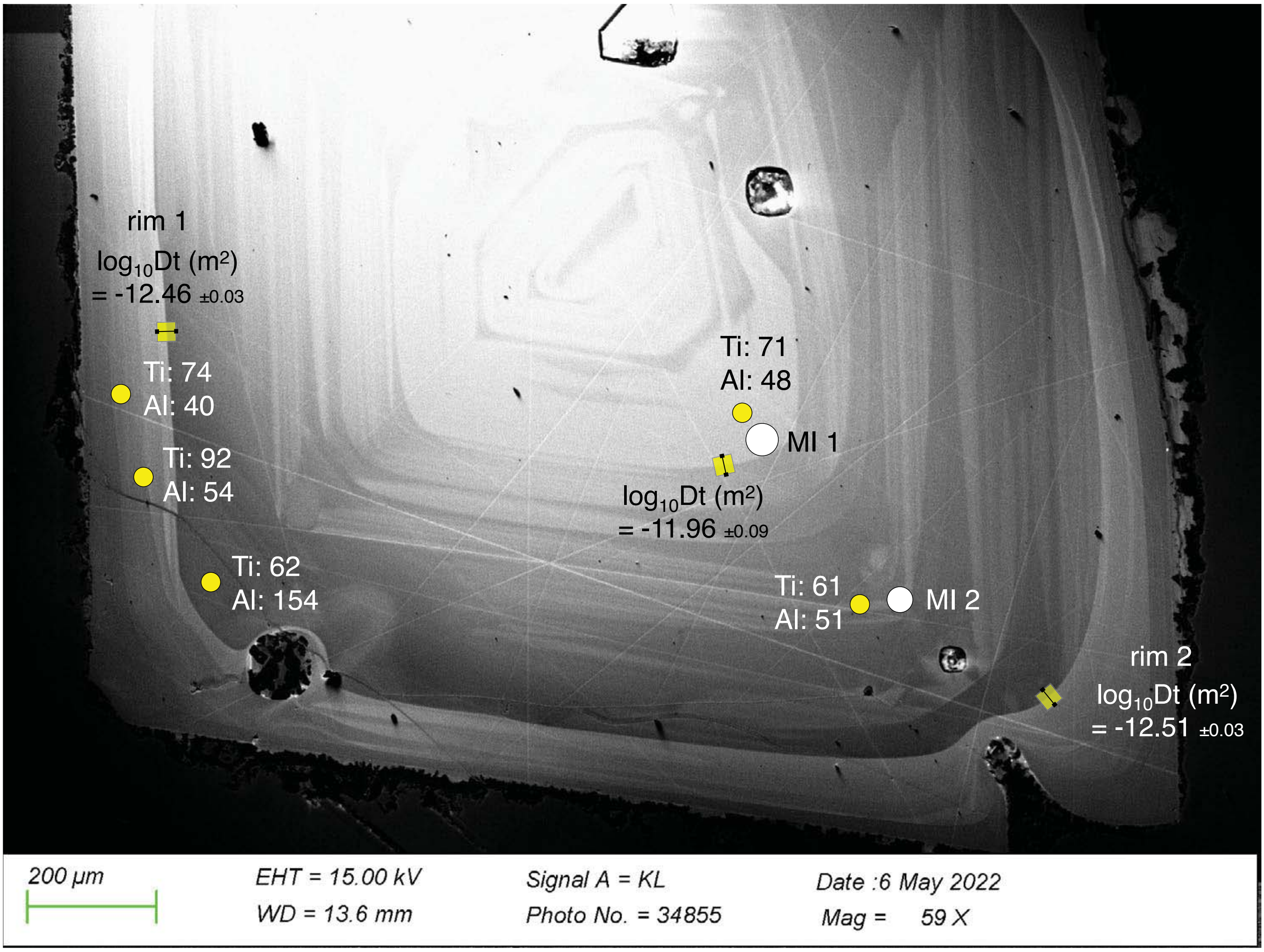

rim 1
log$_{10}$Dt (m^2)
= -12.46 ±0.03
Ti: 74
Al: 40
Ti: 92
Al: 54
Ti: 62
Al: 154
Ti: 71
Al: 48
MI 1
log$_{10}$Dt (m^2)
= -11.96 ±0.09
Ti: 61
Al: 51
MI 2
rim 2
log$_{10}$Dt (m^2)
= -12.51 ±0.03
200 μm
EHT = 15.00 kV
WD = 13.6 mm
Signal A = KL
Photo No. = 34855
Date :6 May 2022
Mag = 59 X

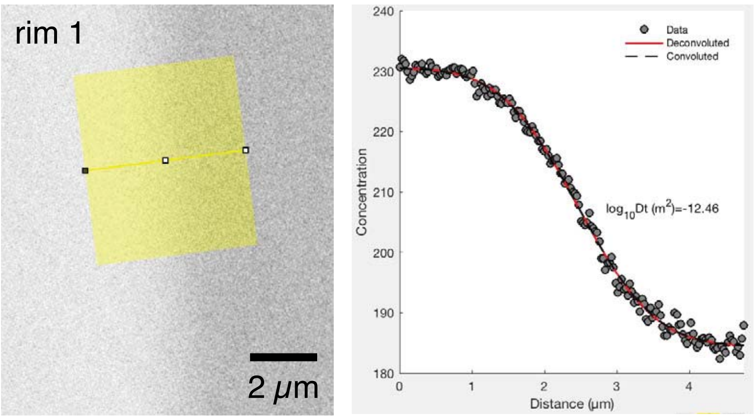

rim 1
2 μm
Data
Deconvoluted
Convoluted
log$_{10}$Dt (m^2)=-12.46
Concentration
Distance (μm)

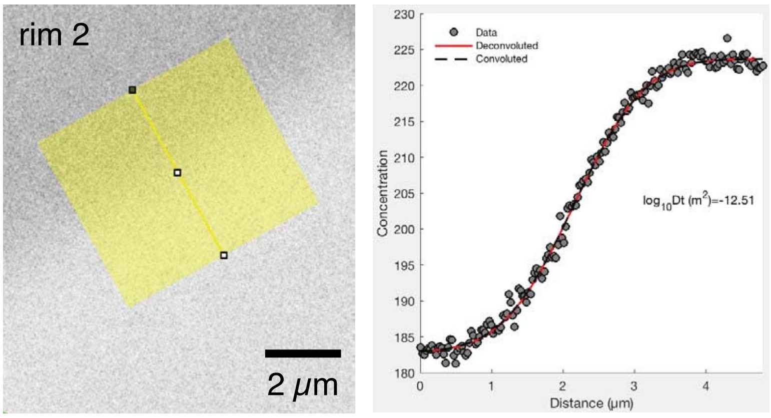

rim 2
2 μm
Data
Deconvoluted
Convoluted
log$_{10}$Dt (m^2)=-12.51
Concentration
Distance (μm)

Amalia I5

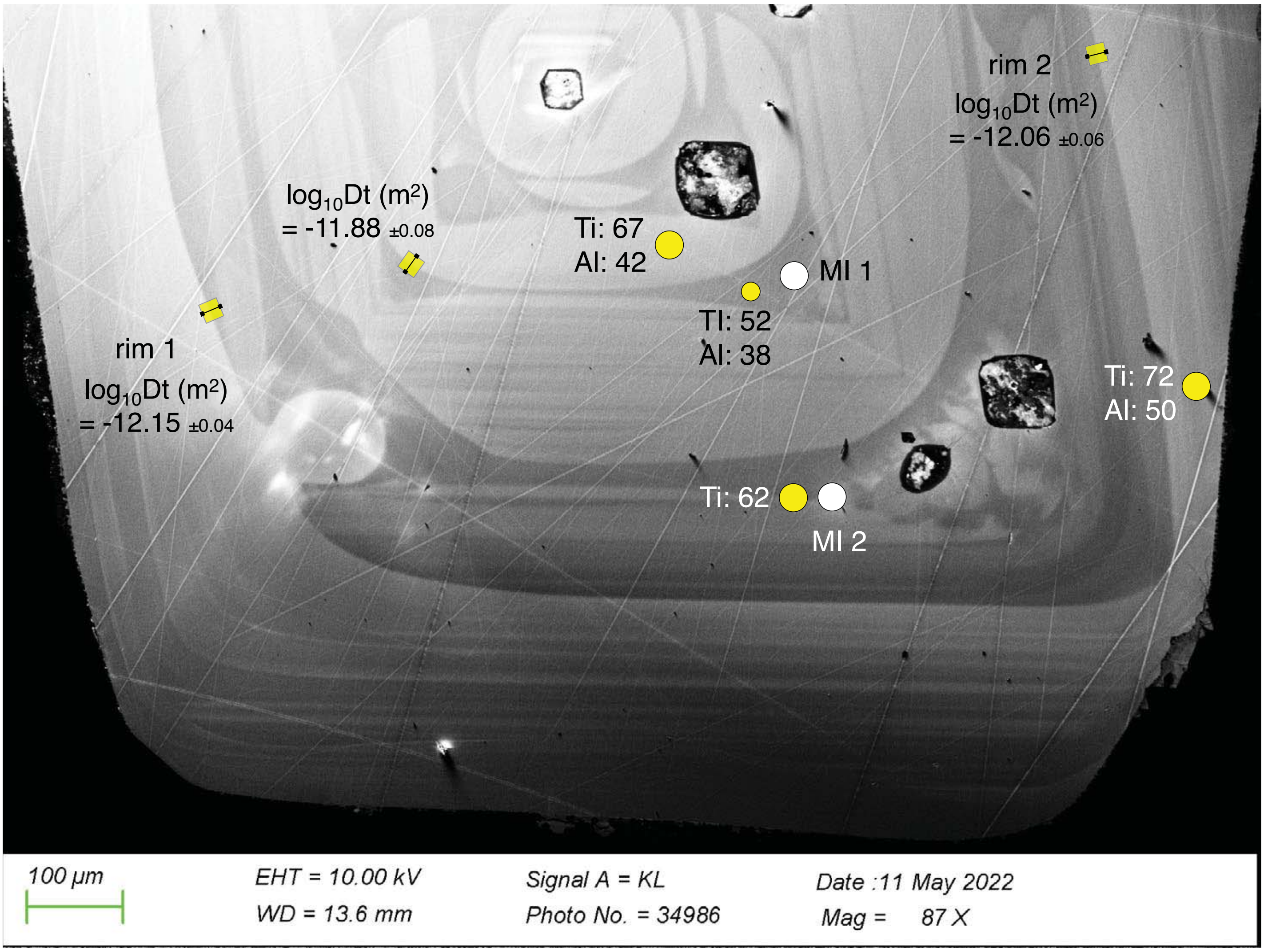
rim 2
$log_{10}Dt$ (m^2)
= -12.06 ±0.06
$log_{10}Dt$ (m^2)
= -11.88 ±0.08
Ti: 67
Al: 42
MI 1
TI: 52
Al: 38
rim 1
$log_{10}Dt$ (m^2)
= -12.15 ±0.04
Ti: 72
Al: 50
Ti: 62
MI 2
100 μm
EHT = 10.00 kV
WD = 13.6 mm
Signal A = KL
Photo No. = 34986
Date :11 May 2022
Mag = 87 X

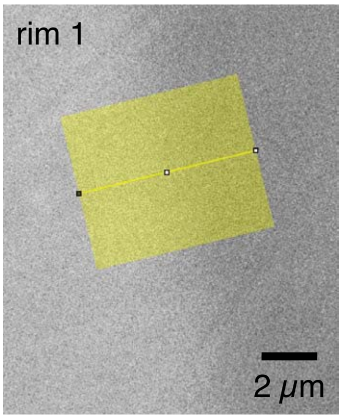
rim 1
2 μm

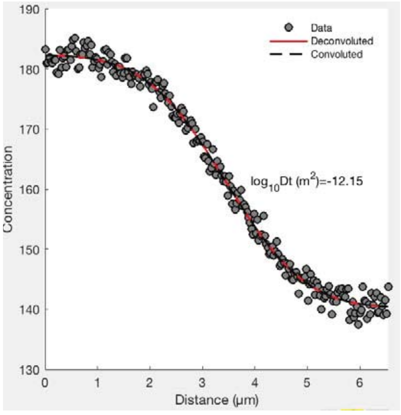
Data
Deconvoluted
Convoluted
$log_{10}Dt$ (m^2)=-12.15
Concentration
Distance (μm)

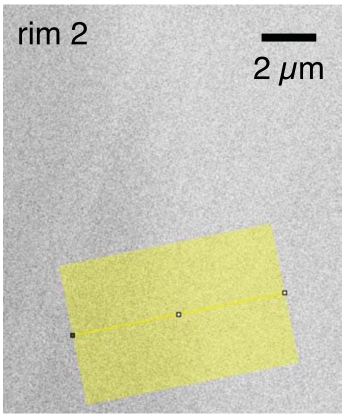
rim 2
2 μm

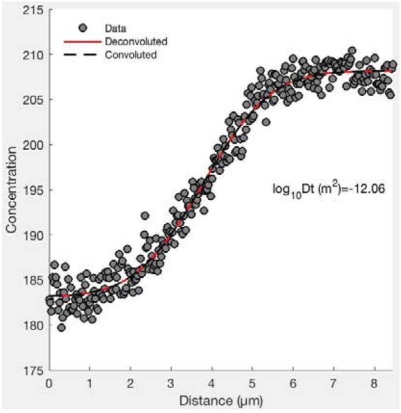
Data
Deconvoluted
Convoluted
$log_{10}Dt$ (m^2)=-12.06
Concentration
Distance (μm)

Bandelier AB

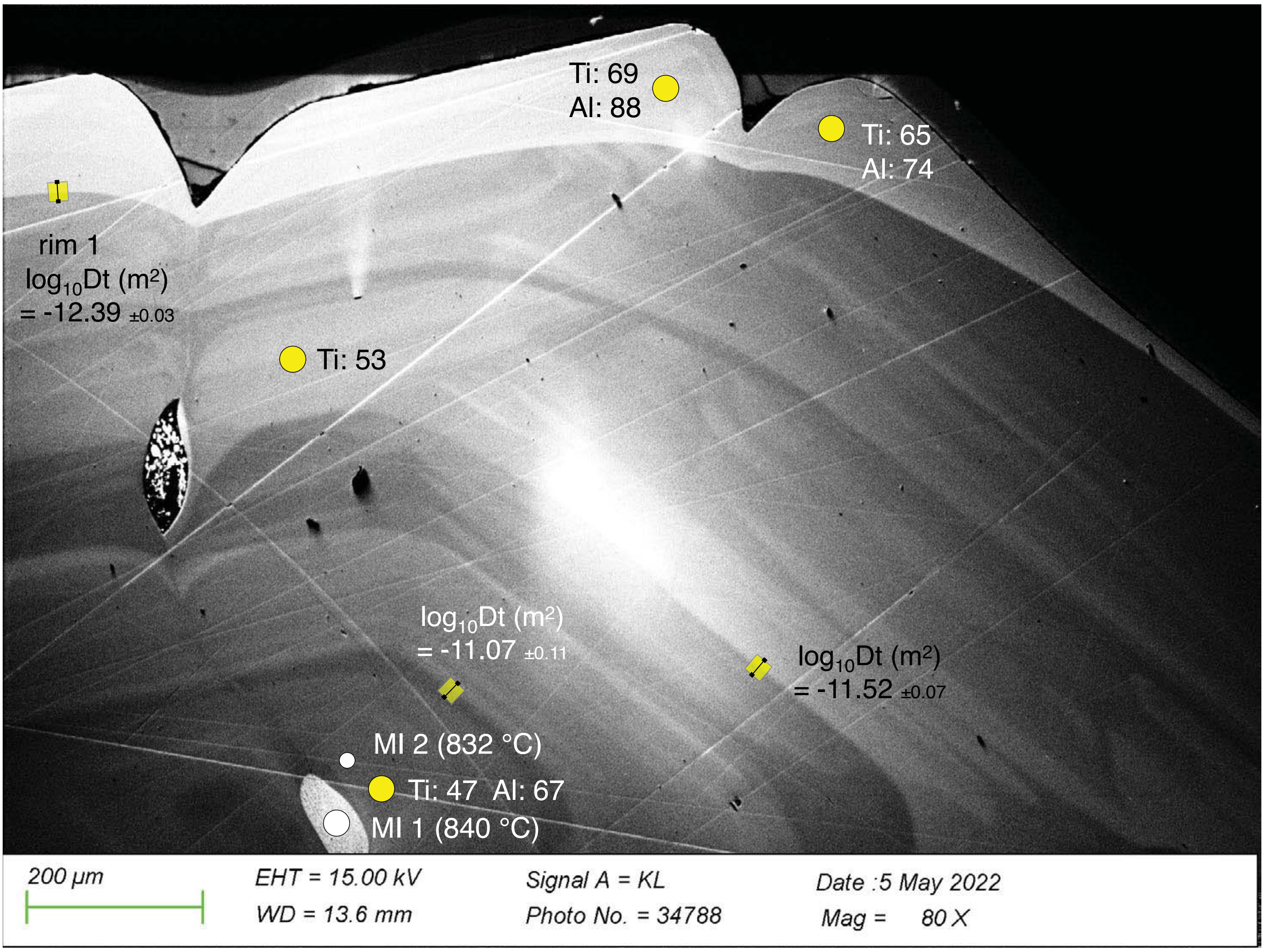

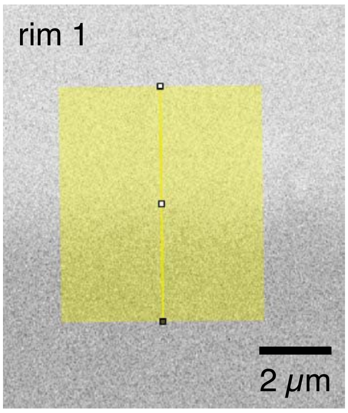

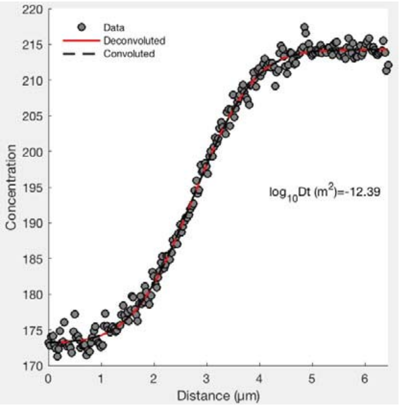

Bandelier AG

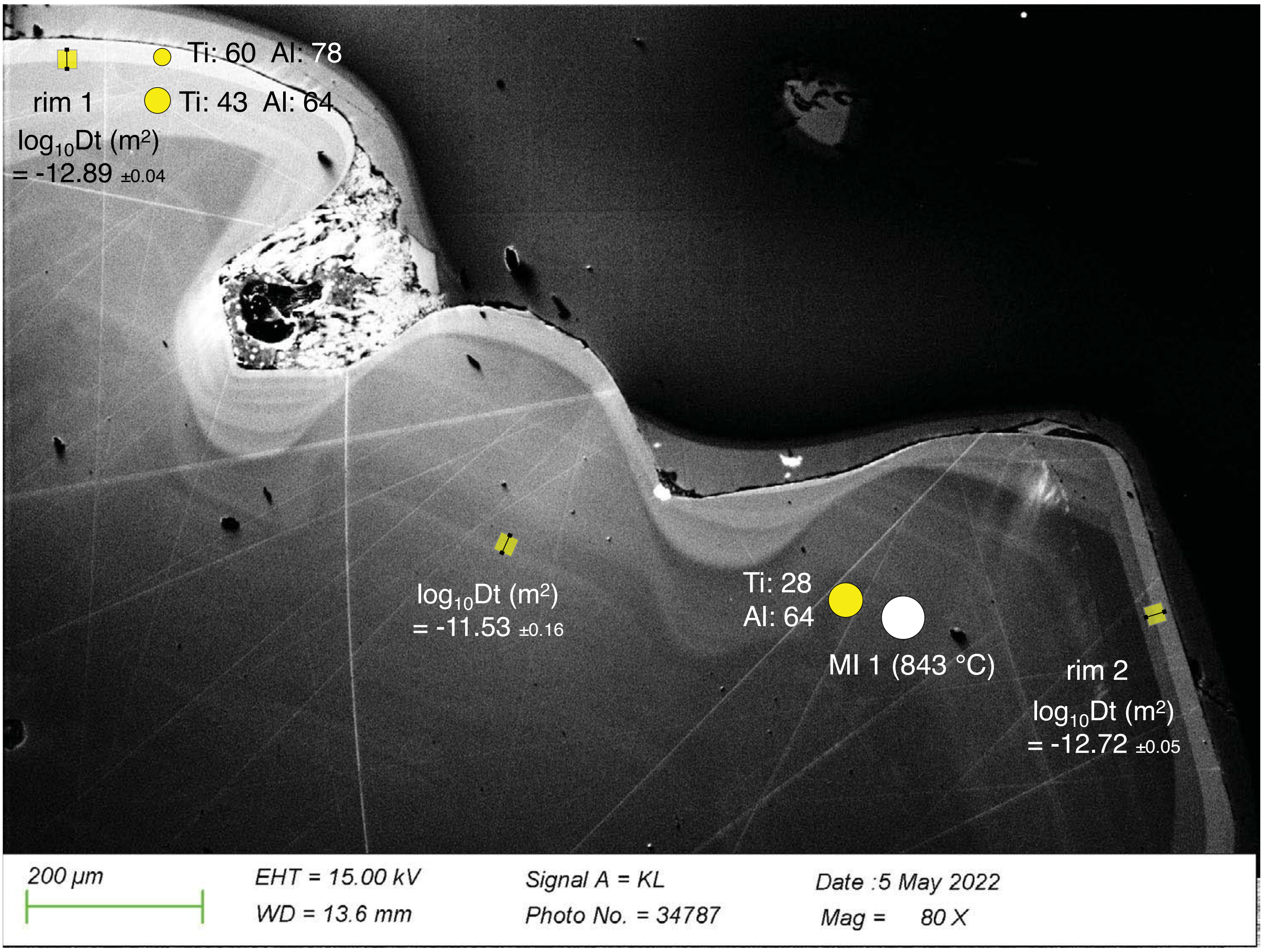

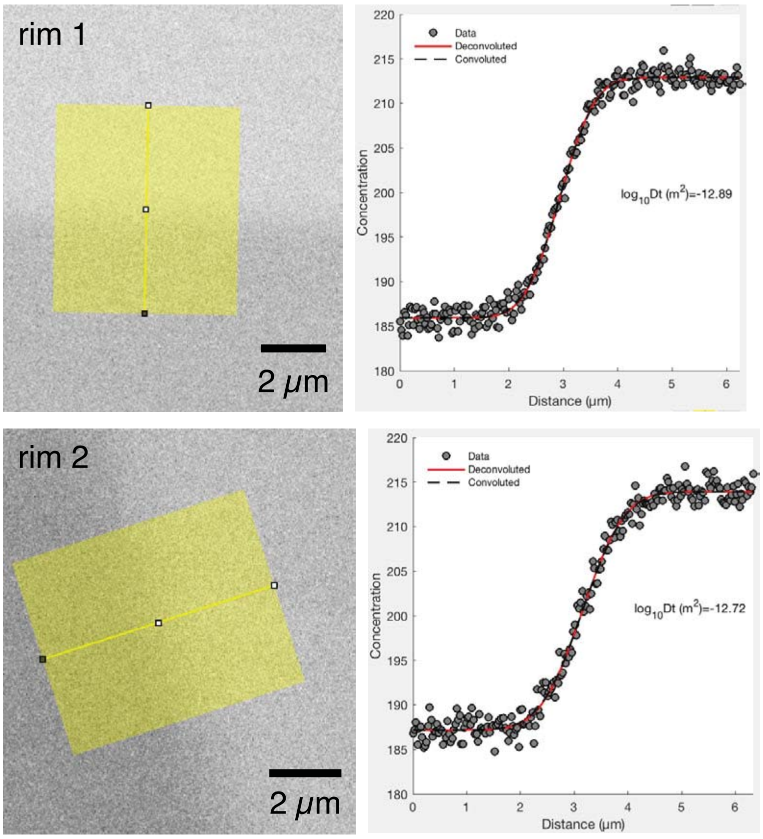

Bandelier BA

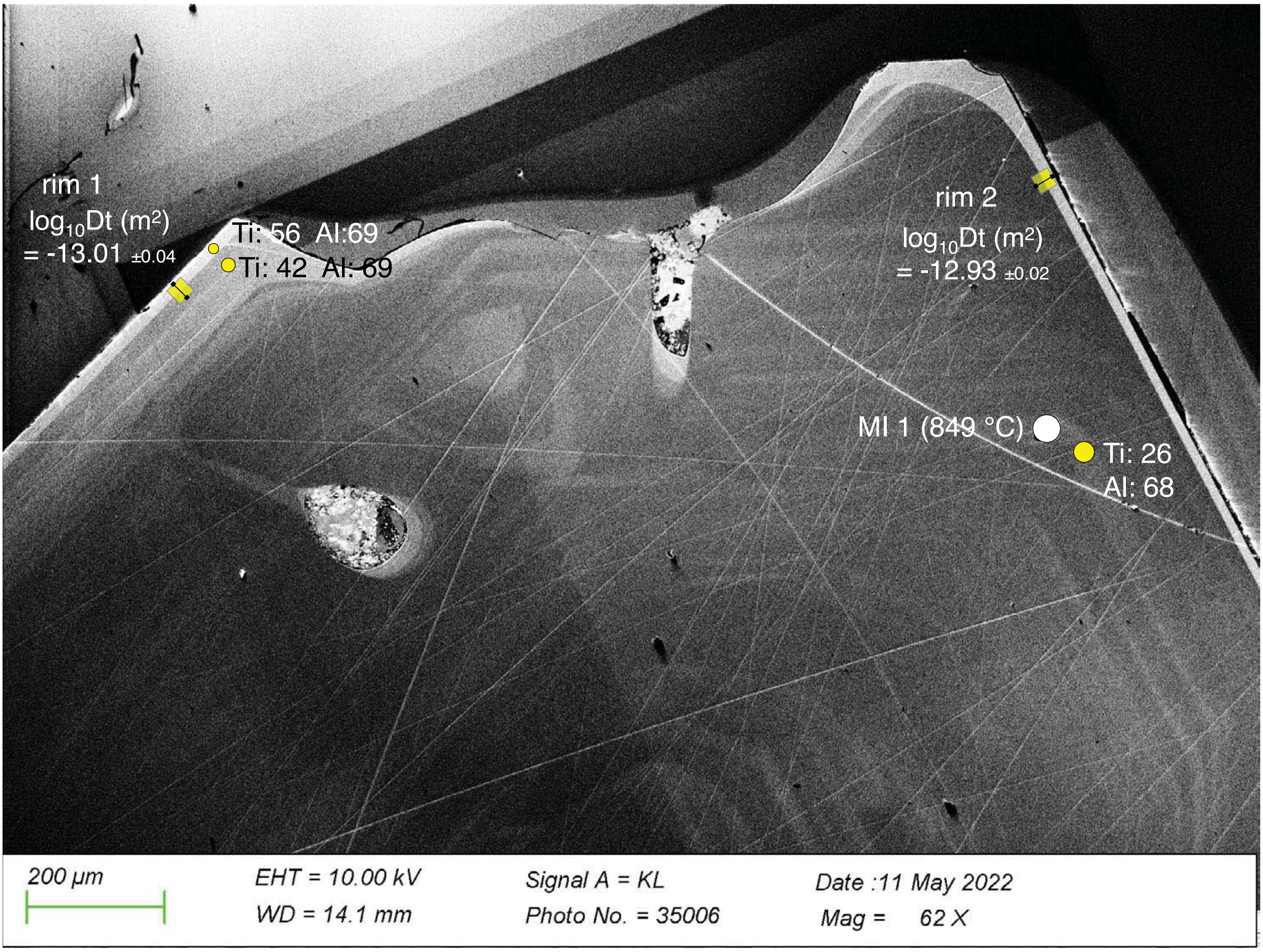

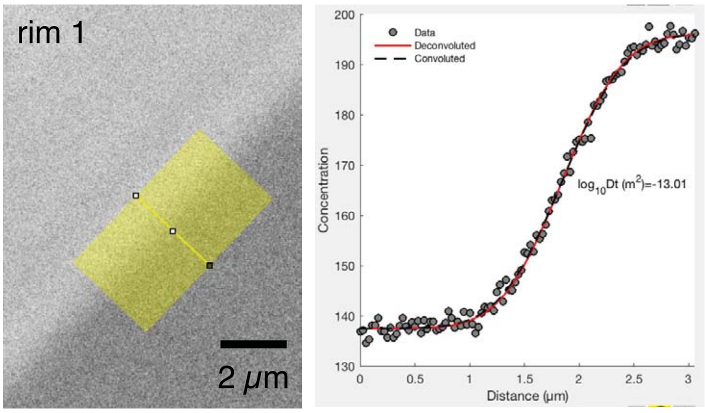

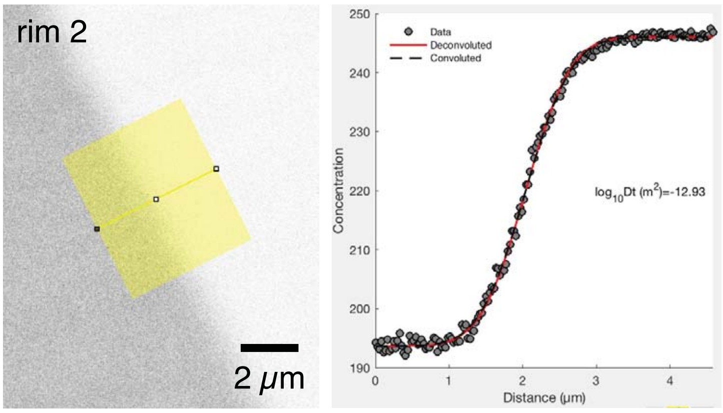

Bandelier BB

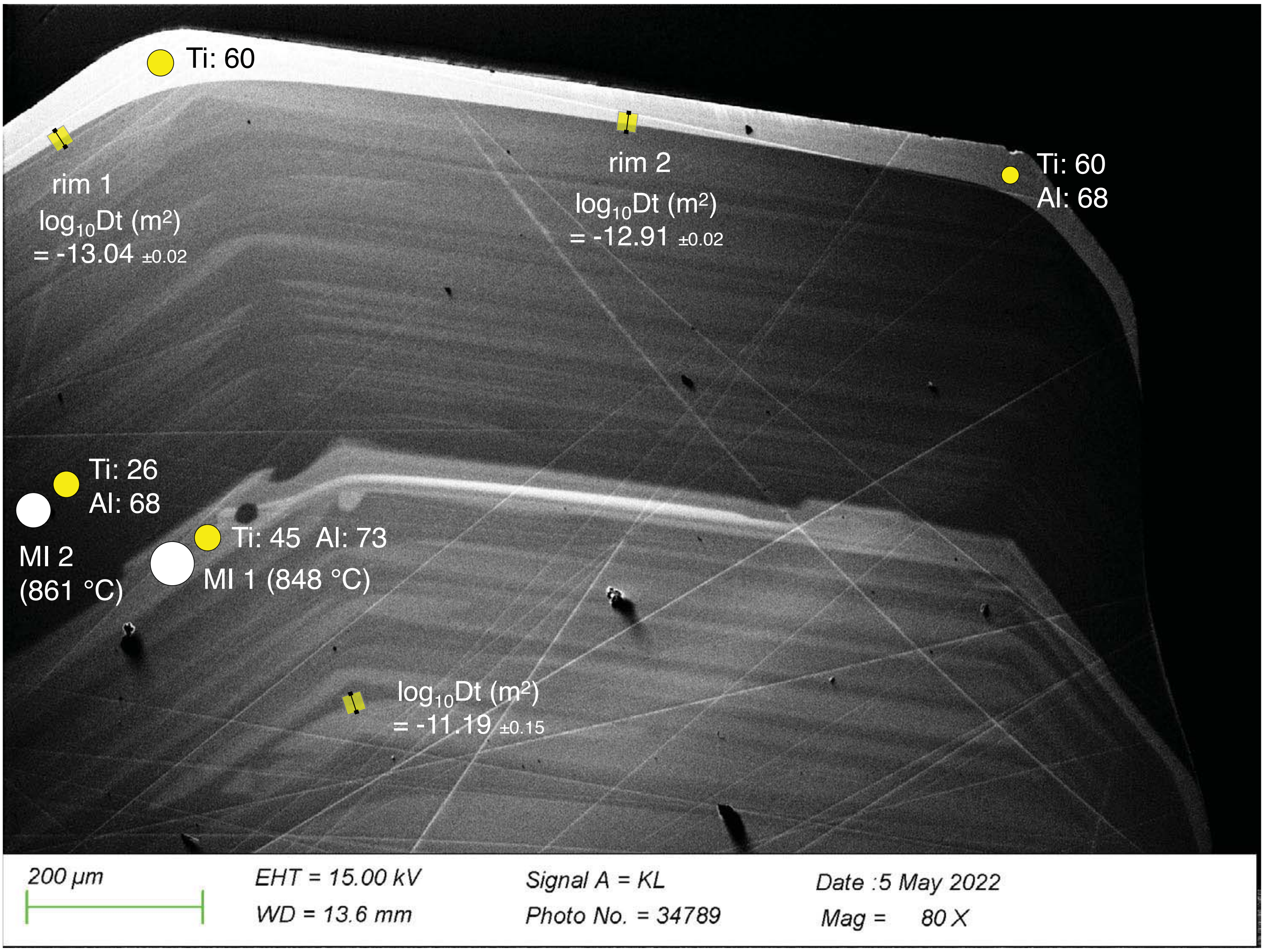

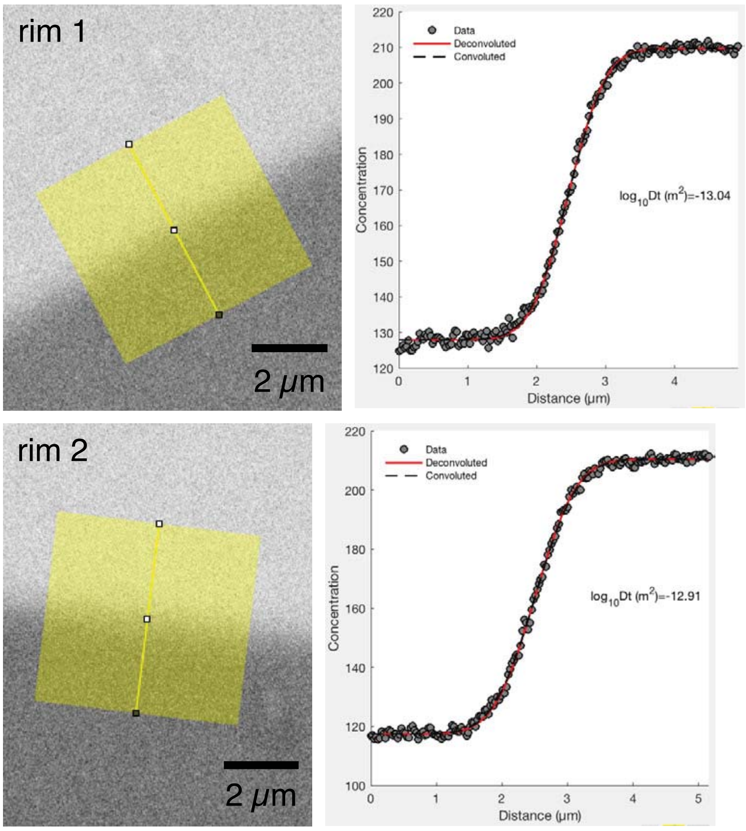

Cotton1 Q1

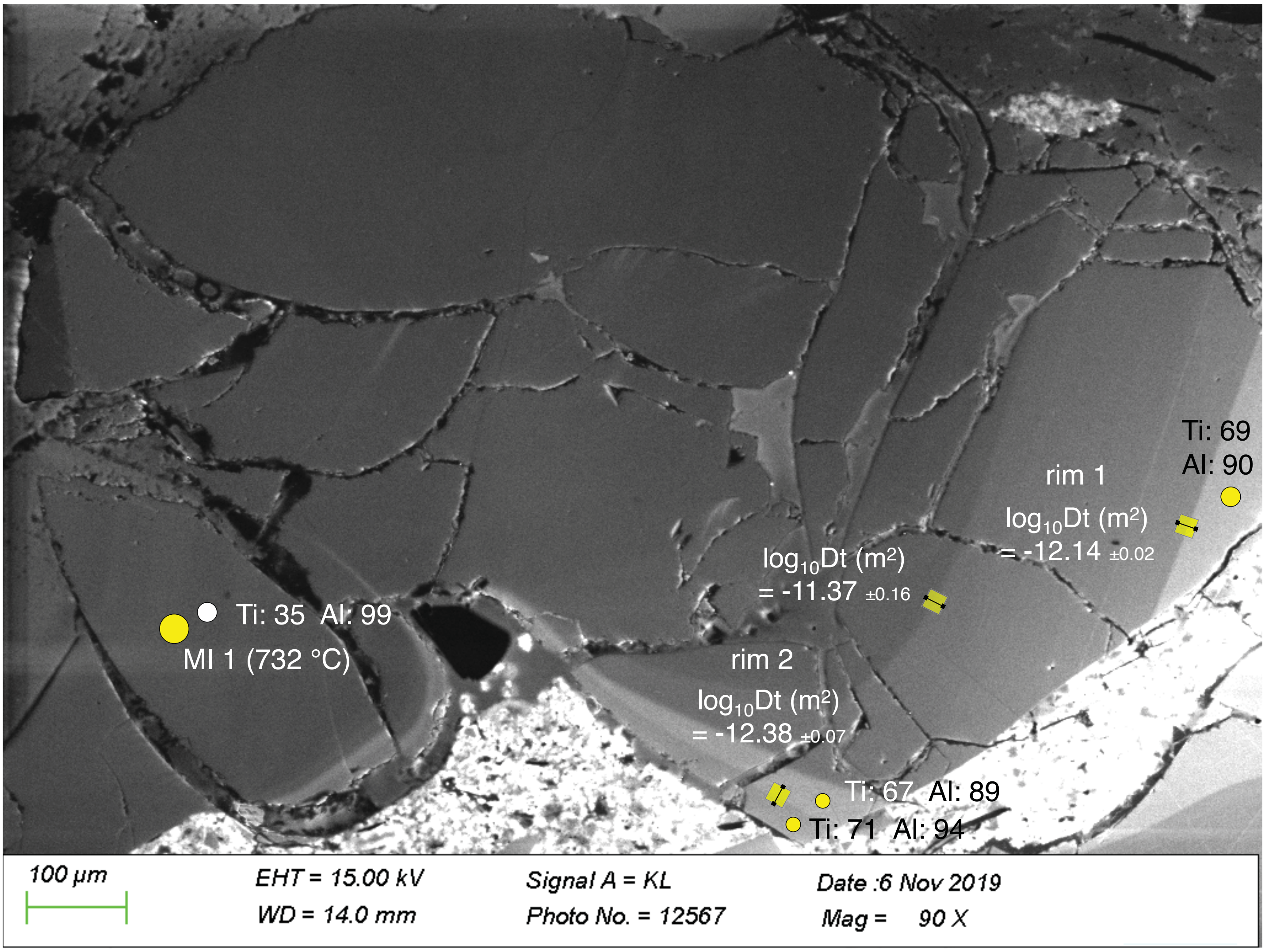
Ti: 69
Al: 90
rim 1
log10Dt (m2)
= -12.14 ±0.02
log10Dt (m2)
= -11.37 ±0.16
Ti: 35 Al: 99
MI 1 (732 °C)
rim 2
log10Dt (m2)
= -12.38 ±0.07
Ti: 67 Al: 89
Ti: 71 Al: 94
100 µm
EHT = 15.00 kV
WD = 14.0 mm
Signal A = KL
Photo No. = 12567
Date :6 Nov 2019
Mag = 90 X

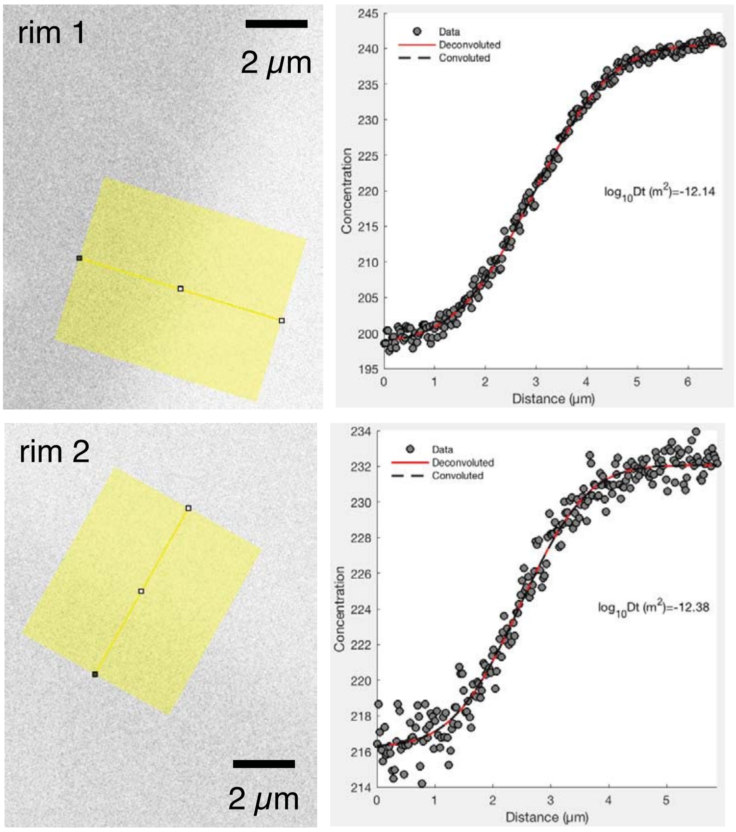
rim 1
2 µm
Data
Deconvoluted
Convoluted
log10Dt (m2)=-12.14
Concentration
Distance (µm)
rim 2
2 µm
Data
Deconvoluted
Convoluted
log10Dt (m2)=-12.38
Concentration
Distance (µm)

Cotton1 Q2

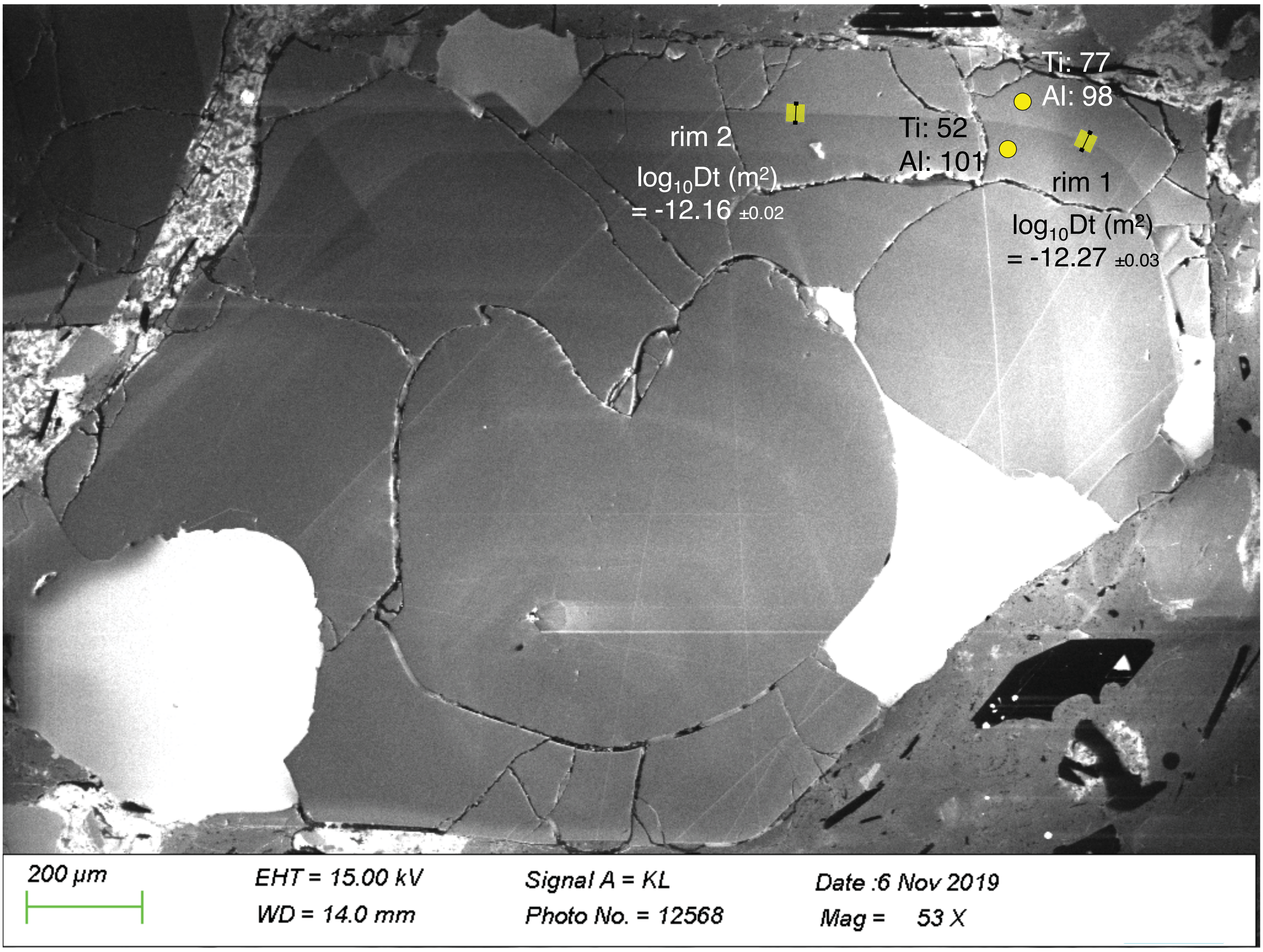

Ti: 77
Al: 98
rim 2
log10Dt (m2)
= -12.16 ±0.02
Ti: 52
Al: 101
rim 1
log10Dt (m2)
= -12.27 ±0.03
200 µm
EHT = 15.00 kV
WD = 14.0 mm
Signal A = KL
Photo No. = 12568
Date :6 Nov 2019
Mag = 53 X

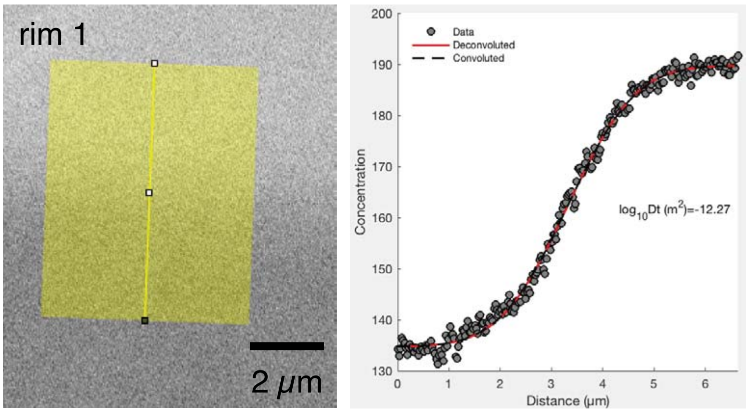

rim 1
2 µm
Data
Deconvoluted
Convoluted
Concentration
Distance (µm)
log10Dt (m2)=-12.27

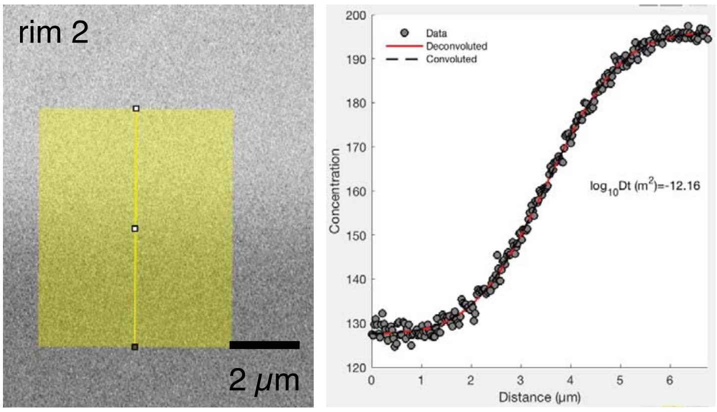

rim 2
2 µm
Data
Deconvoluted
Convoluted
Concentration
Distance (µm)
log10Dt (m2)=-12.16

Cotton1 Q6

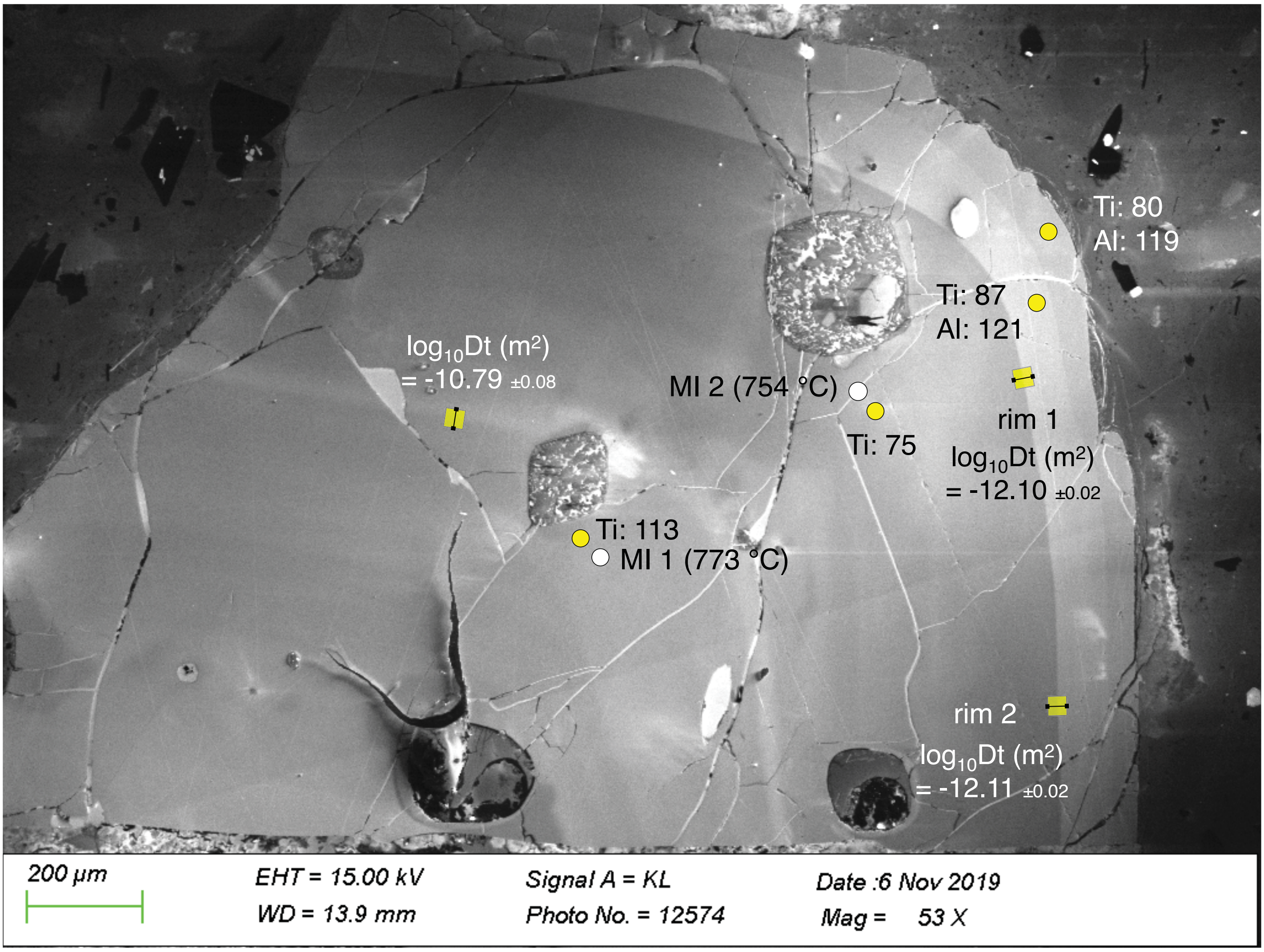
Ti: 80
Al: 119
Ti: 87
Al: 121
$log_{10}Dt$ (m^2)
= -10.79 ±0.08
MI 2 (754 °C)
Ti: 75
rim 1
$log_{10}Dt$ (m^2)
= -12.10 ±0.02
Ti: 113
MI 1 (773 °C)
rim 2
$log_{10}Dt$ (m^2)
= -12.11 ±0.02
200 μm
EHT = 15.00 kV
WD = 13.9 mm
Signal A = KL
Photo No. = 12574
Date :6 Nov 2019
Mag = 53 X

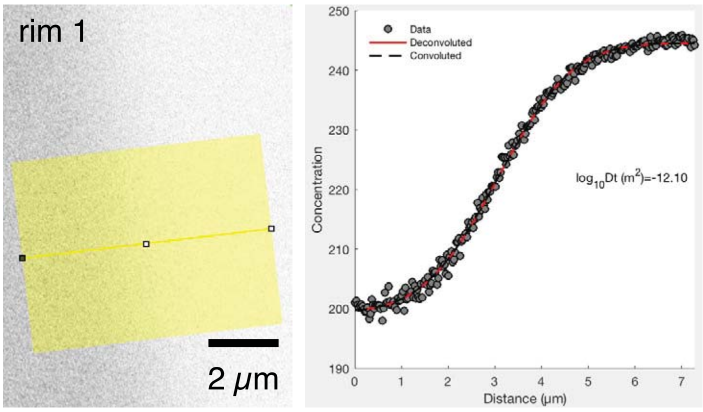
rim 1
2 μm
Data
Deconvoluted
Convoluted
$log_{10}Dt$ (m^2)=-12.10
Concentration
Distance (μm)

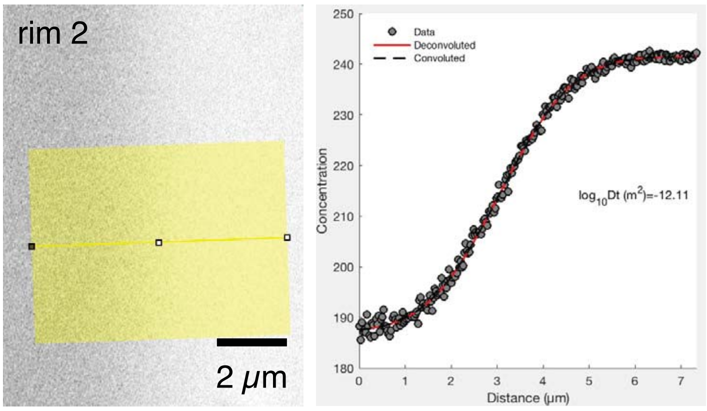
rim 2
2 μm
Data
Deconvoluted
Convoluted
$log_{10}Dt$ (m^2)=-12.11
Concentration
Distance (μm)

Hiko1 Q4

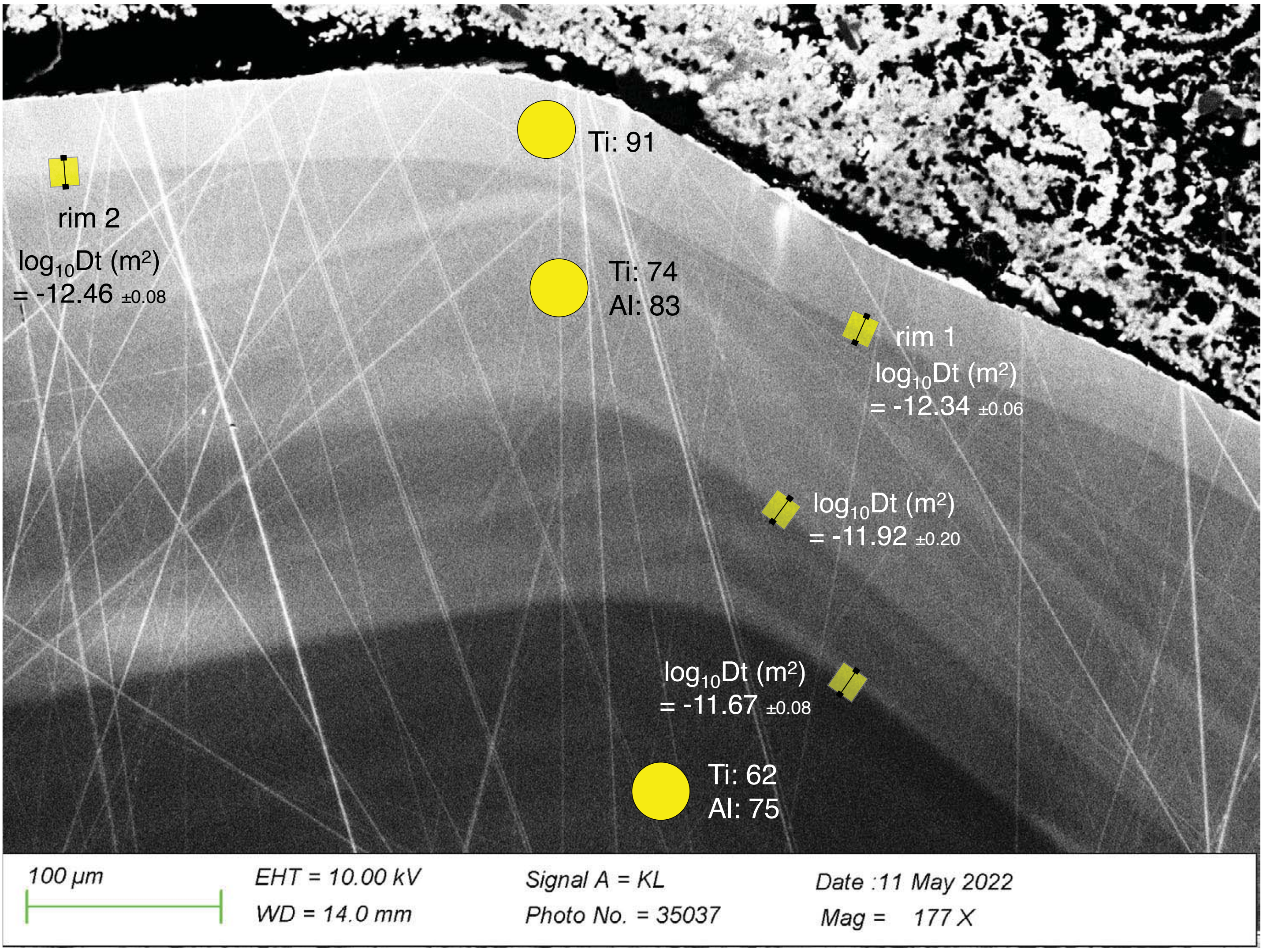

Ti: 91
rim 2
log10Dt (m2)
= -12.46 ±0.08
Ti: 74
Al: 83
rim 1
log10Dt (m2)
= -12.34 ±0.06
log10Dt (m2)
= -11.92 ±0.20
log10Dt (m2)
= -11.67 ±0.08
Ti: 62
Al: 75
100 µm
EHT = 10.00 kV
WD = 14.0 mm
Signal A = KL
Photo No. = 35037
Date :11 May 2022
Mag = 177 X

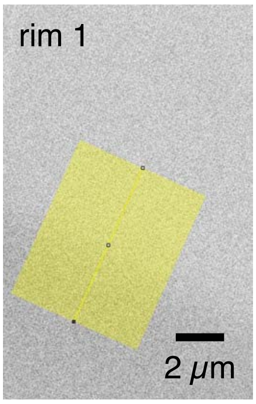

rim 1
2 µm

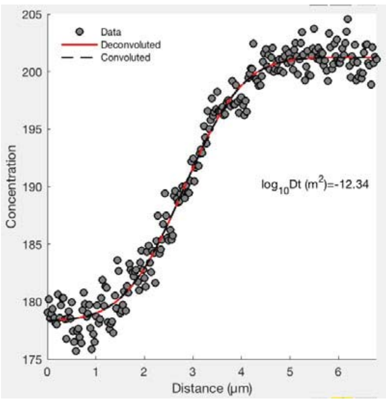

Data
Deconvoluted
Convoluted
Concentration
Distance (µm)
log10Dt (m2)=-12.34

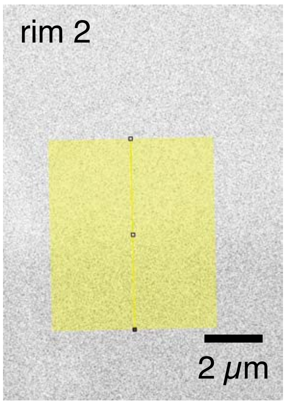

rim 2
2 µm

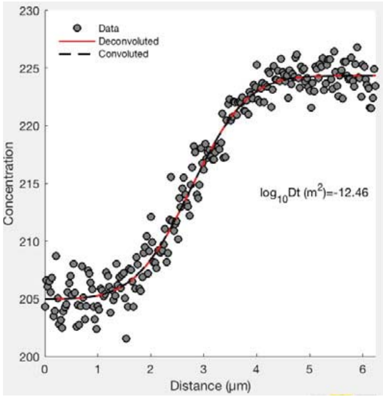

Data
Deconvoluted
Convoluted
Concentration
Distance (µm)
log10Dt (m2)=-12.46

Hiko1 Q9

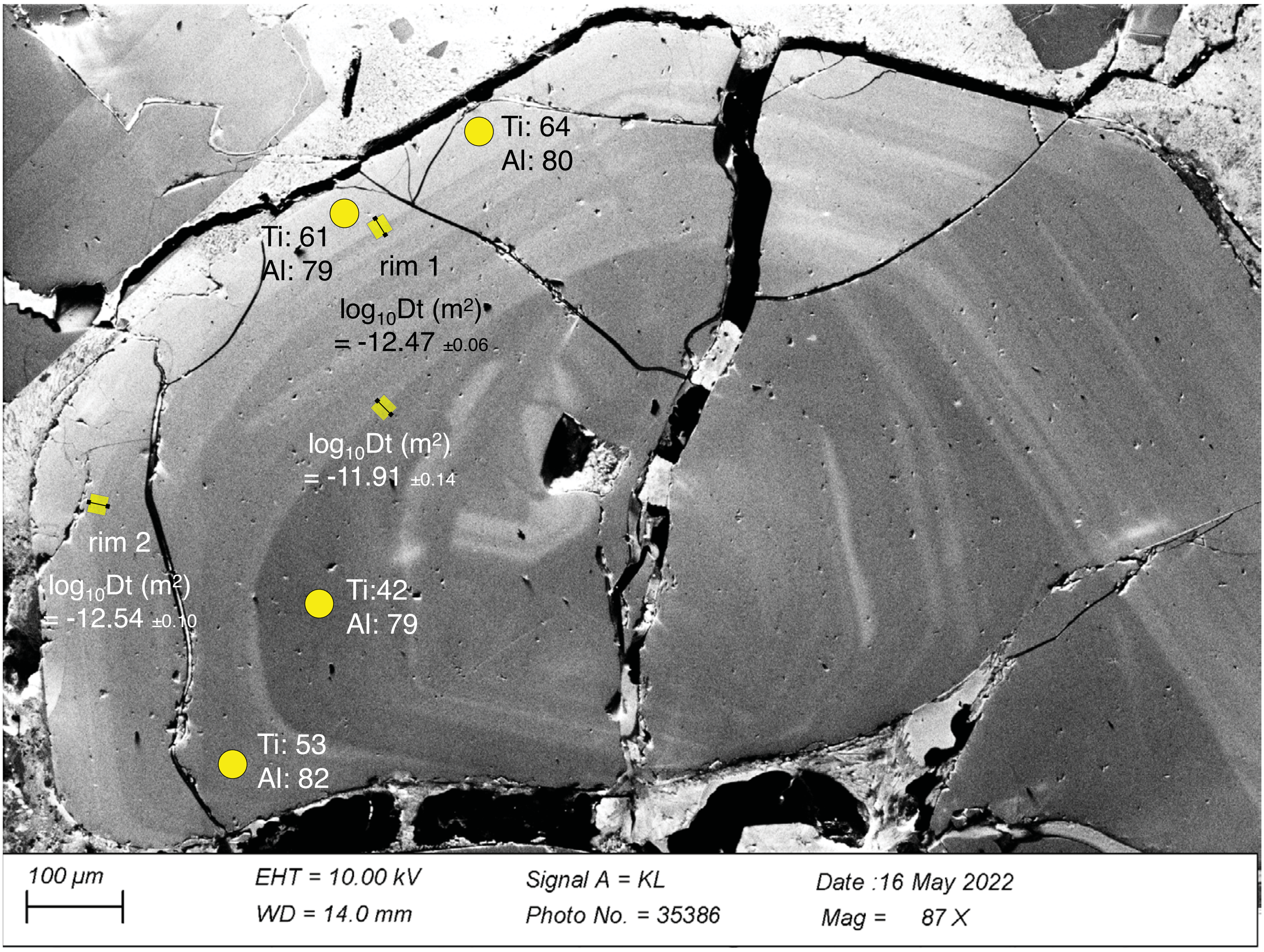
Ti: 64
Al: 80
Ti: 61
Al: 79
rim 1
log10Dt (m2)
= -12.47 ±0.06
log10Dt (m2)
= -11.91 ±0.14
rim 2
log10Dt (m2)
= -12.54 ±0.10
Ti:42
Al: 79
Ti: 53
Al: 82
100 μm
EHT = 10.00 kV
WD = 14.0 mm
Signal A = KL
Photo No. = 35386
Date :16 May 2022
Mag = 87 X

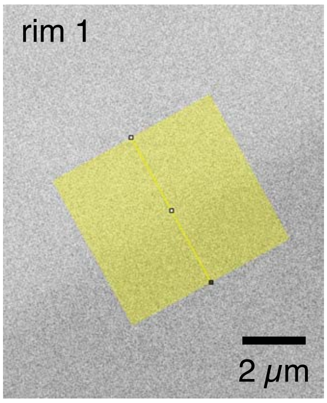
rim 1
2 μm

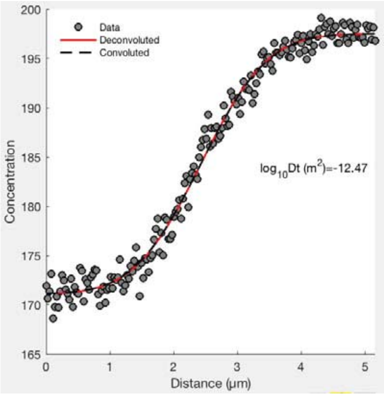
Data
Deconvoluted
Convoluted
Concentration
Distance (μm)
log10Dt (m2)=-12.47

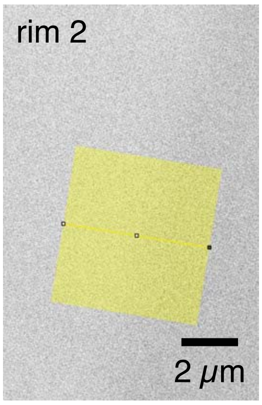
rim 2
2 μm

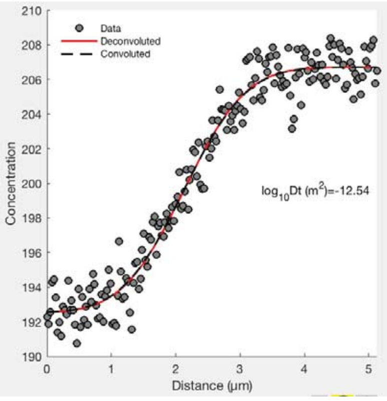
Data
Deconvoluted
Convoluted
Concentration
Distance (μm)
log10Dt (m2)=-12.54

Hiko1 Q10

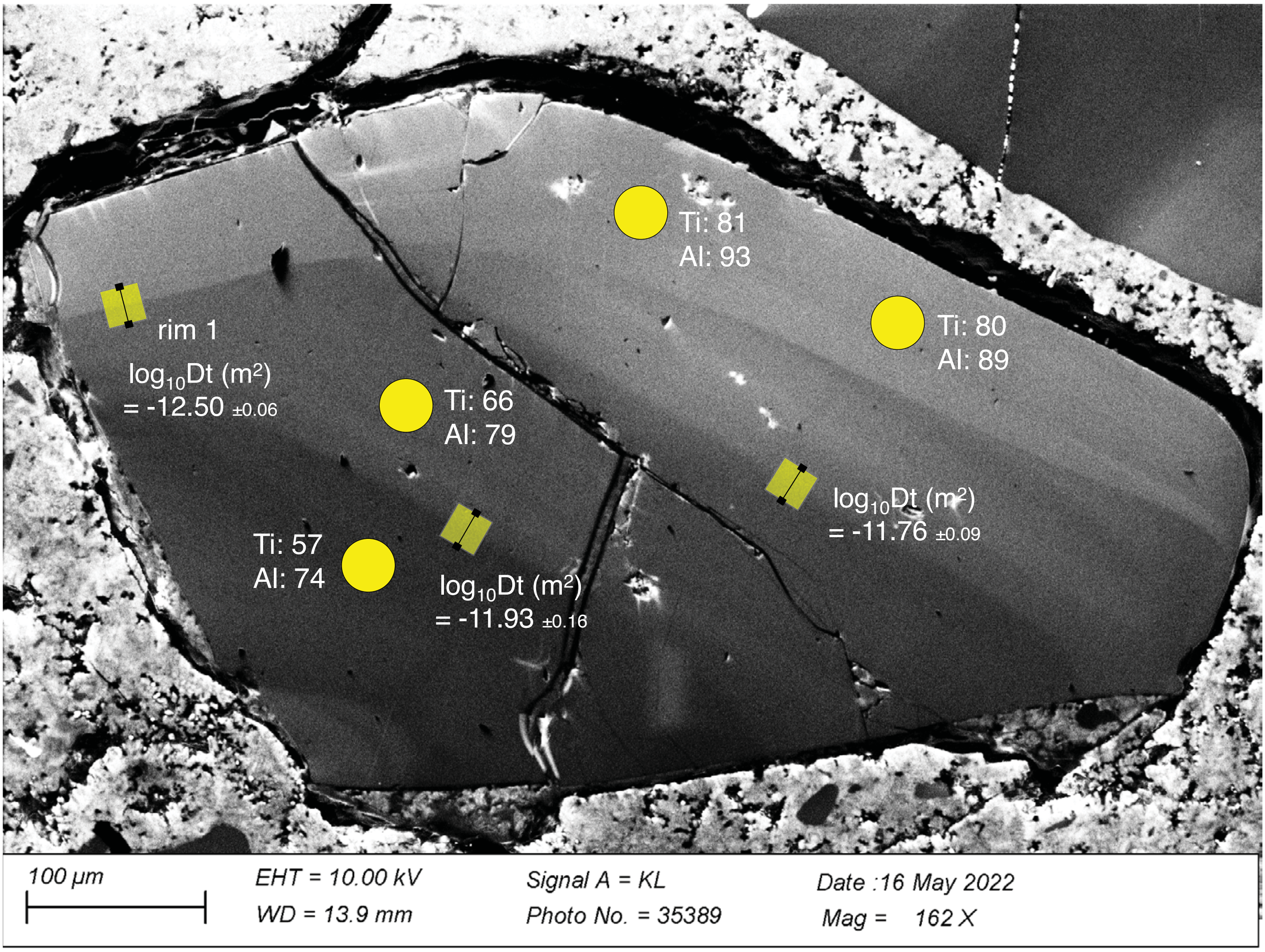

rim 1
log10Dt (m2)
= -12.50 ±0.06
Ti: 81
Al: 93
Ti: 80
Al: 89
Ti: 66
Al: 79
Ti: 57
Al: 74
log10Dt (m2)
= -11.93 ±0.16
log10Dt (m2)
= -11.76 ±0.09
100 µm
EHT = 10.00 kV
WD = 13.9 mm
Signal A = KL
Photo No. = 35389
Date :16 May 2022
Mag = 162 X

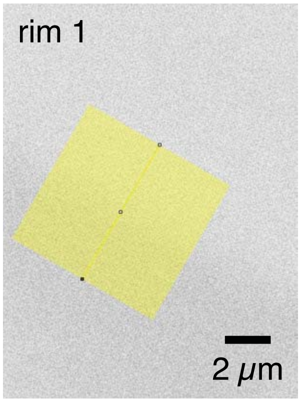

rim 1
2 µm

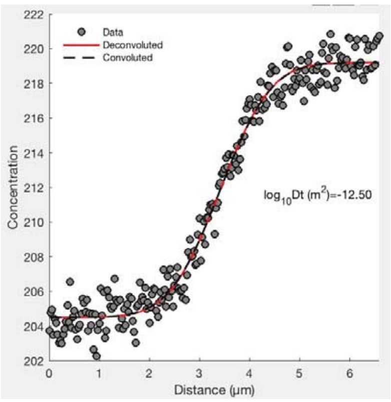

Data
Deconvoluted
Convoluted
log10Dt (m2)=-12.50
Concentration
Distance (µm)

Lassen Q1

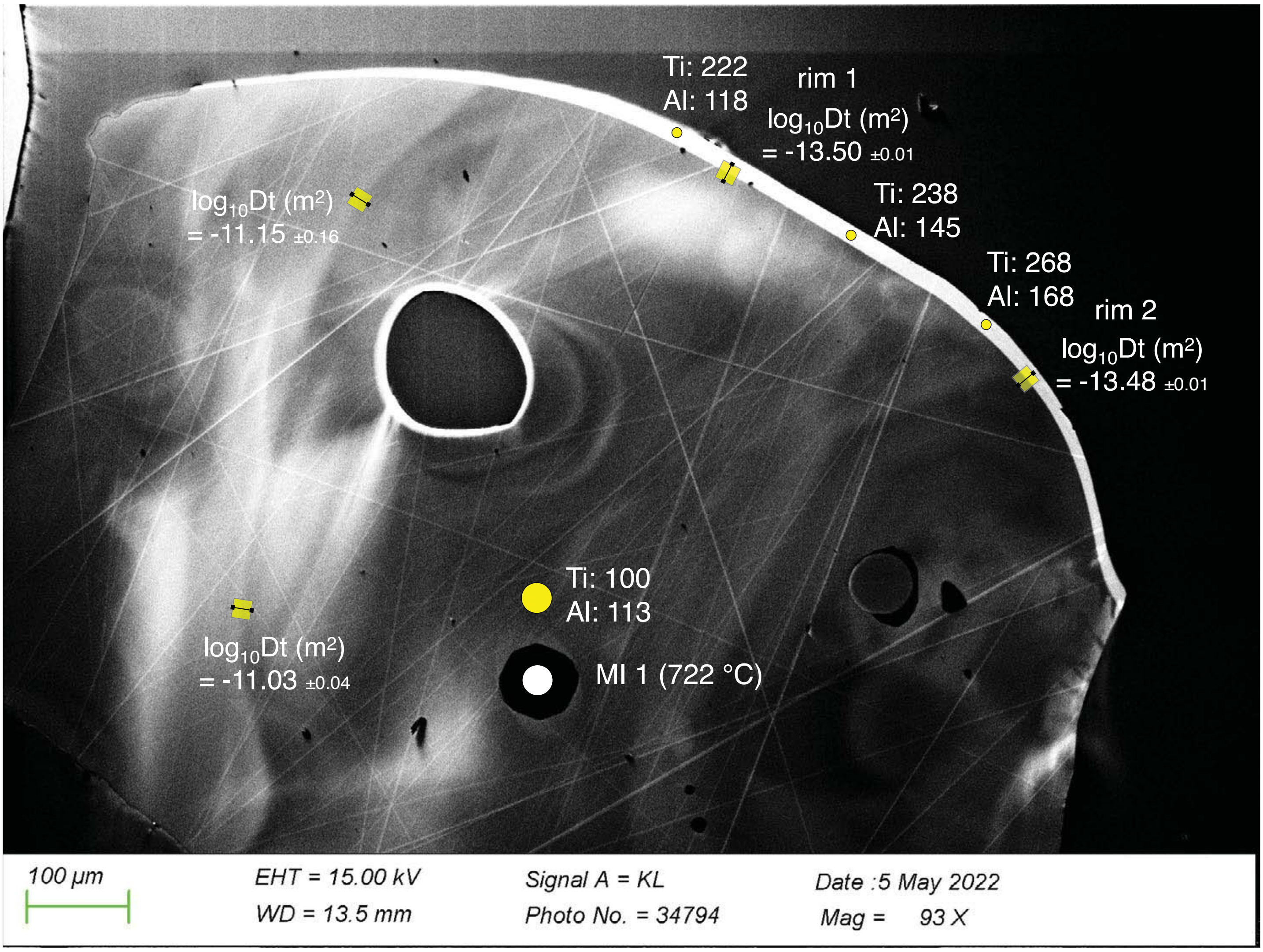
Ti: 222
Al: 118
rim 1
$\log_{10}Dt$ (m^2)
= -13.50 ±0.01
$\log_{10}Dt$ (m^2)
= -11.15 ±0.16
Ti: 238
Al: 145
Ti: 268
Al: 168
rim 2
$\log_{10}Dt$ (m^2)
= -13.48 ±0.01
Ti: 100
Al: 113
$\log_{10}Dt$ (m^2)
= -11.03 ±0.04
MI 1 (722 °C)
100 μm
EHT = 15.00 kV
WD = 13.5 mm
Signal A = KL
Photo No. = 34794
Date :5 May 2022
Mag = 93 X

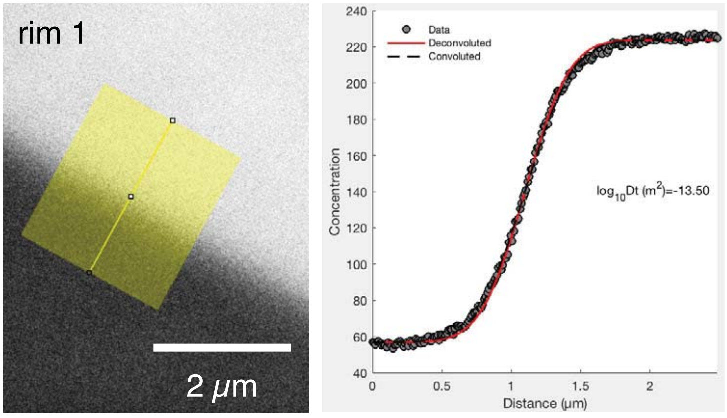
rim 1
2 μm
Data
Deconvoluted
Convoluted
Concentration
Distance (μm)
$\log_{10}Dt$ (m^2)=-13.50

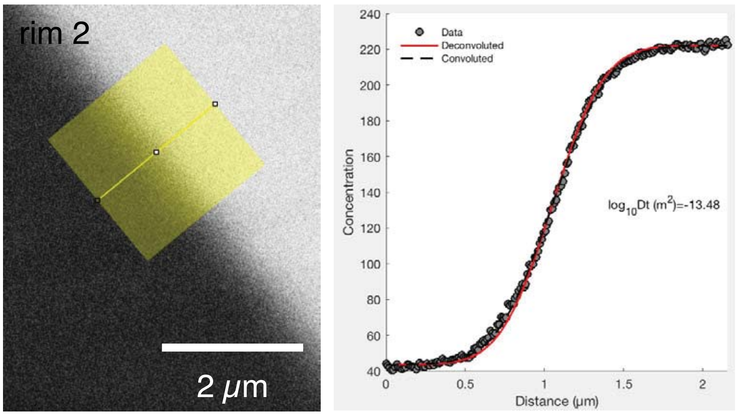
rim 2
2 μm
Data
Deconvoluted
Convoluted
Concentration
Distance (μm)
$\log_{10}Dt$ (m^2)=-13.48

Lassen Q2

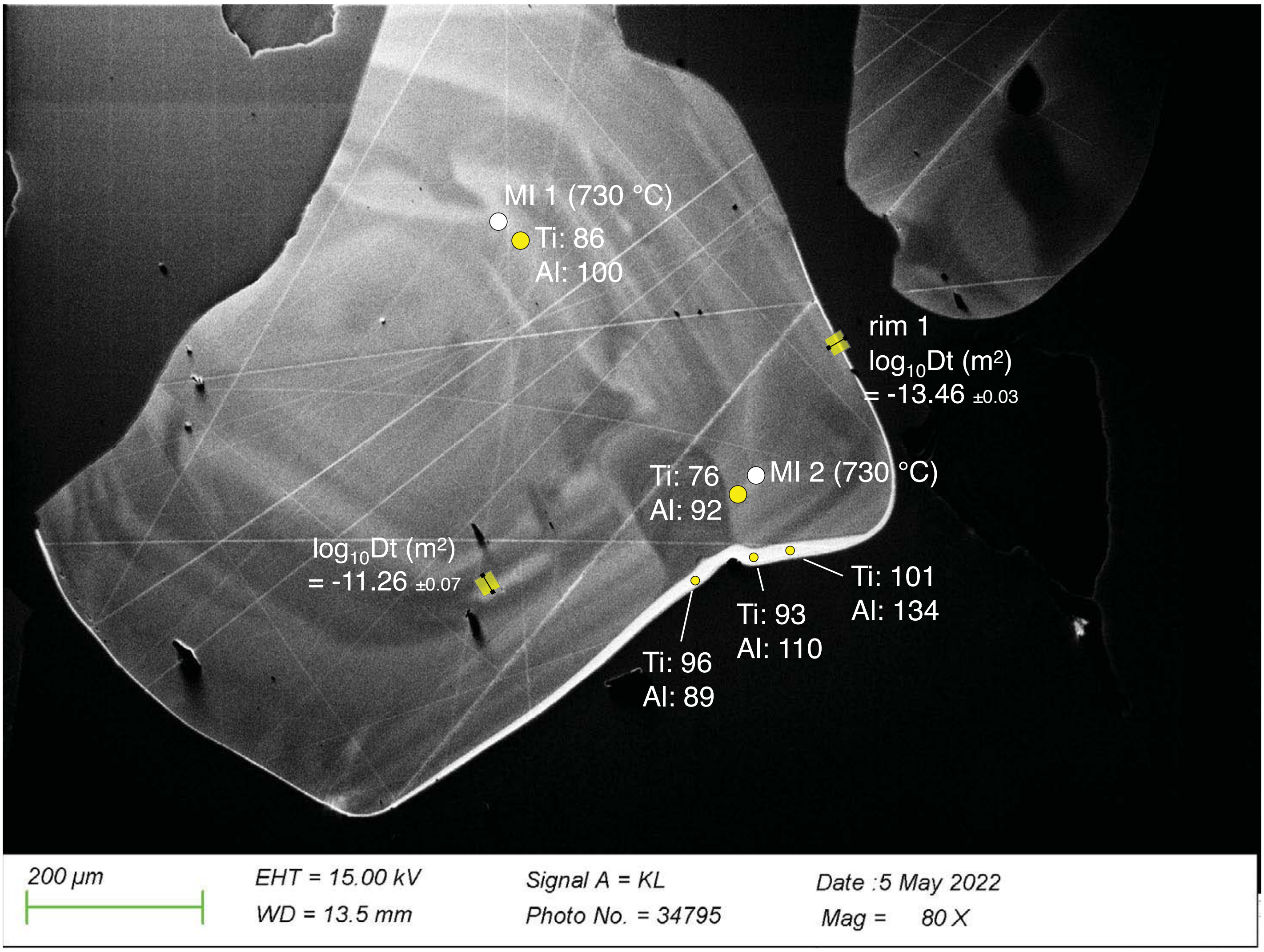
MI 1 (730 °C)
Ti: 86
Al: 100
rim 1
$log_{10}Dt$ (m^2)
= -13.46 ±0.03
Ti: 76
Al: 92
MI 2 (730 °C)
$log_{10}Dt$ (m^2)
= -11.26 ±0.07
Ti: 101
Al: 134
Ti: 93
Al: 110
Ti: 96
Al: 89
200 μm
EHT = 15.00 kV
WD = 13.5 mm
Signal A = KL
Photo No. = 34795
Date :5 May 2022
Mag = 80 X

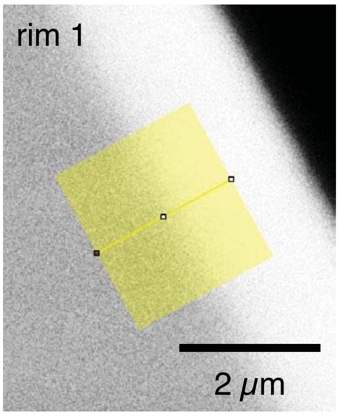
rim 1
2 μm

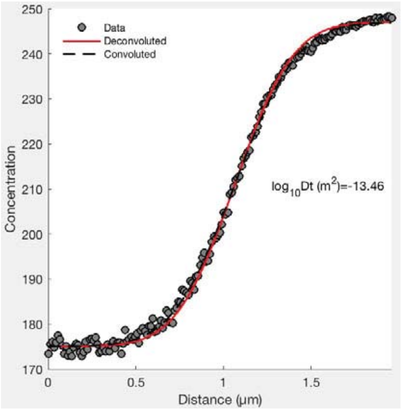
Data
Deconvoluted
Convoluted
Concentration
Distance (μm)
$log_{10}Dt$ (m^2)=-13.46
250
240
230
220
210
200
190
180
170
0
0.5
1
1.5

Rainbow1 A1

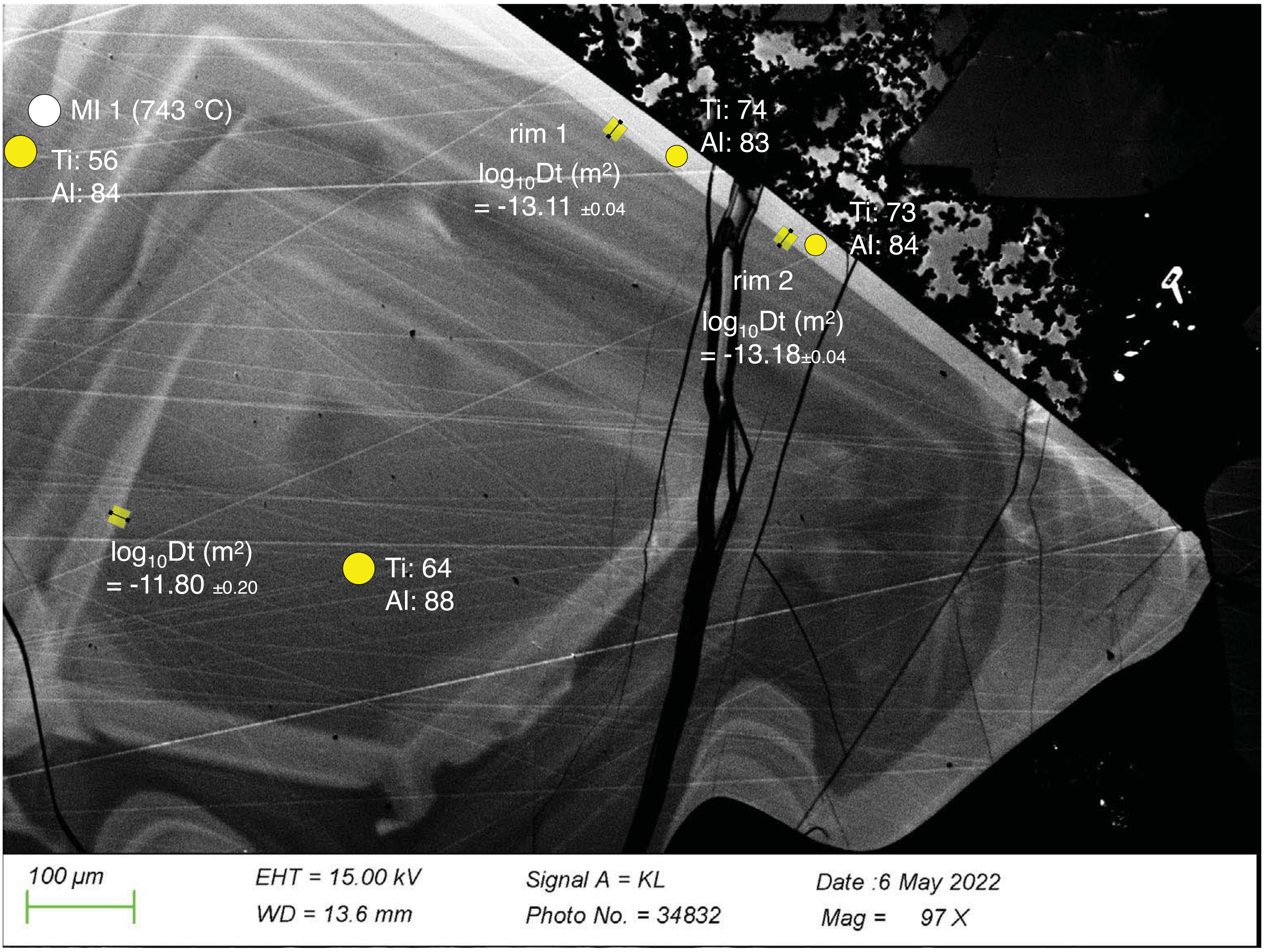

MI 1 (743 °C)
Ti: 56
Al: 84
rim 1
log10Dt (m2)
= -13.11 ±0.04
Ti: 74
Al: 83
Ti: 73
Al: 84
rim 2
log10Dt (m2)
= -13.18±0.04
log10Dt (m2)
= -11.80 ±0.20
Ti: 64
Al: 88
100 μm
EHT = 15.00 kV
WD = 13.6 mm
Signal A = KL
Photo No. = 34832
Date :6 May 2022
Mag = 97 X

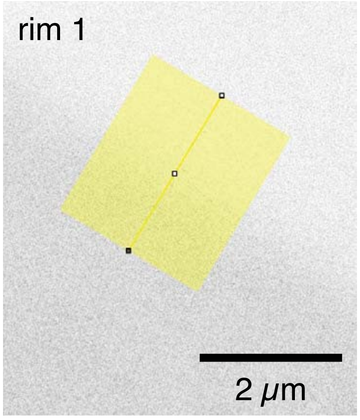

rim 1
2 μm

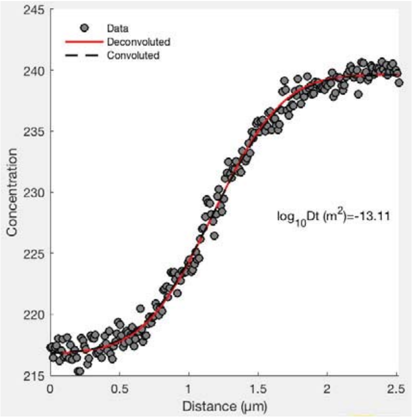

Data
Deconvoluted
Convoluted
Concentration
Distance (μm)
log10Dt (m2)=-13.11

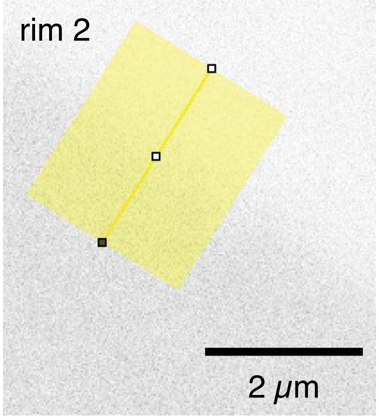

rim 2
2 μm

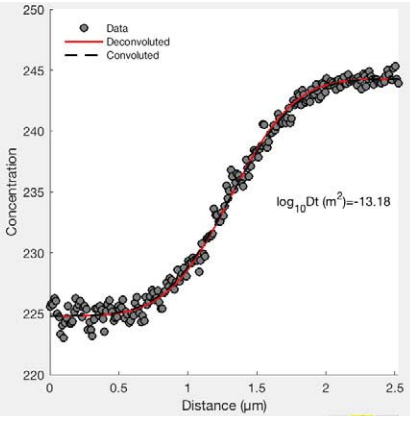

Data
Deconvoluted
Convoluted
Concentration
Distance (μm)
log10Dt (m2)=-13.18

Rainbow1 A3

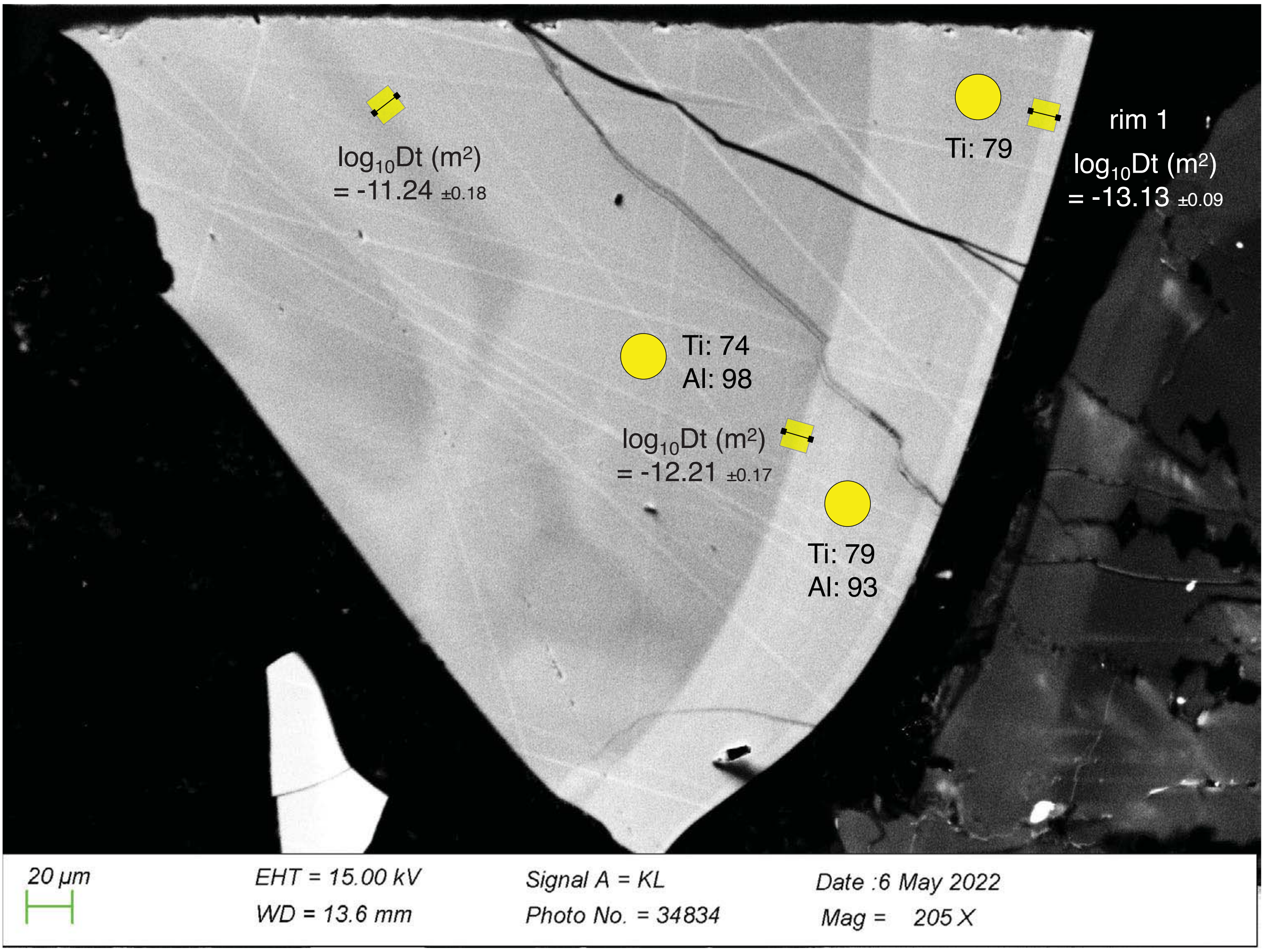

log$_{10}$Dt (m^2)
= -11.24 ±0.18
Ti: 79
rim 1
log$_{10}$Dt (m^2)
= -13.13 ±0.09
Ti: 74
Al: 98
log$_{10}$Dt (m^2)
= -12.21 ±0.17
Ti: 79
Al: 93
20 µm
EHT = 15.00 kV
WD = 13.6 mm
Signal A = KL
Photo No. = 34834
Date :6 May 2022
Mag = 205 X

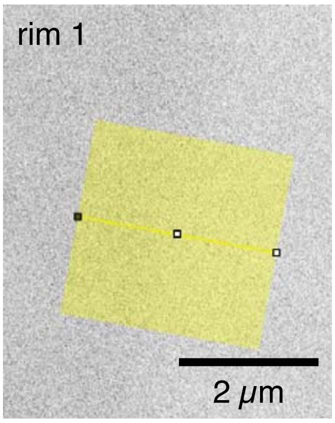

rim 1
2 µm

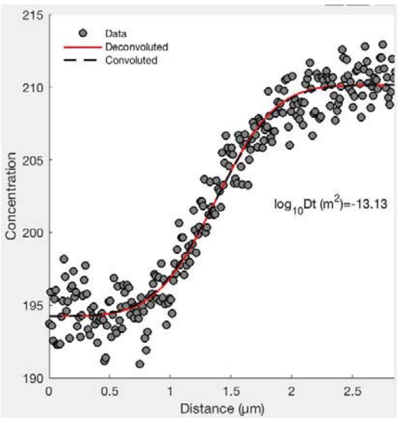

Data
Deconvoluted
Convoluted
log$_{10}$Dt (m^2)=-13.13
Concentration
Distance (µm)
215
210
205
200
195
190
0
0.5
1
1.5
2
2.5

Rainbow1 C1

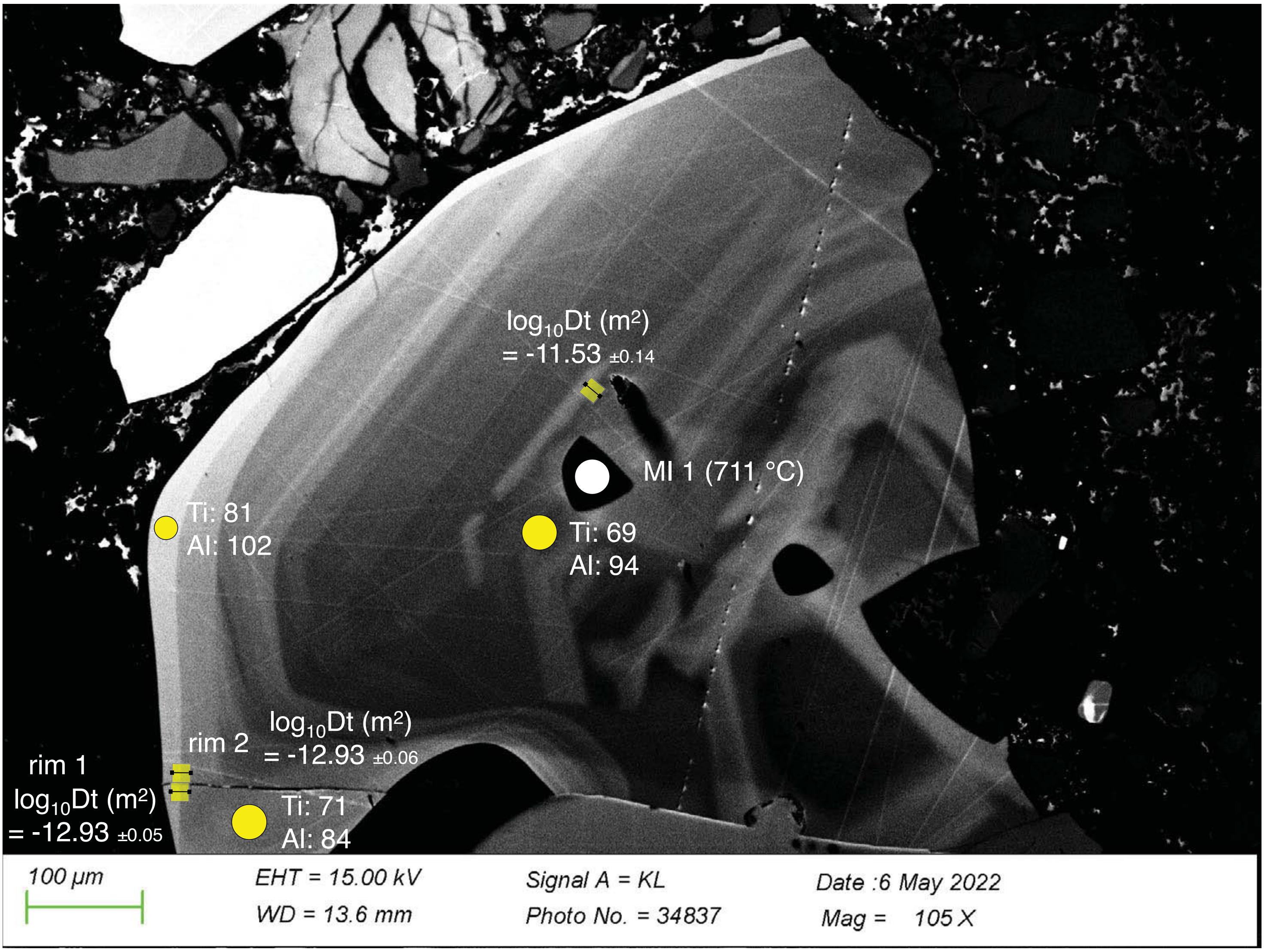
log10Dt (m2)
= -11.53 ±0.14
MI 1 (711 °C)
Ti: 81
Al: 102
Ti: 69
Al: 94
log10Dt (m2)
= -12.93 ±0.06
rim 2
rim 1
log10Dt (m2)
= -12.93 ±0.05
Ti: 71
Al: 84
100 μm
EHT = 15.00 kV
WD = 13.6 mm
Signal A = KL
Photo No. = 34837
Date :6 May 2022
Mag = 105 X

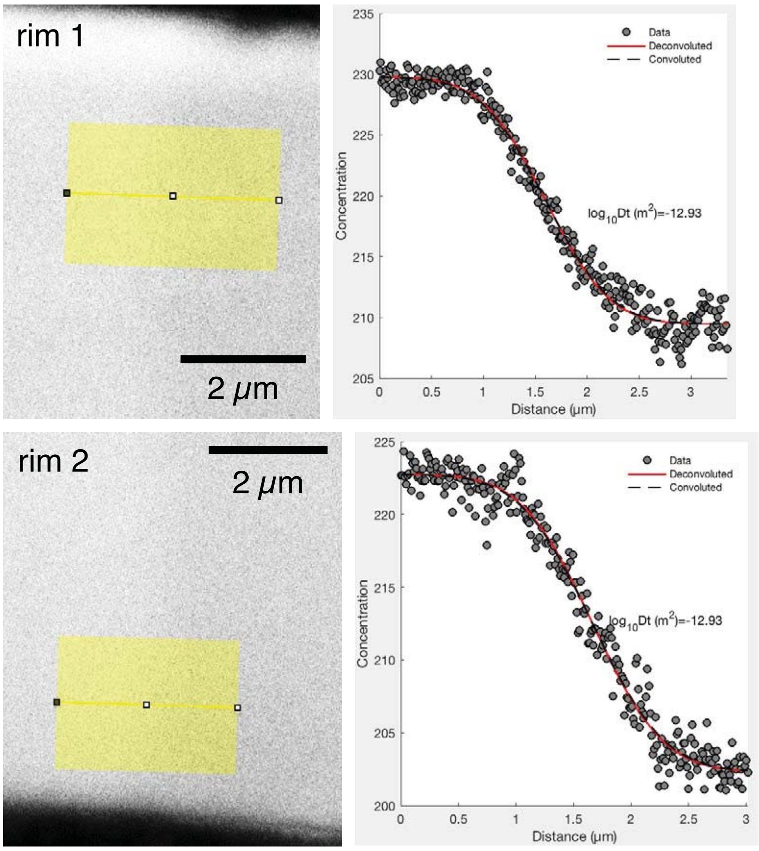
rim 1
2 μm
Data
Deconvoluted
Convoluted
log10Dt (m2)=-12.93
Concentration
Distance (μm)
rim 2
2 μm
Data
Deconvoluted
Convoluted
log10Dt (m2)=-12.93
Concentration
Distance (μm)

Toba 3C

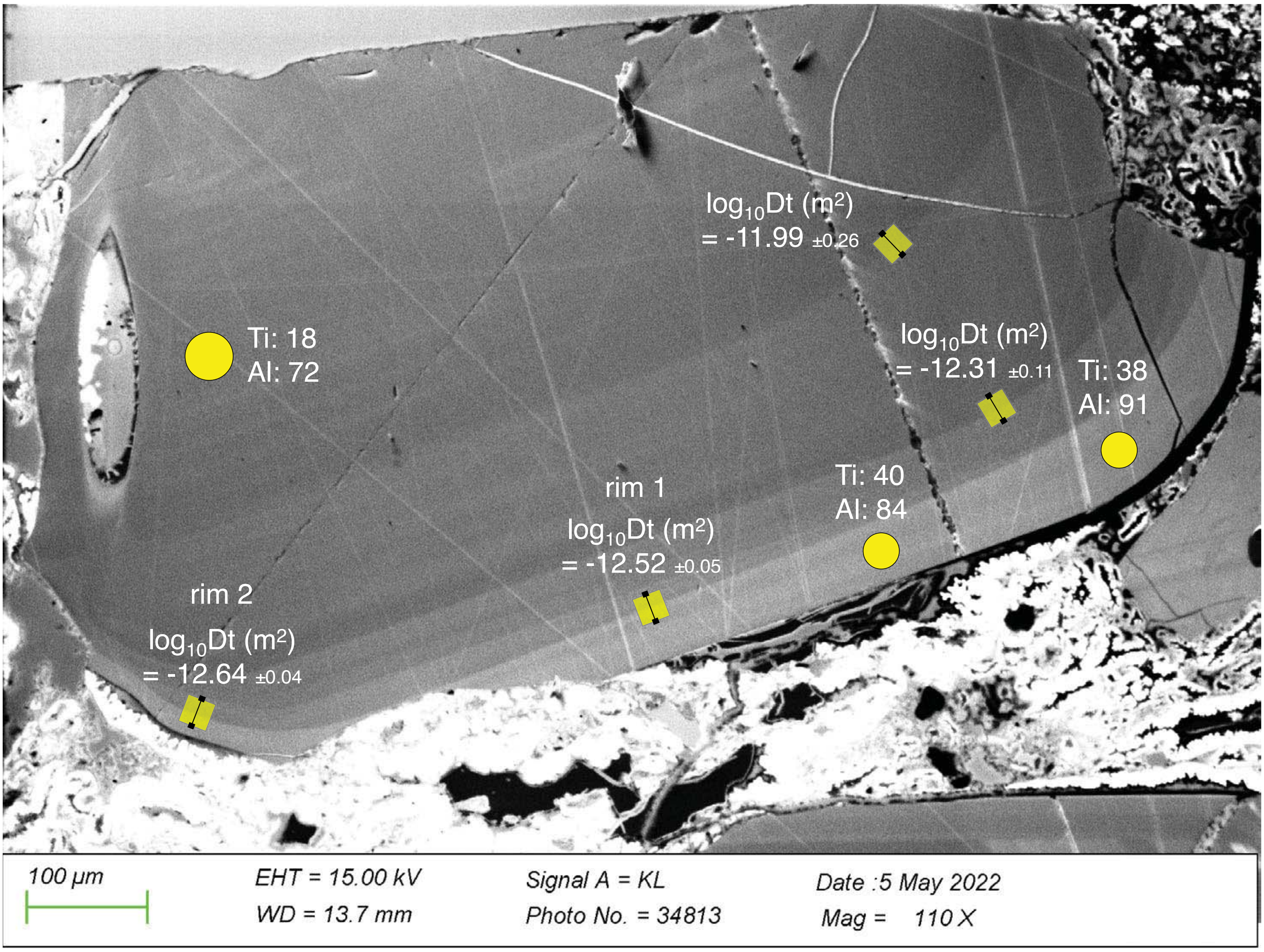

log10Dt (m2)
= -11.99 ±0.26
Ti: 18
Al: 72
log10Dt (m2)
= -12.31 ±0.11
Ti: 38
Al: 91
rim 1
log10Dt (m2)
= -12.52 ±0.05
Ti: 40
Al: 84
rim 2
log10Dt (m2)
= -12.64 ±0.04
100 µm
EHT = 15.00 kV
WD = 13.7 mm
Signal A = KL
Photo No. = 34813
Date :5 May 2022
Mag = 110 X

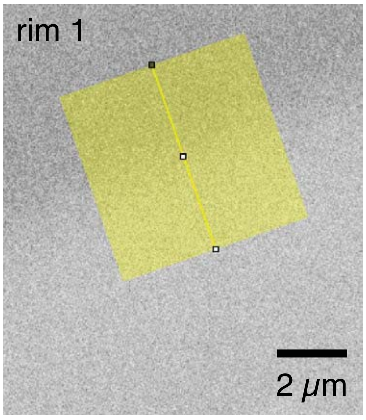

rim 1
2 µm

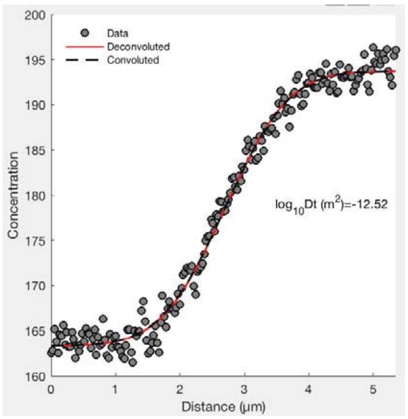

Data
Deconvoluted
Convoluted
Concentration
Distance (µm)
log10Dt (m2)=-12.52

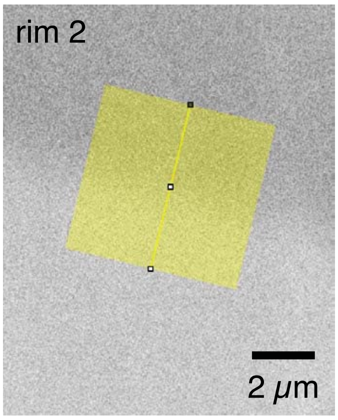

rim 2
2 µm

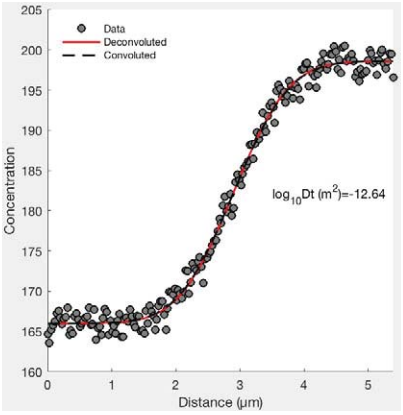

Data
Deconvoluted
Convoluted
Concentration
Distance (µm)
log10Dt (m2)=-12.64

Toba A1

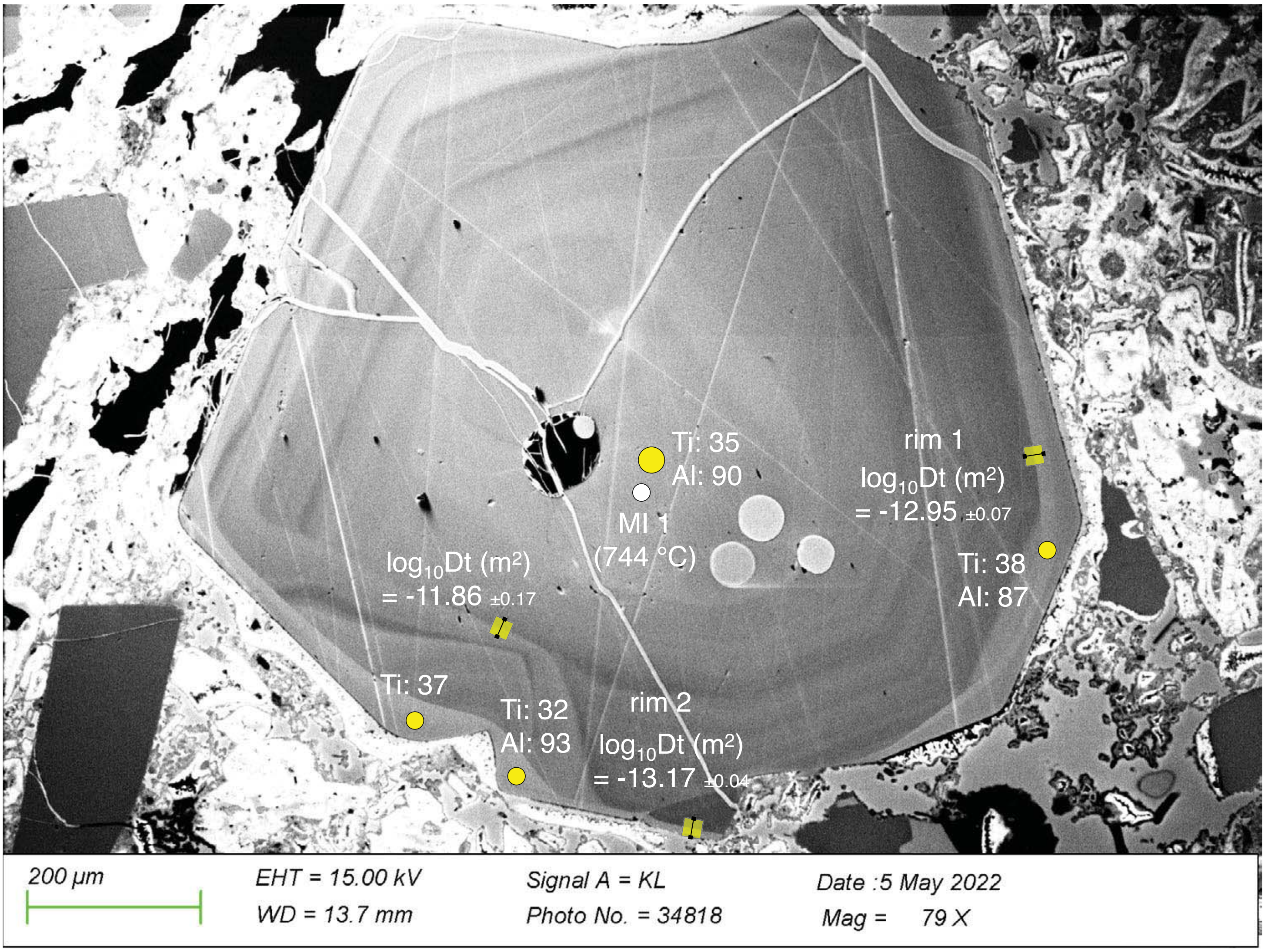

Ti: 35
Al: 90
MI 1
(744 °C)
rim 1
log10Dt (m2)
= -12.95 ±0.07
Ti: 38
Al: 87
log10Dt (m2)
= -11.86 ±0.17
Ti: 37
Ti: 32
Al: 93
rim 2
log10Dt (m2)
= -13.17 ±0.04
200 μm
EHT = 15.00 kV
WD = 13.7 mm
Signal A = KL
Photo No. = 34818
Date :5 May 2022
Mag = 79 X

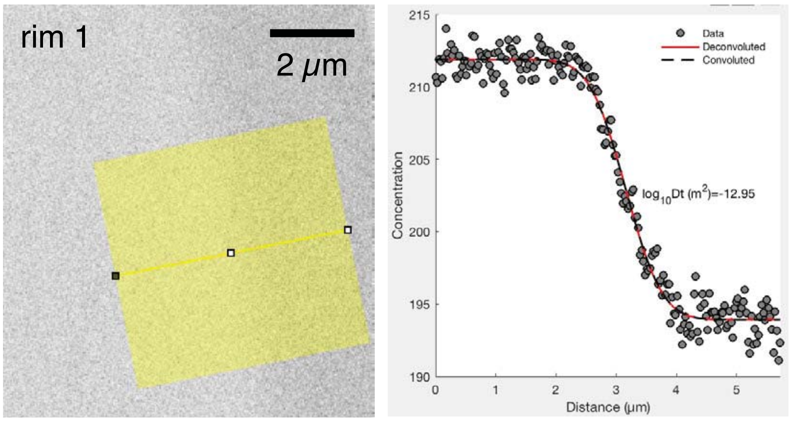

rim 1
2 μm
Data
Deconvoluted
Convoluted
log10Dt (m2)=-12.95
Concentration
Distance (μm)

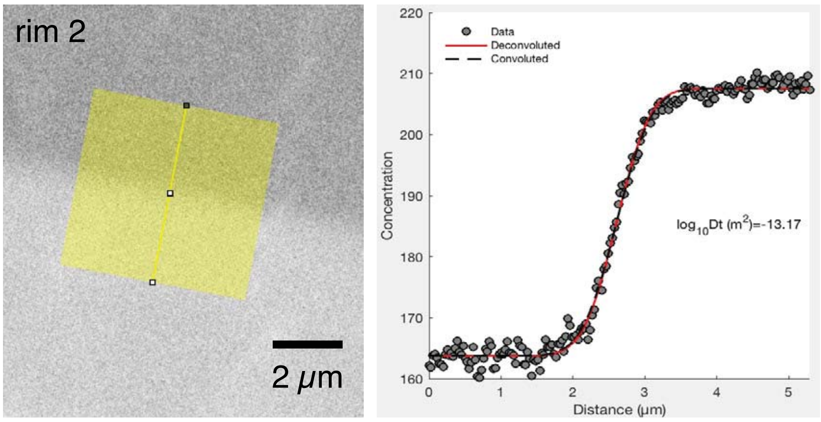

rim 2
2 μm
Data
Deconvoluted
Convoluted
log10Dt (m2)=-13.17
Concentration
Distance (μm)

Toba A2

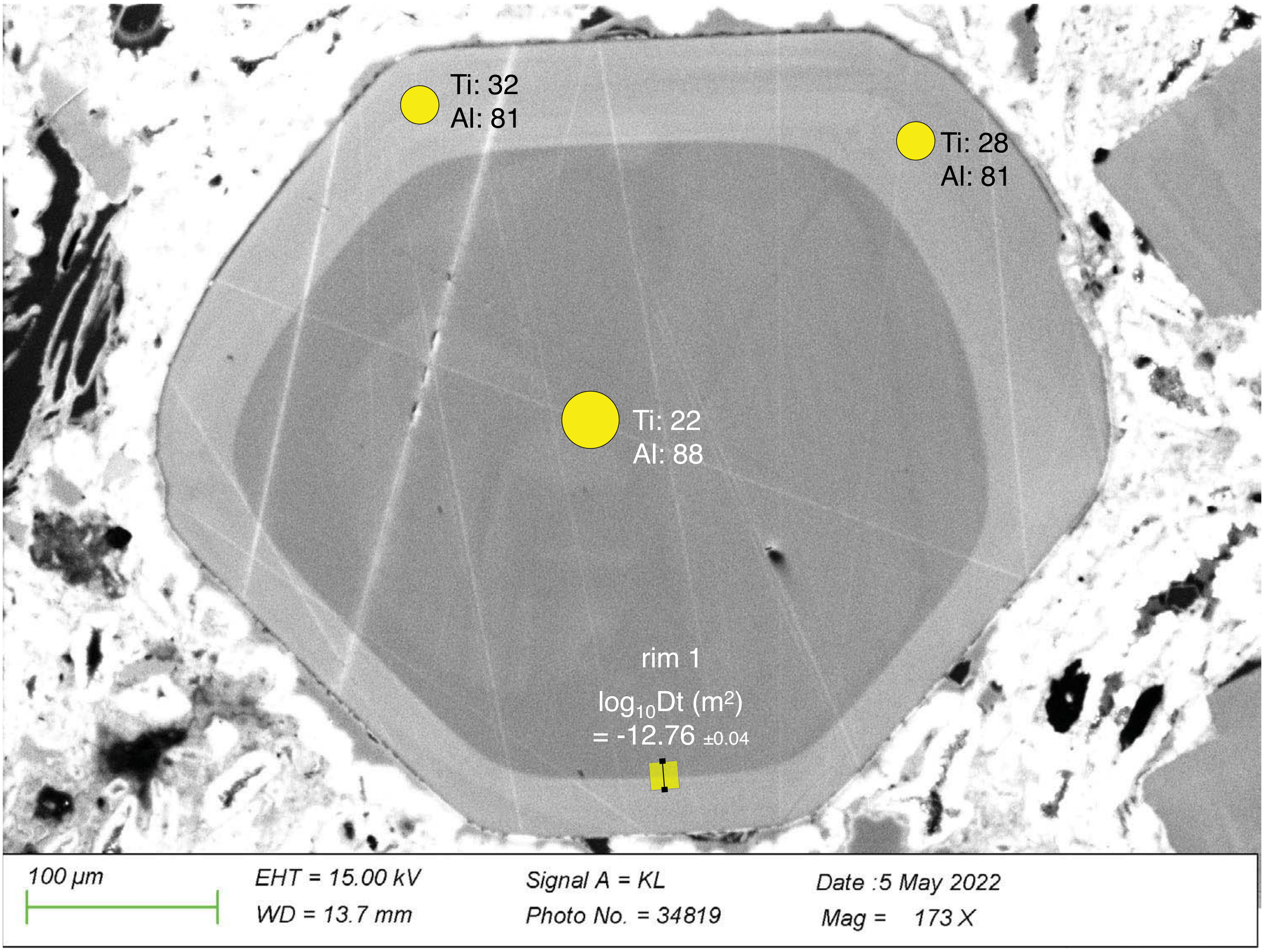

Ti: 32
Al: 81
Ti: 28
Al: 81
Ti: 22
Al: 88
rim 1
log10Dt (m2)
= -12.76 ±0.04
100 µm
EHT = 15.00 kV
WD = 13.7 mm
Signal A = KL
Photo No. = 34819
Date :5 May 2022
Mag = 173 X

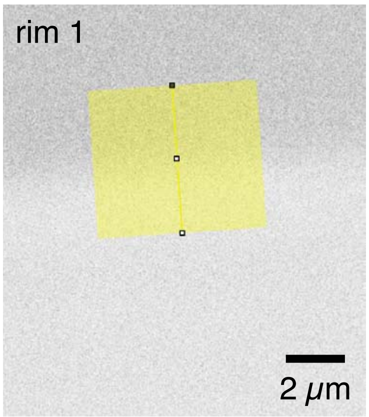

rim 1
2 µm

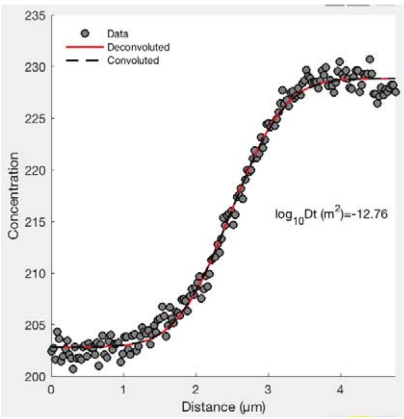

Data
Deconvoluted
Convoluted
log10Dt (m2)=-12.76
Concentration
Distance (µm)

Toba E2

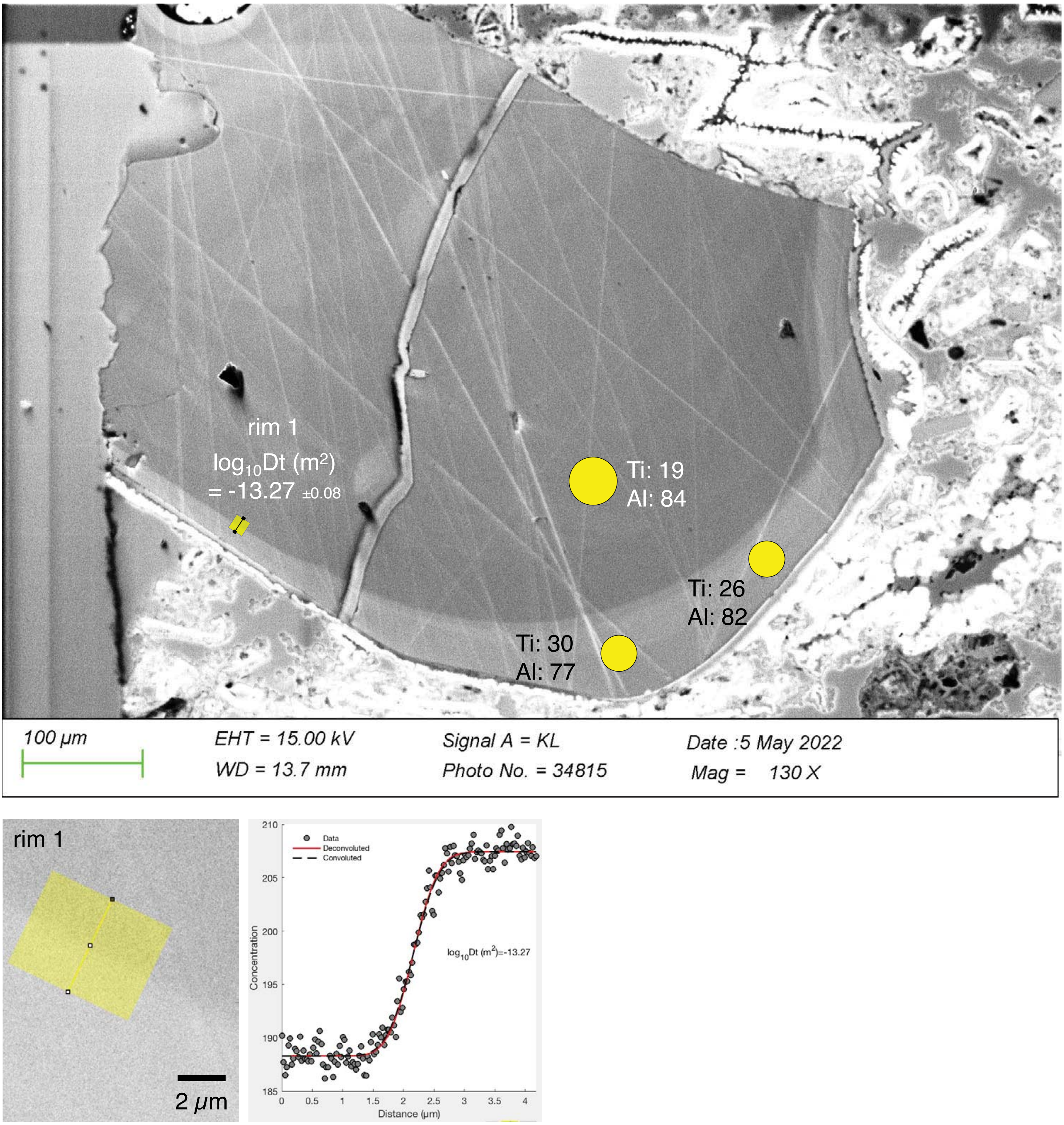
rim 1
log10Dt (m2)
= -13.27 ±0.08
Ti: 19
Al: 84
Ti: 26
Al: 82
Ti: 30
Al: 77
100 µm
EHT = 15.00 kV
WD = 13.7 mm
Signal A = KL
Photo No. = 34815
Date :5 May 2022
Mag = 130 X
rim 1
2 µm
Data
Deconvoluted
Convoluted
log10Dt (m2)=-13.27
Concentration
Distance (µm)
185
190
195
200
205
210
0
0.5
1
1.5
2
2.5
3
3.5
4

Tunnel D2

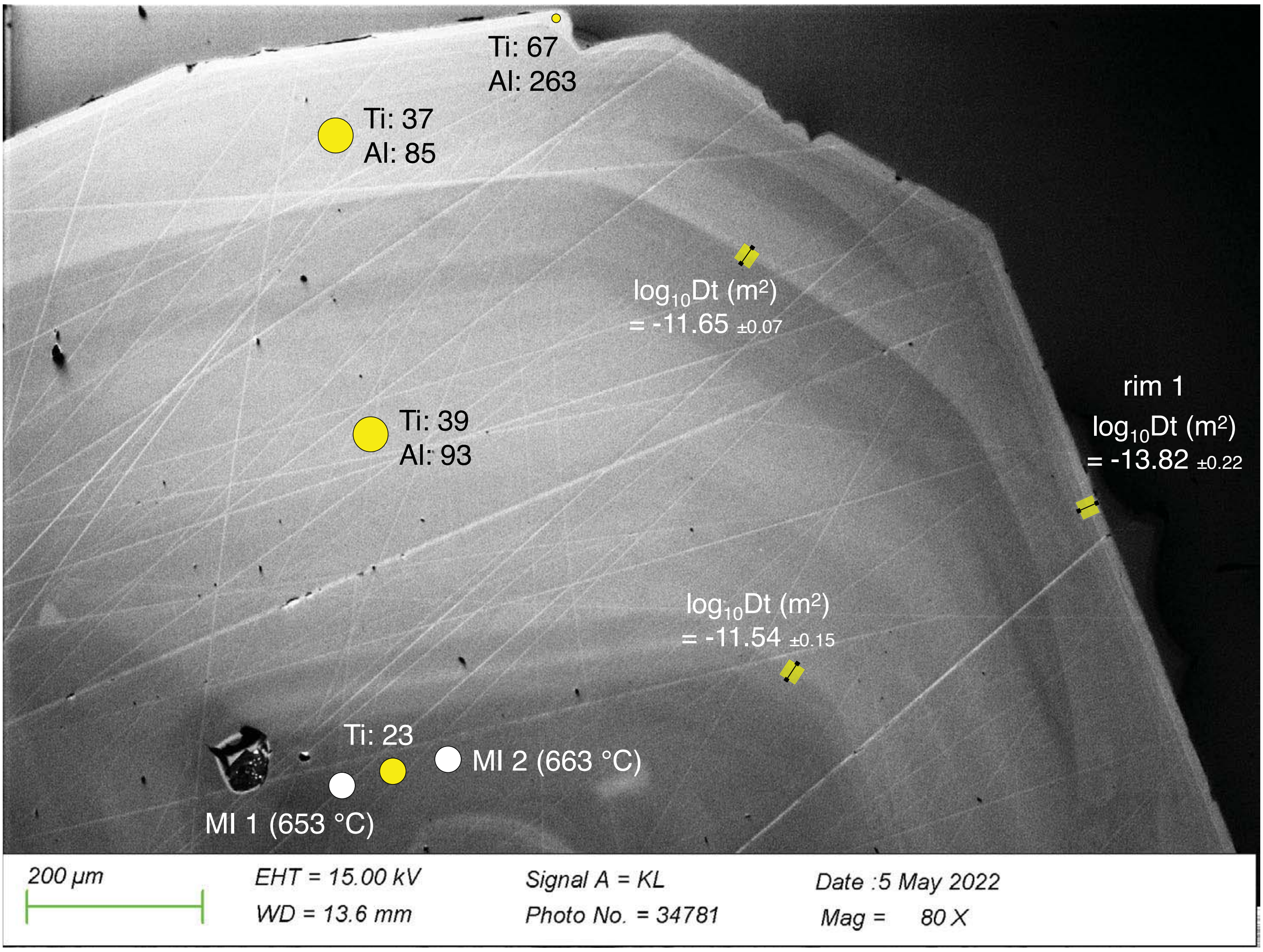
Ti: 67
Al: 263
Ti: 37
Al: 85
log10Dt (m2)
= -11.65 ±0.07
rim 1
log10Dt (m2)
= -13.82 ±0.22
Ti: 39
Al: 93
log10Dt (m2)
= -11.54 ±0.15
Ti: 23
MI 2 (663 °C)
MI 1 (653 °C)
200 μm
EHT = 15.00 kV
WD = 13.6 mm
Signal A = KL
Photo No. = 34781
Date :5 May 2022
Mag = 80 X

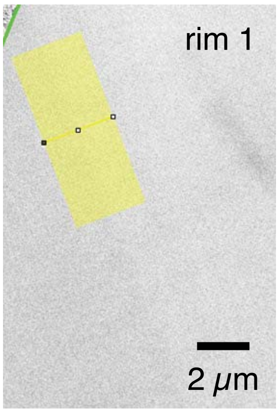
rim 1
2 μm

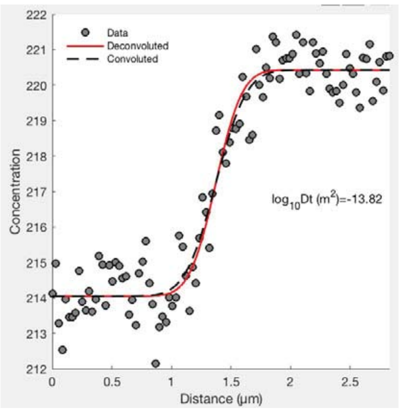
Data
Deconvoluted
Convoluted
Concentration
Distance (μm)
log10Dt (m2)=-13.82

Tunnel2 E1

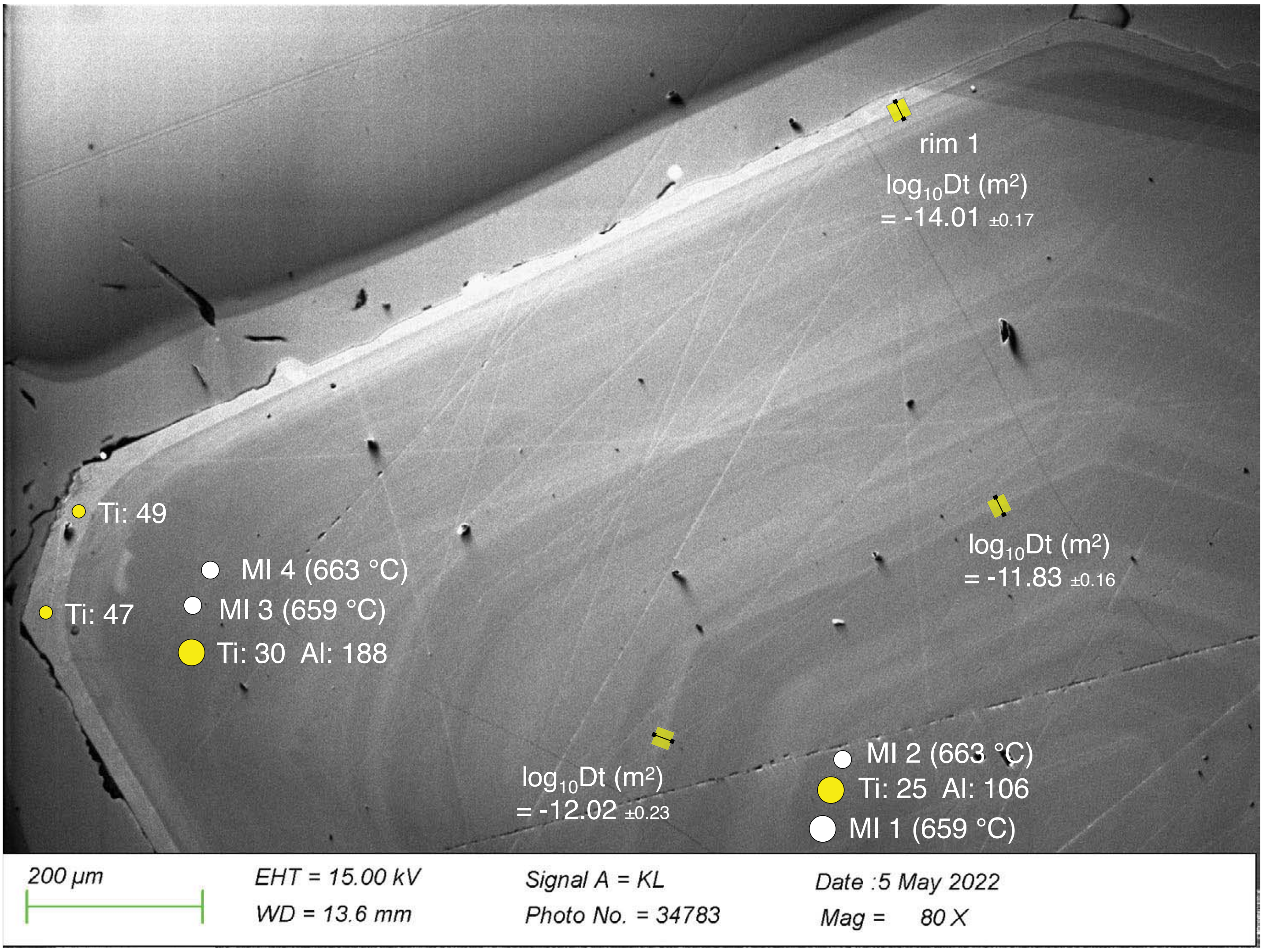
rim 1
log10Dt (m2)
= -14.01 ±0.17
log10Dt (m2)
= -11.83 ±0.16
Ti: 49
MI 4 (663 °C)
MI 3 (659 °C)
Ti: 47
Ti: 30 Al: 188
log10Dt (m2)
= -12.02 ±0.23
MI 2 (663 °C)
Ti: 25 Al: 106
MI 1 (659 °C)
200 µm
EHT = 15.00 kV
WD = 13.6 mm
Signal A = KL
Photo No. = 34783
Date :5 May 2022
Mag = 80 X

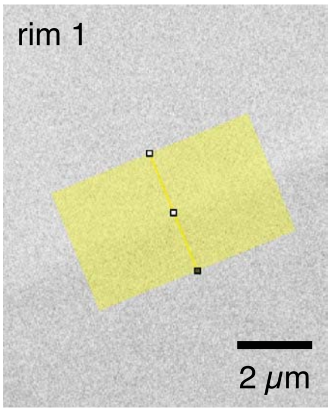
rim 1
2 µm

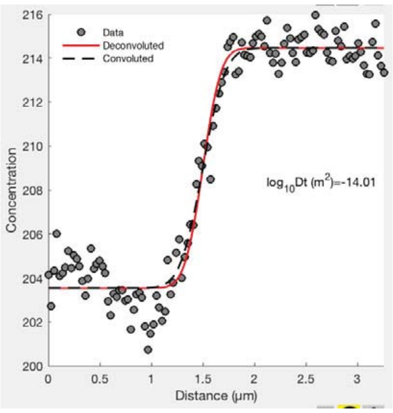
Data
Deconvoluted
Convoluted
Concentration
Distance (µm)
log10Dt (m2)=-14.01

Tunnel2 G1

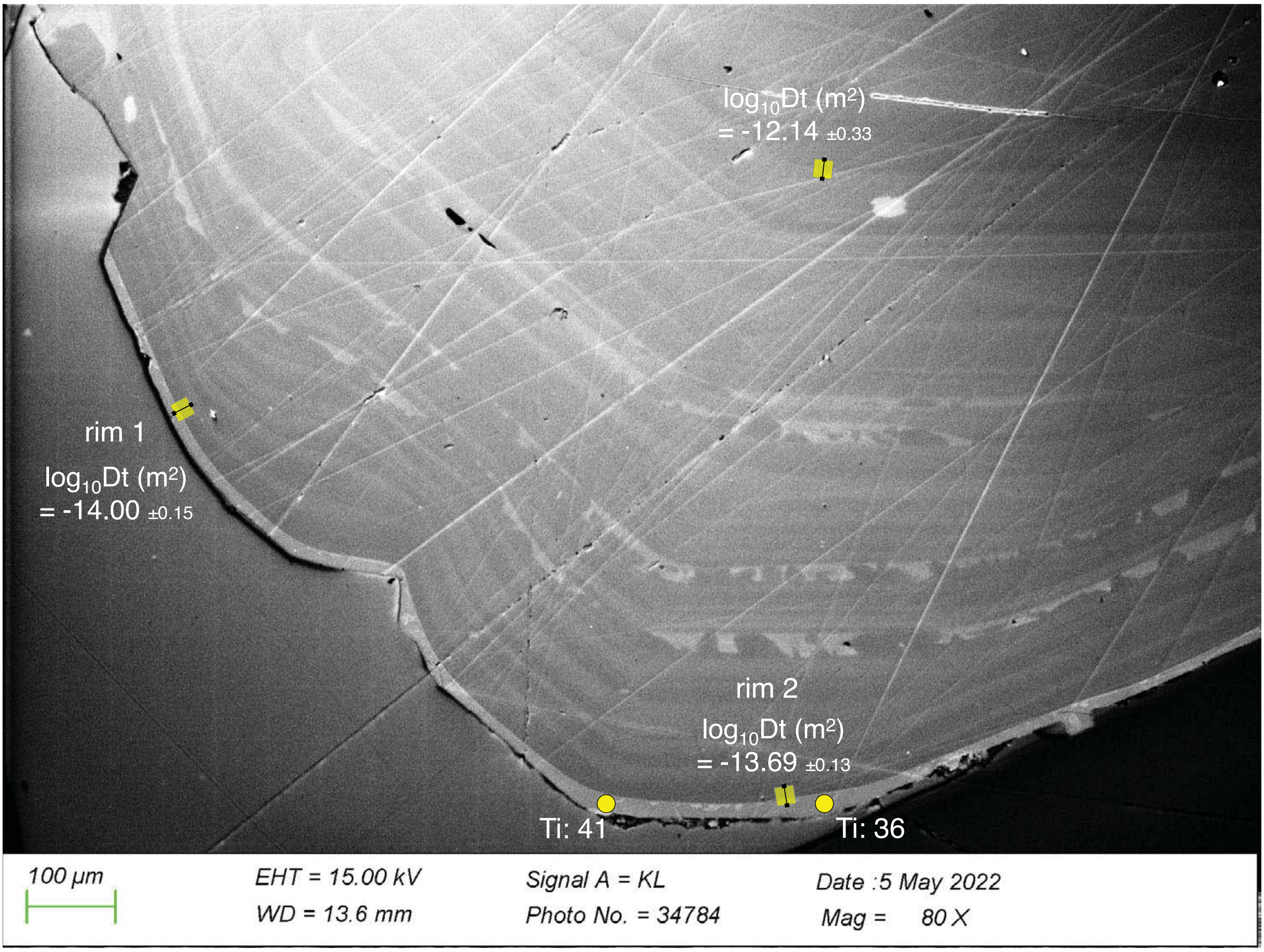

$\log_{10}Dt$ (m²)
= -12.14 ±0.33
rim 1
$\log_{10}Dt$ (m²)
= -14.00 ±0.15
rim 2
$\log_{10}Dt$ (m²)
= -13.69 ±0.13
Ti: 41
Ti: 36
100 µm
EHT = 15.00 kV
WD = 13.6 mm
Signal A = KL
Photo No. = 34784
Date :5 May 2022
Mag = 80 X

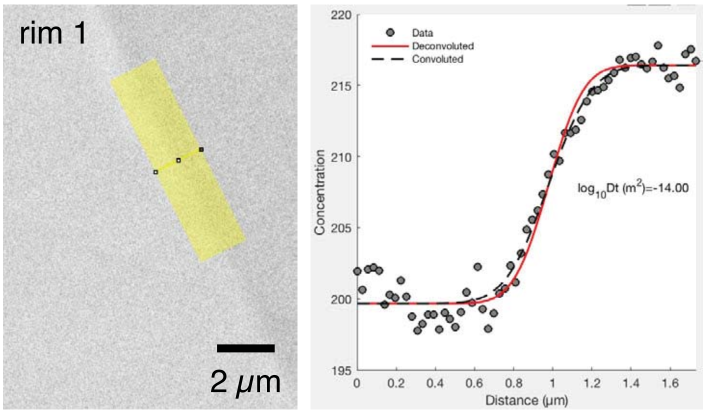

rim 1
2 µm
Data
Deconvoluted
Convoluted
$\log_{10}Dt$ (m²)=-14.00
Concentration
Distance (µm)

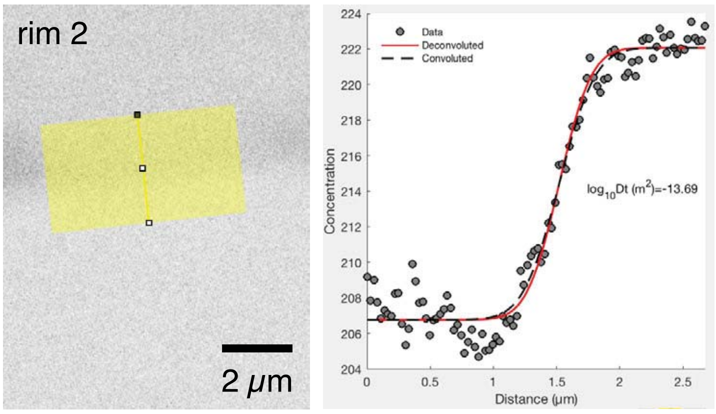

rim 2
2 µm
Data
Deconvoluted
Convoluted
$\log_{10}Dt$ (m²)=-13.69
Concentration
Distance (µm)
